# Supplementary figures and images for: The associations of alcoholic liver disease and nonalcoholic fatty liver disease with bone mineral density and the mediation of serum 25-Hydroxyvitamin D: A bidirectional and two-step Mendelian randomization
Source: PLoS One. 2023 Oct 19;18(10):e0292881. doi: 10.1371/journal.pone.0292881 (PMC10586666; doi:10.1371/journal.pone.0292881)

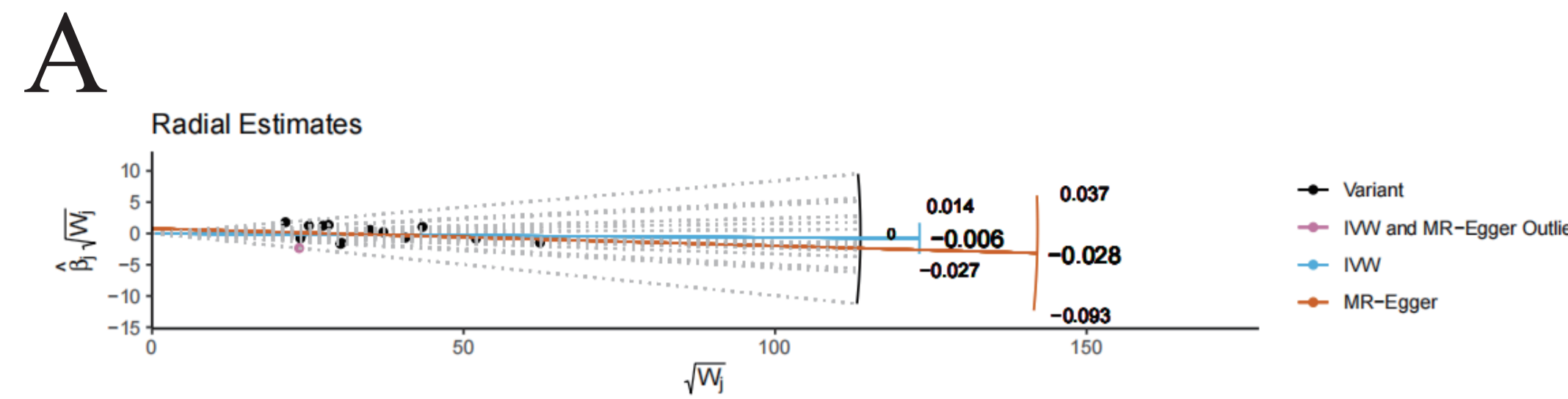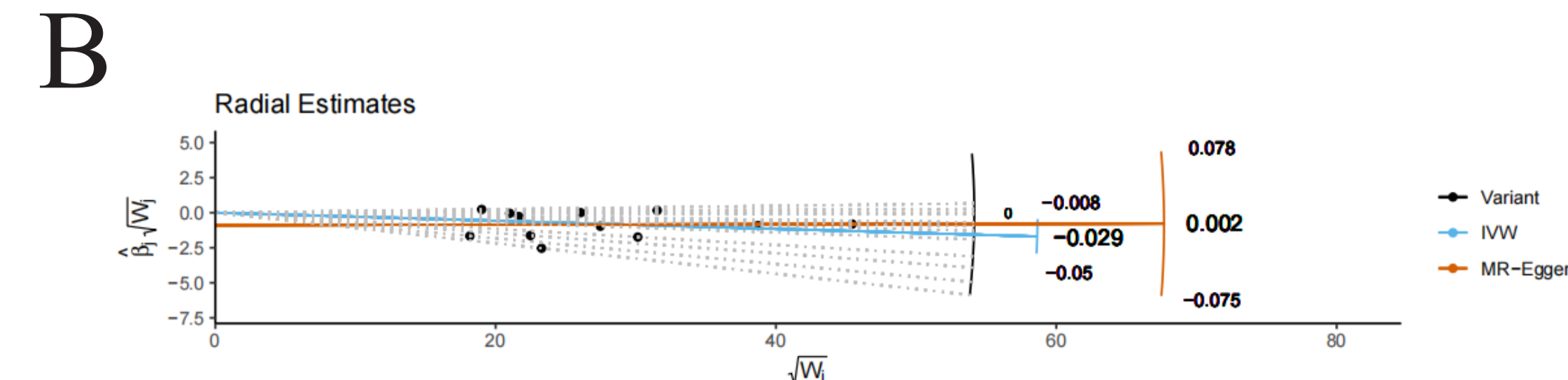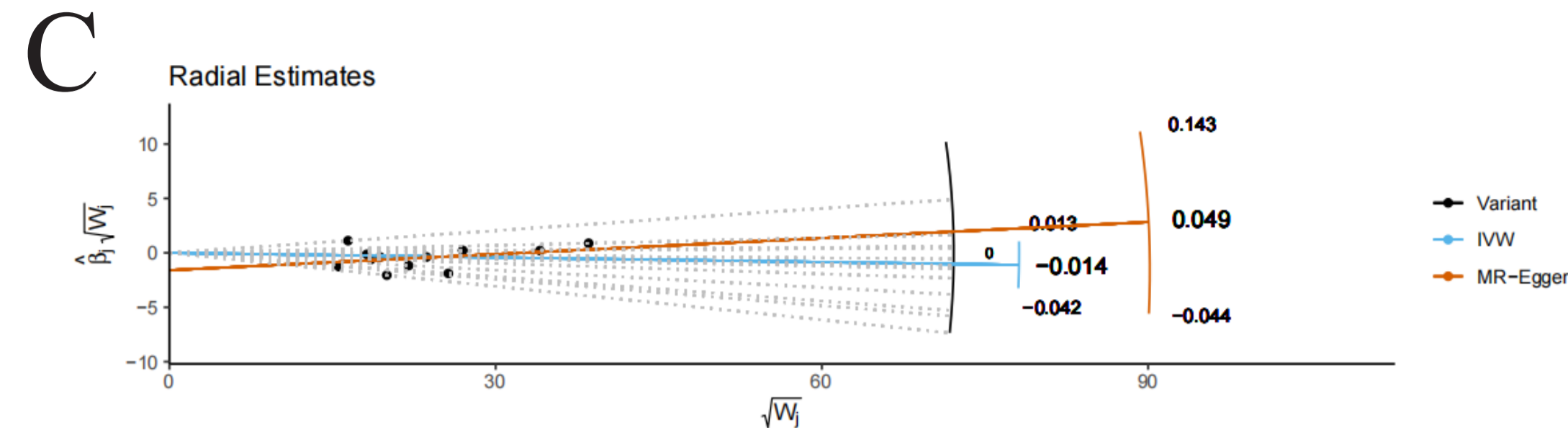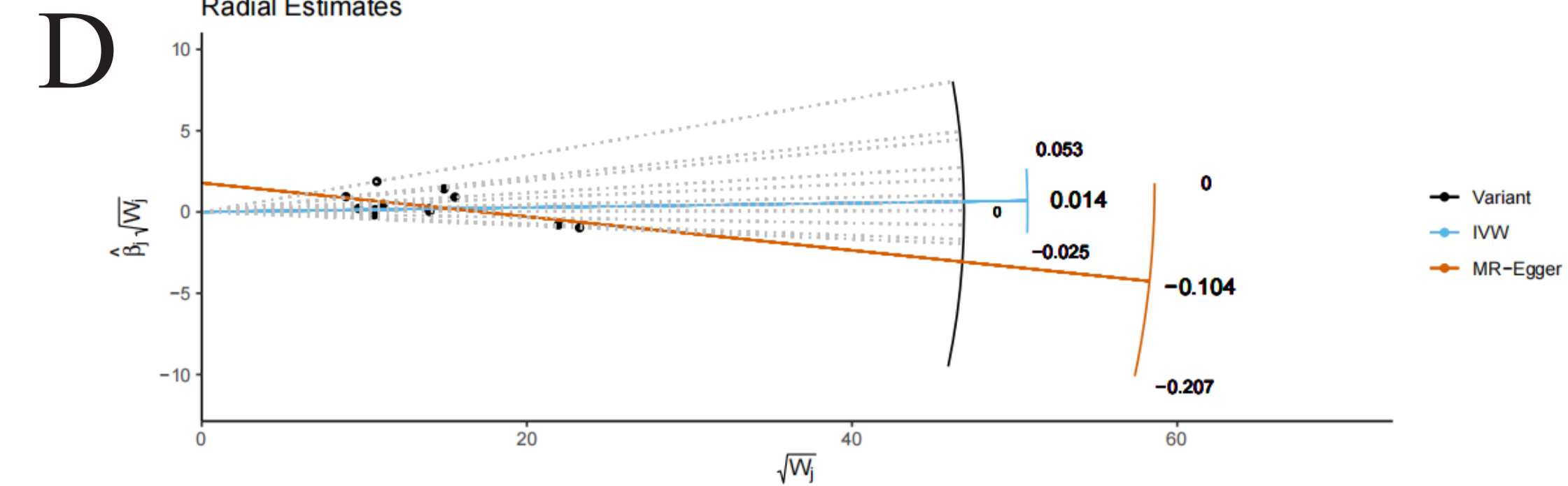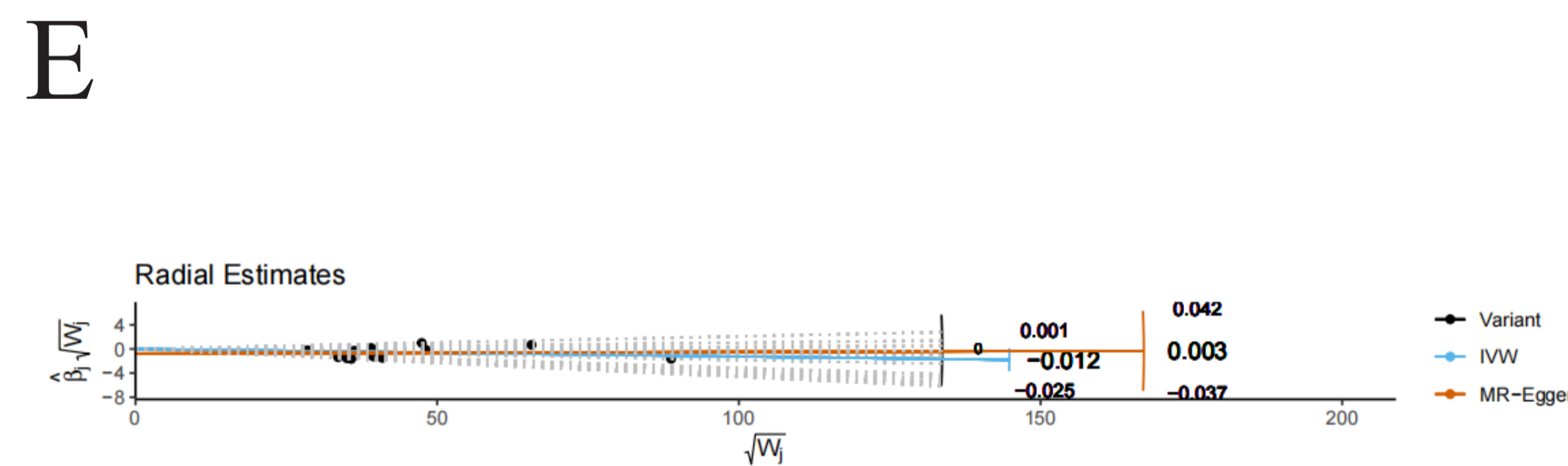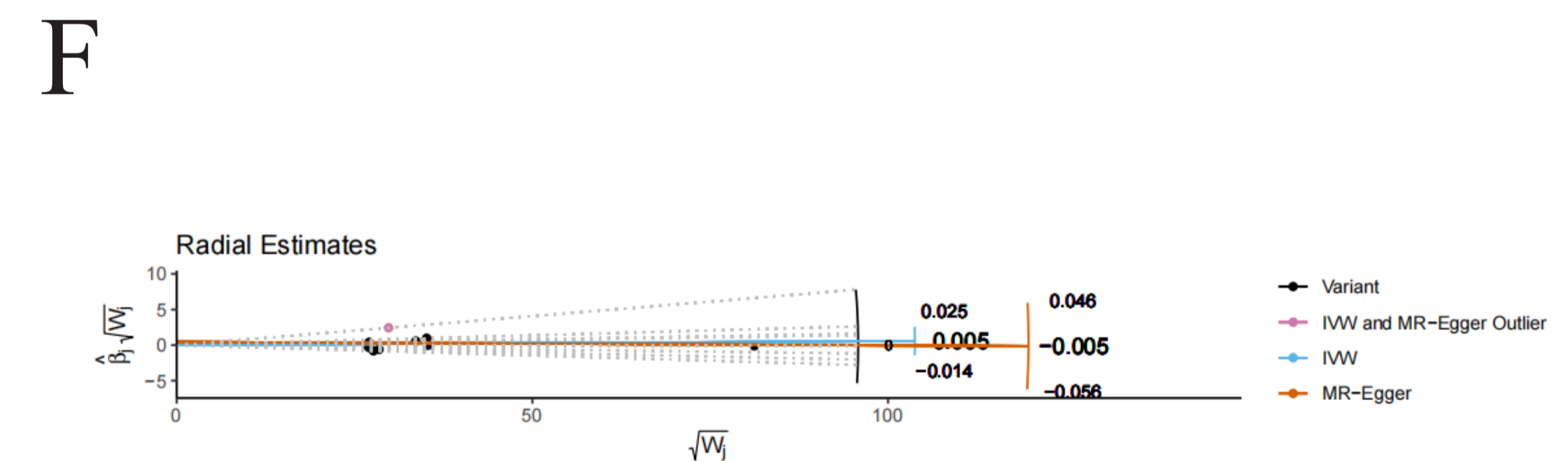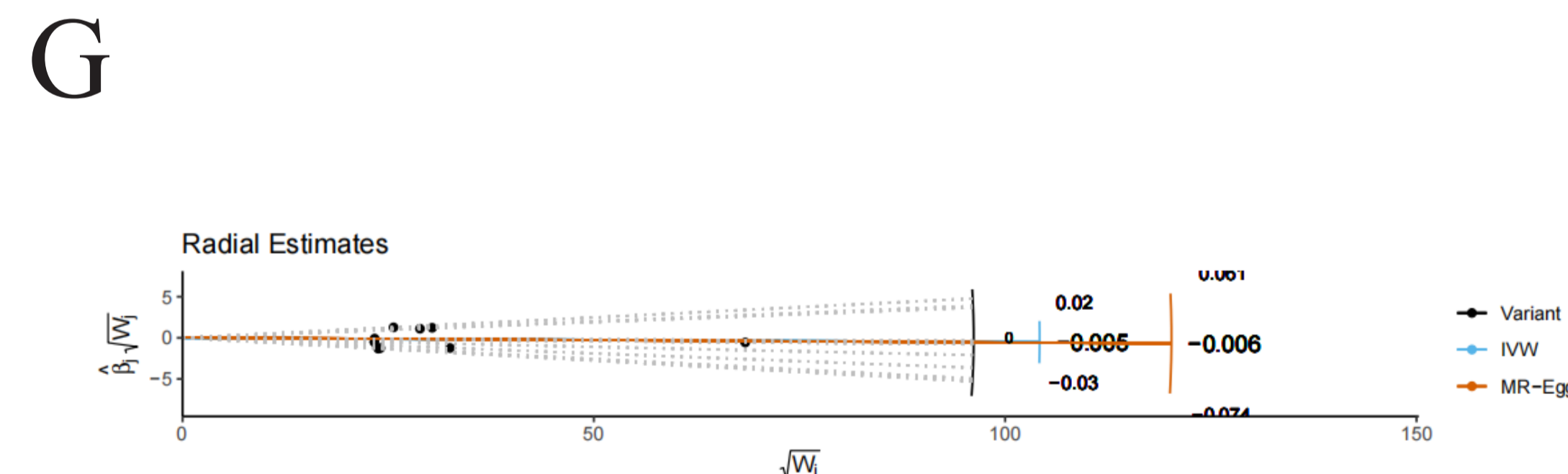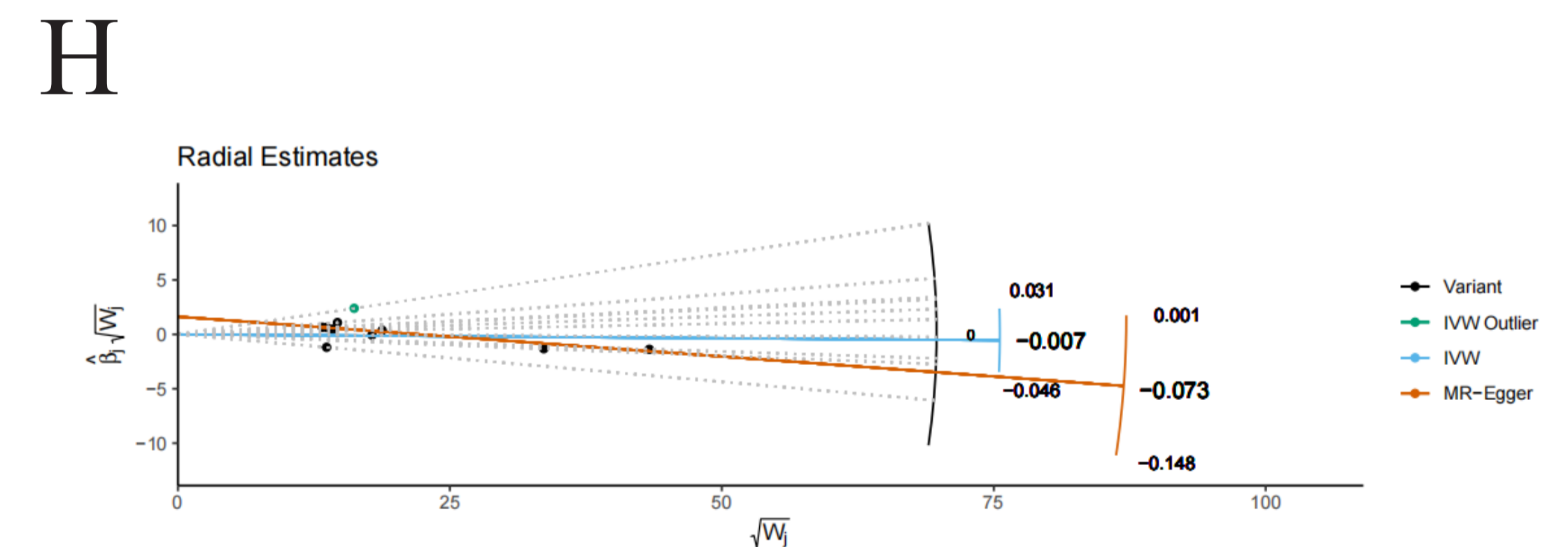

Supplement: S1 Fig — ALD (exposure) with A) TB-BMD(outcome); B) FN-BMD(outcome); C)LS-BMD(outcome); D) FA-BMD(outcome); NAFLD (exposure) with E) TB-BMD(outcome); F) FN-BMD(outcome); G)LS-BMD(outcome); H) FA-BMD(outcome). (PDF) [file pone.0292881.s001.pdf]

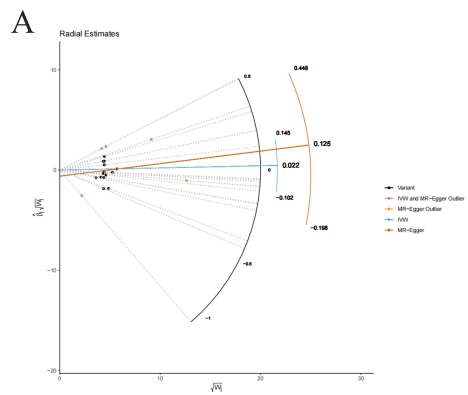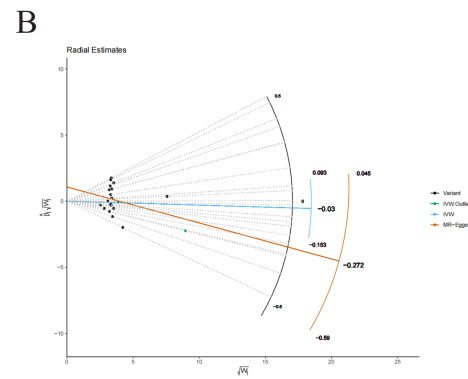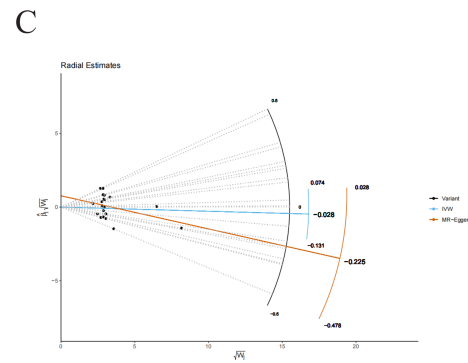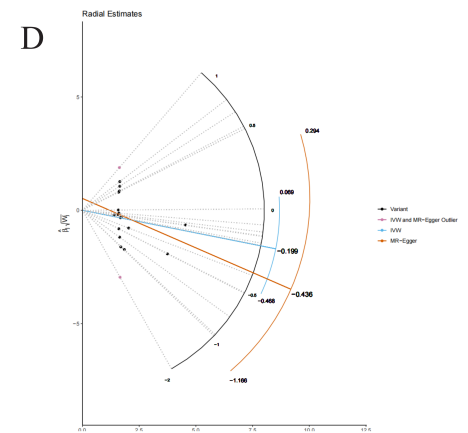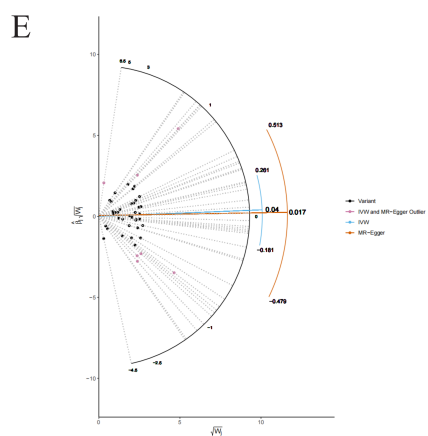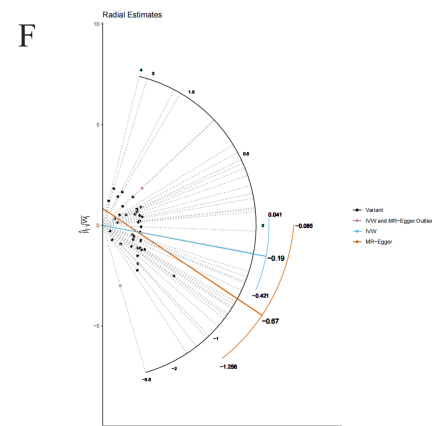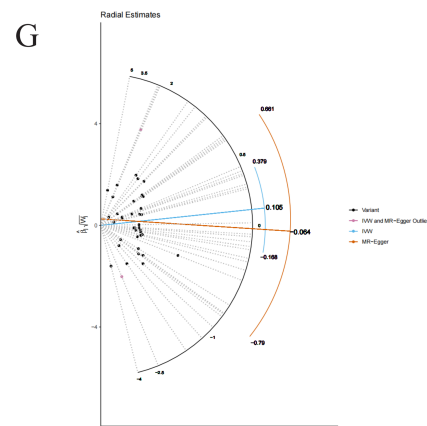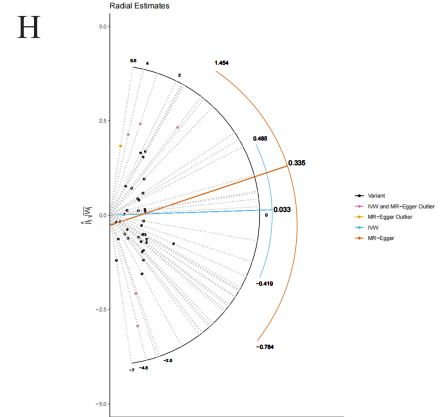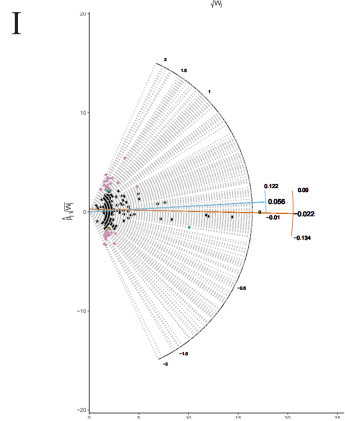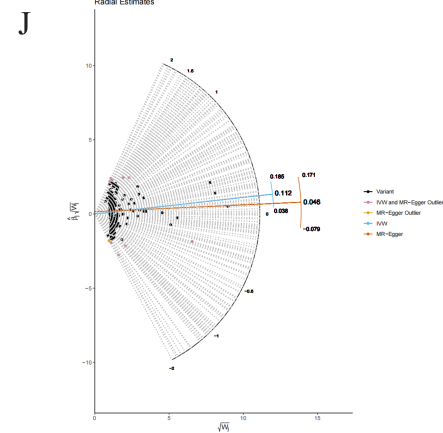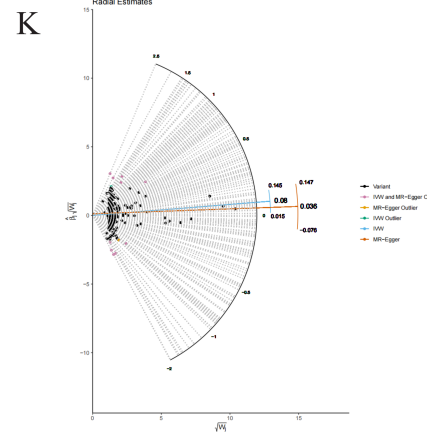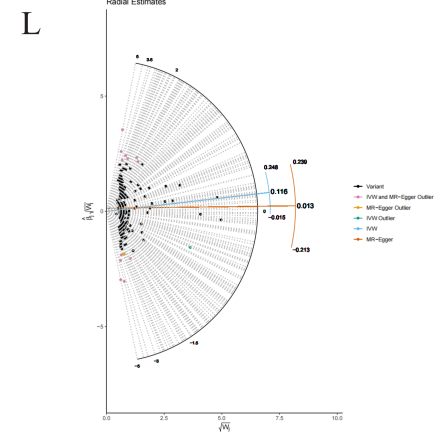

Supplement: S2 Fig — number of cigarettes per day(exposure) with A)TB-BMD(outcome); B) FN-BMD(outcome); C)LS-BMD(outcome); D) FA-BMD(outcome); alcohol consumption per week(exposure) with E) TB-BMD(outcome); F) FN-BMD(outcome); G)LS-BMD(outcome); H) FA-BMD(outcome); Serum 25-Hydroxyvitamin D levels(exposure) with I) TB-BMD(outcome); J) FN-BMD(outcome); K)LS-BMD(outcome); L) FA-BMD(outcome). (PDF) [file pone.0292881.s002.pdf]

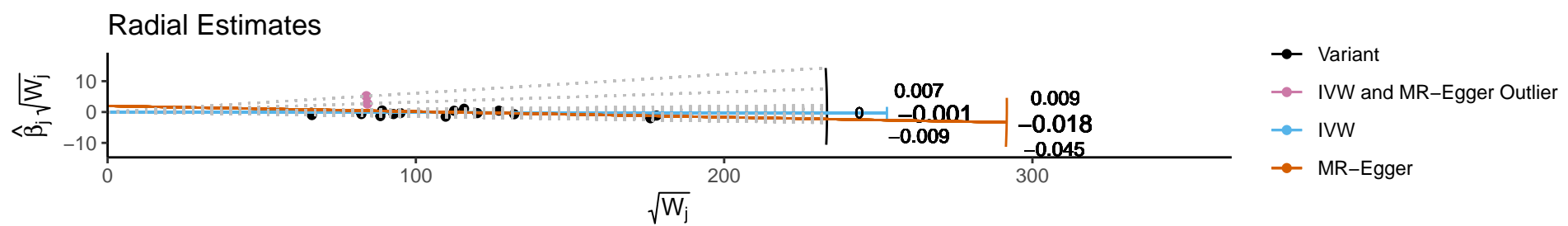

Supplement: S3 Fig — ALD (exposure) with Serum 25-Hydroxyvitamin D levels (outcome). (PDF) [file pone.0292881.s003.pdf]

A

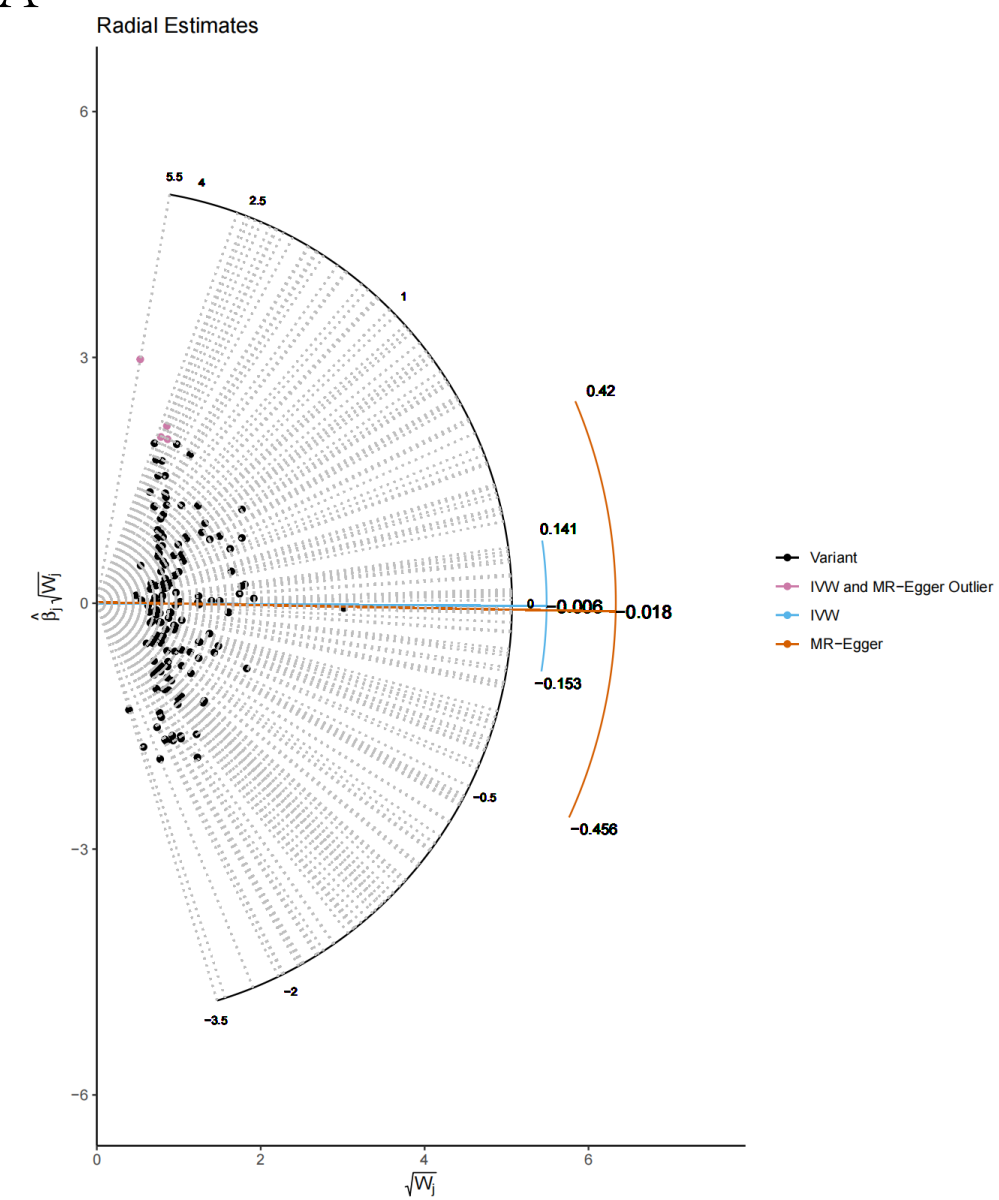

B

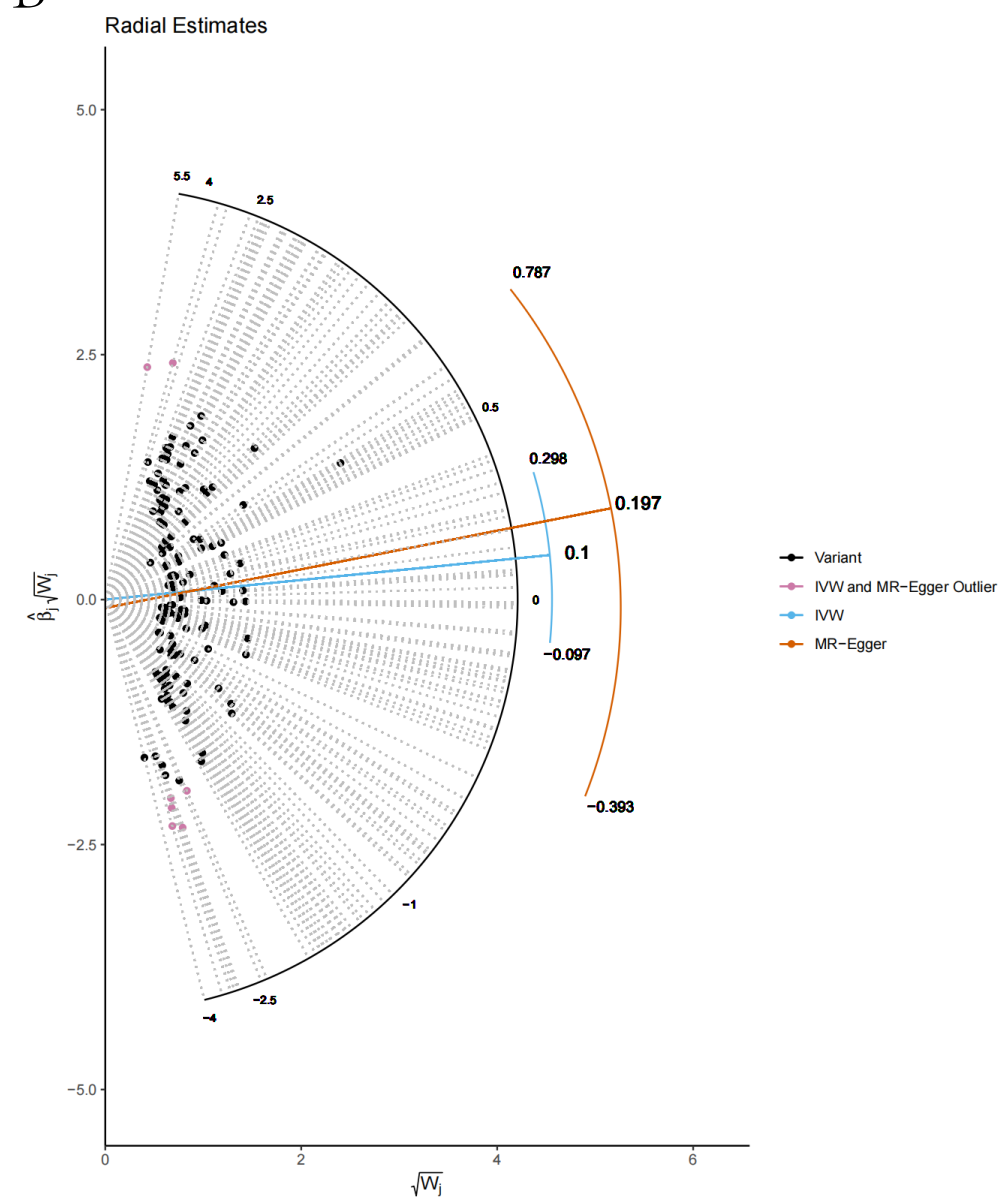

C

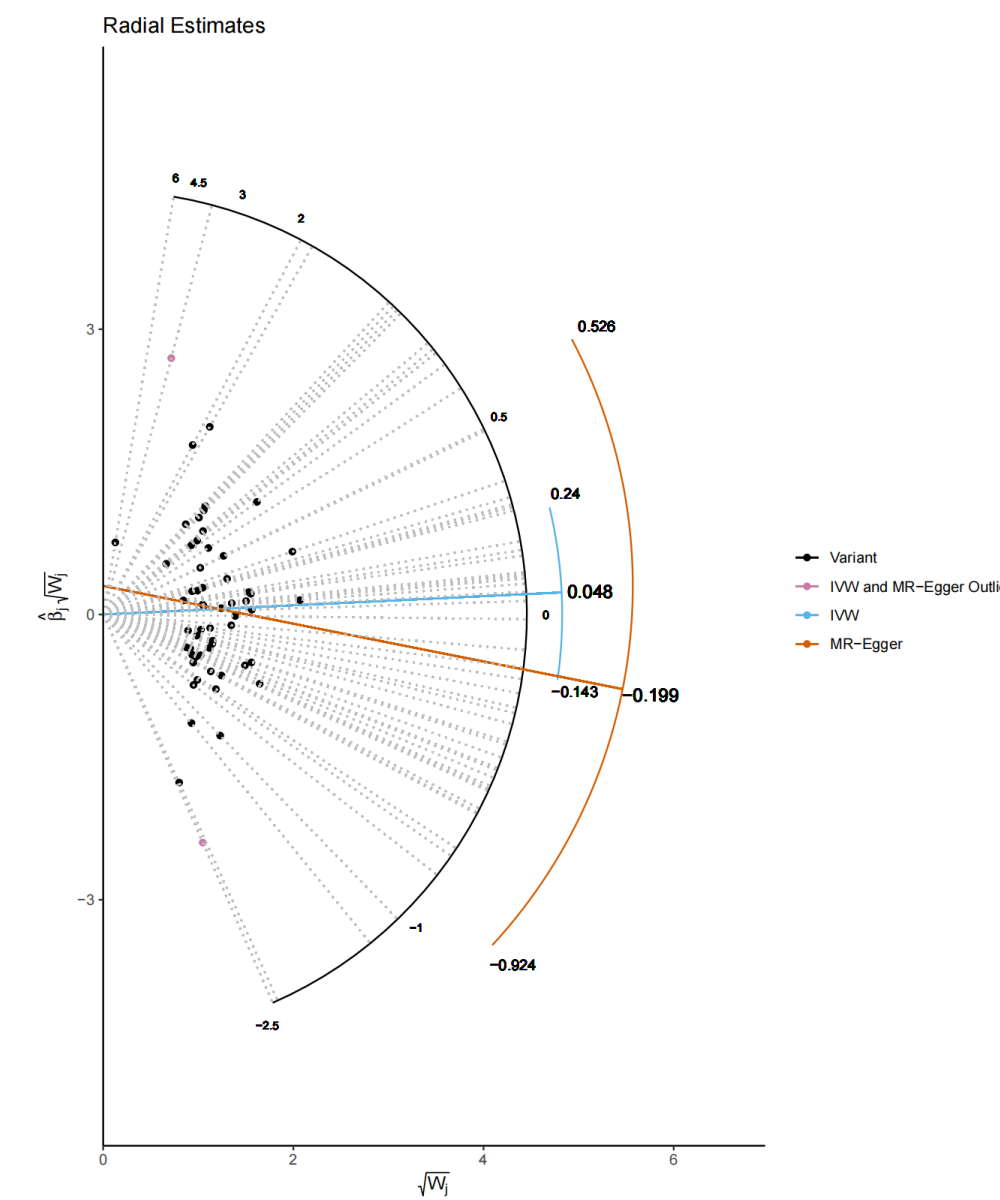

D

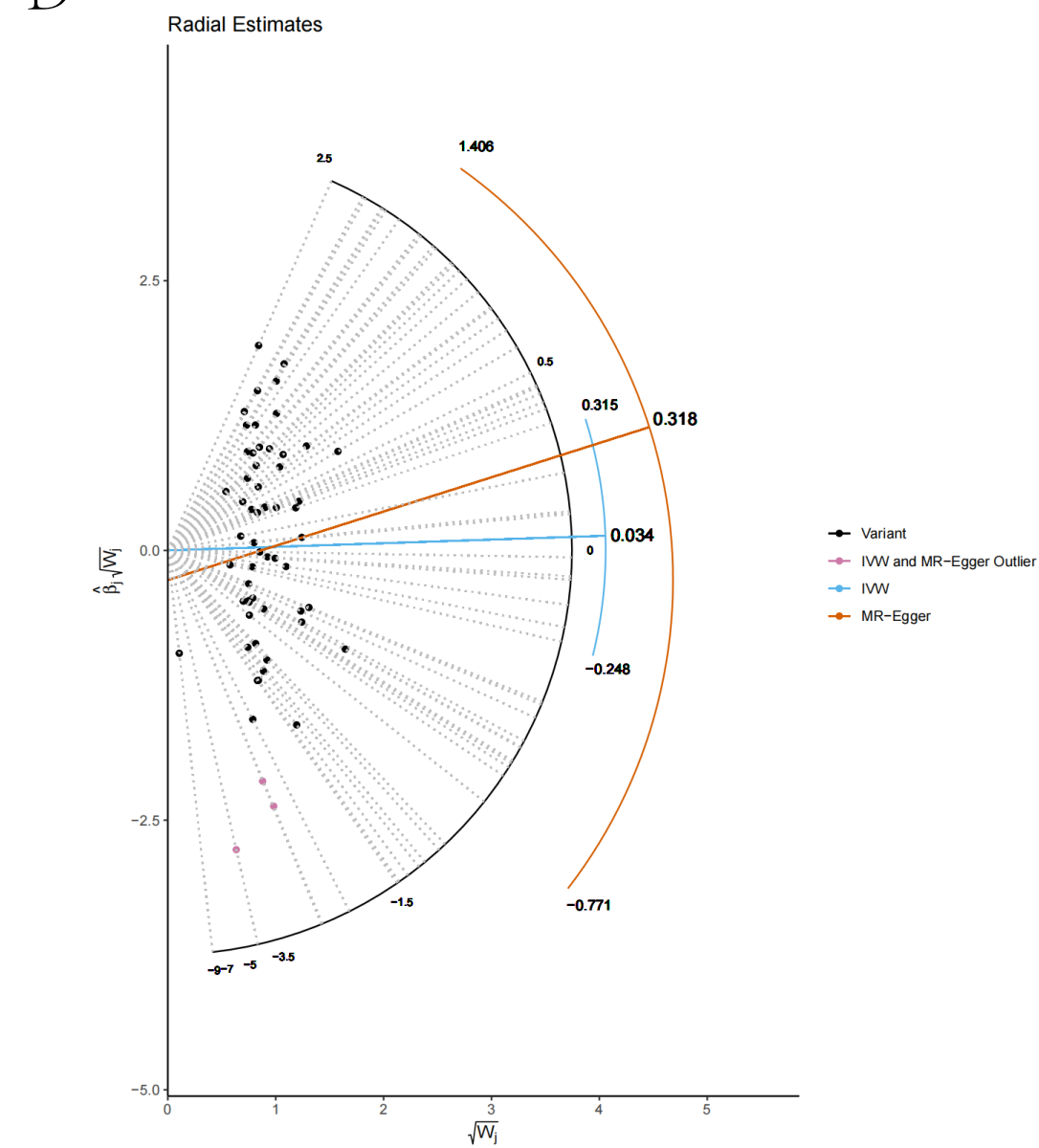

E

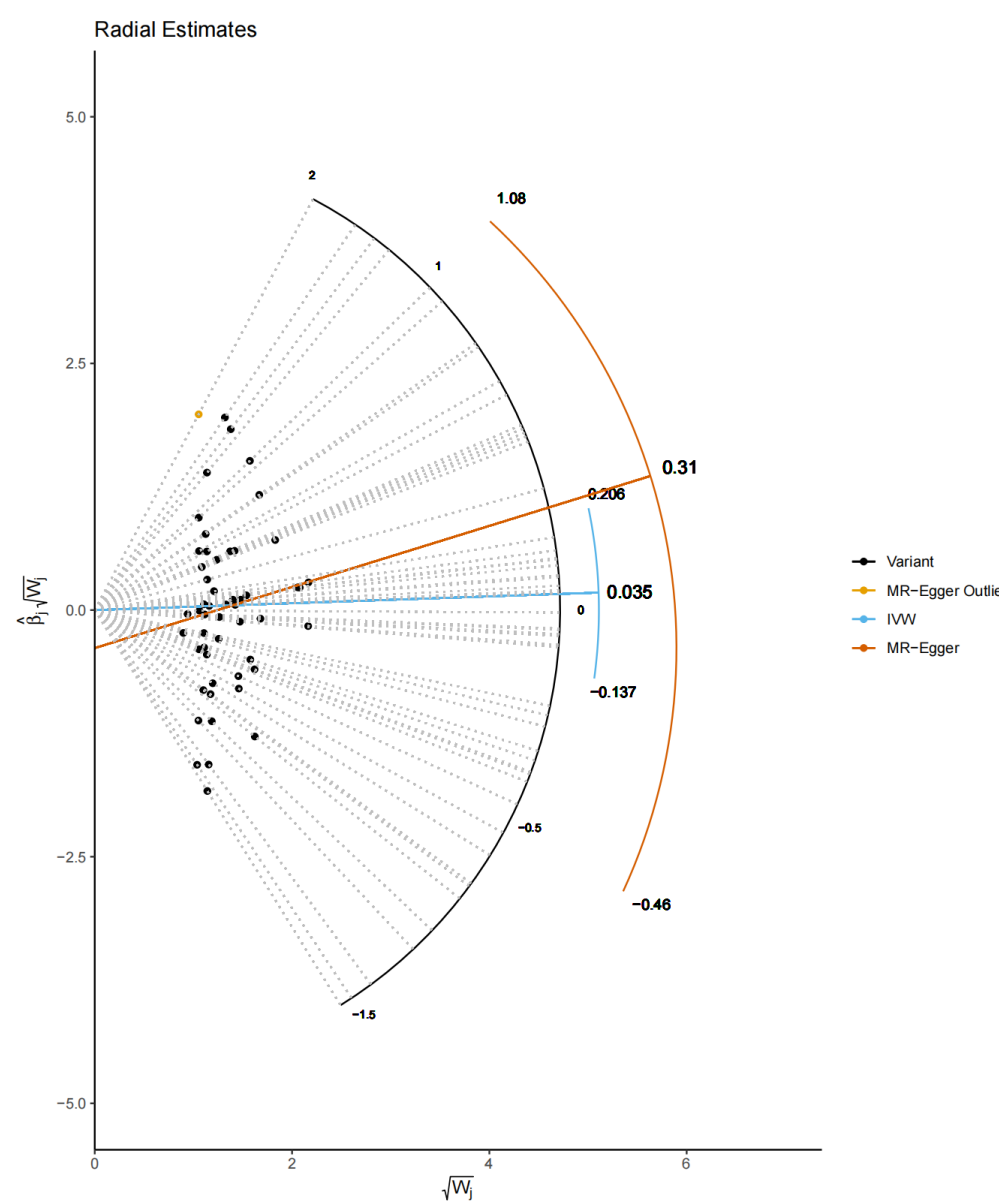

F

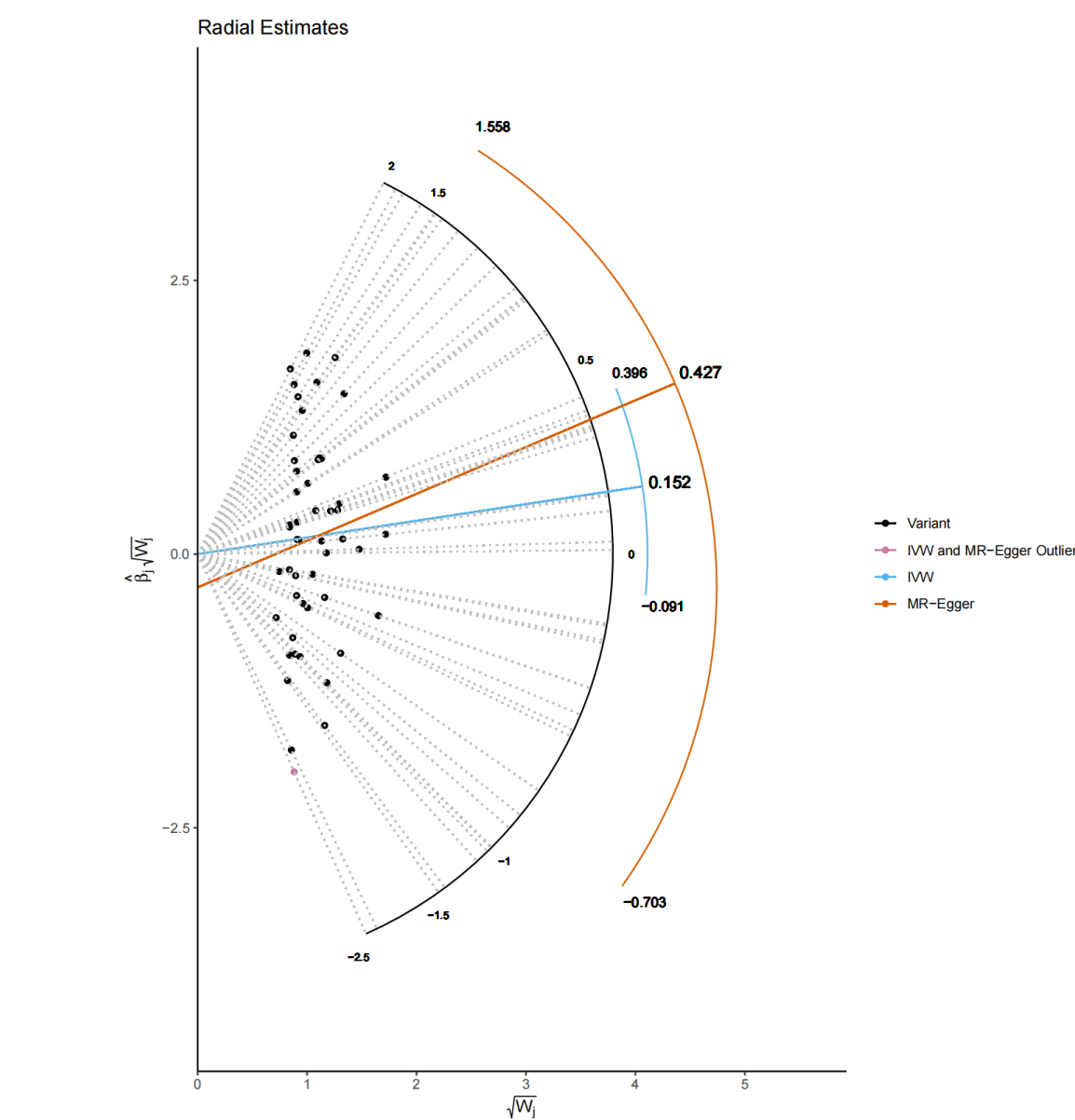

G

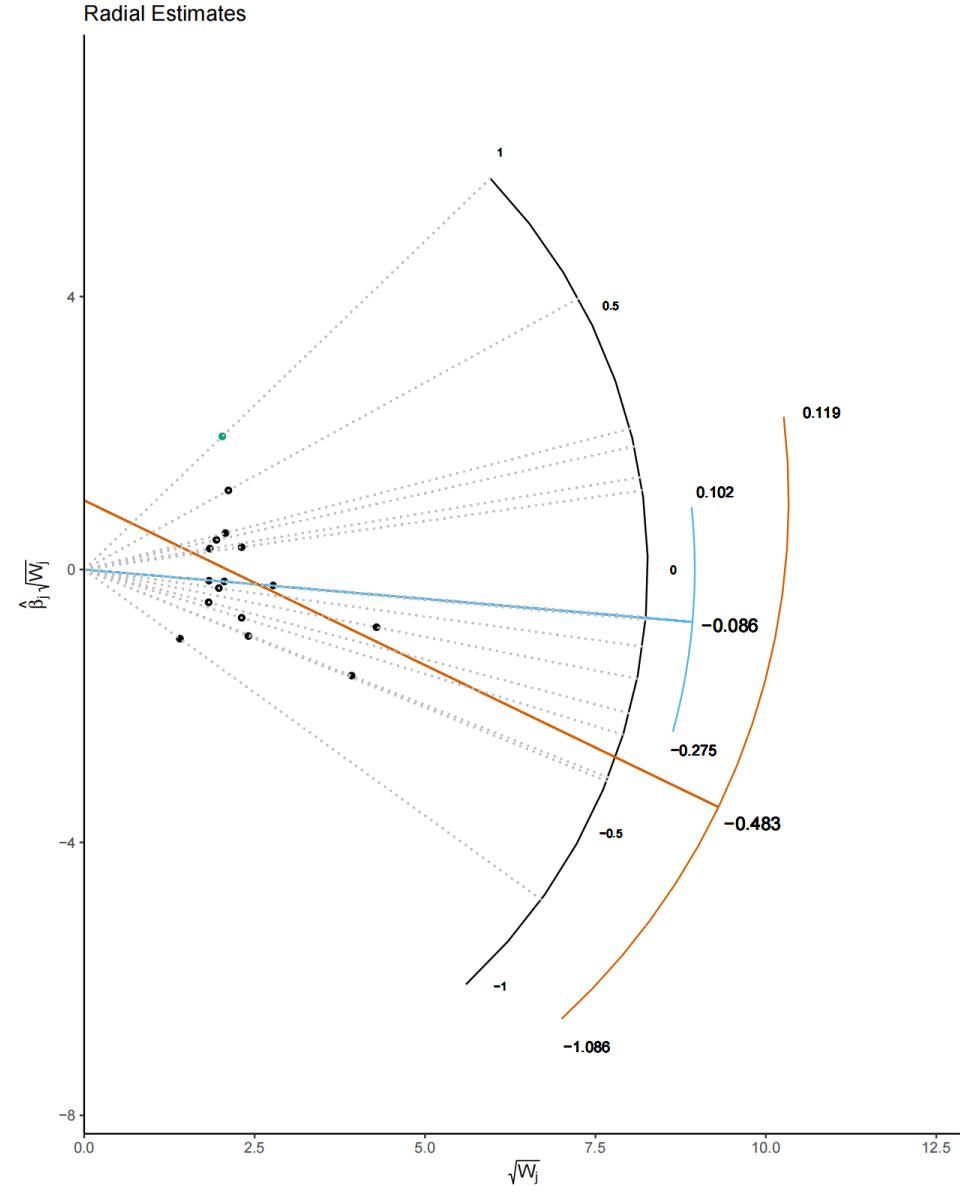

H

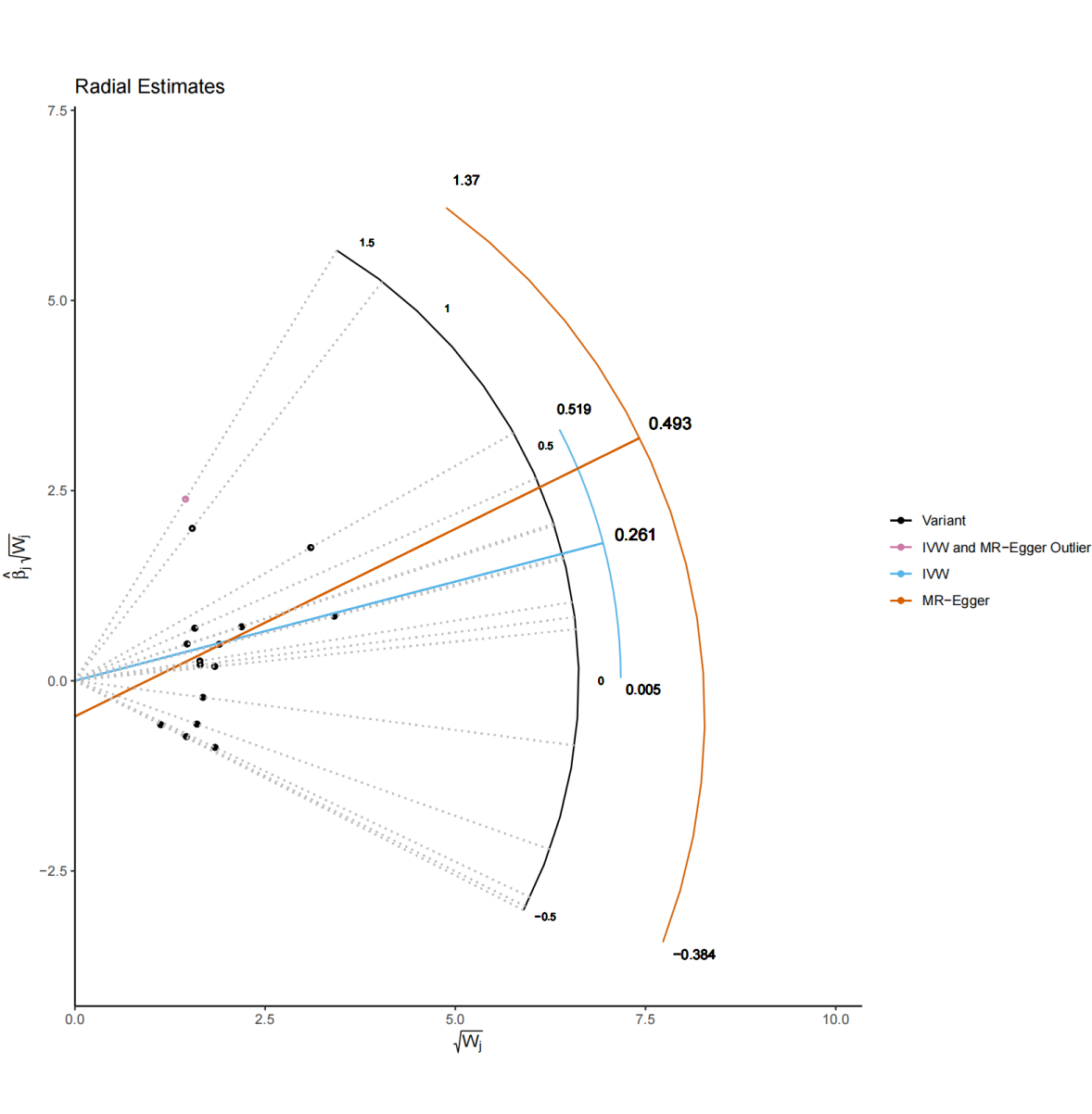

Supplement: S4 Fig — TB-BMD(exposure) with A) ALD(outcome); B)NAFL D(outcome); FN-BMD(exposure) with C) ALD(outcome);D)NAFLD(outcome);LS-BMD(exposure)with E)ALD(outcome); F)NAFLD(outcome);FA-BMD(exposure) with G) ALD(outcome); H)NAFLD(outcome). (PDF) [file pone.0292881.s004.pdf]

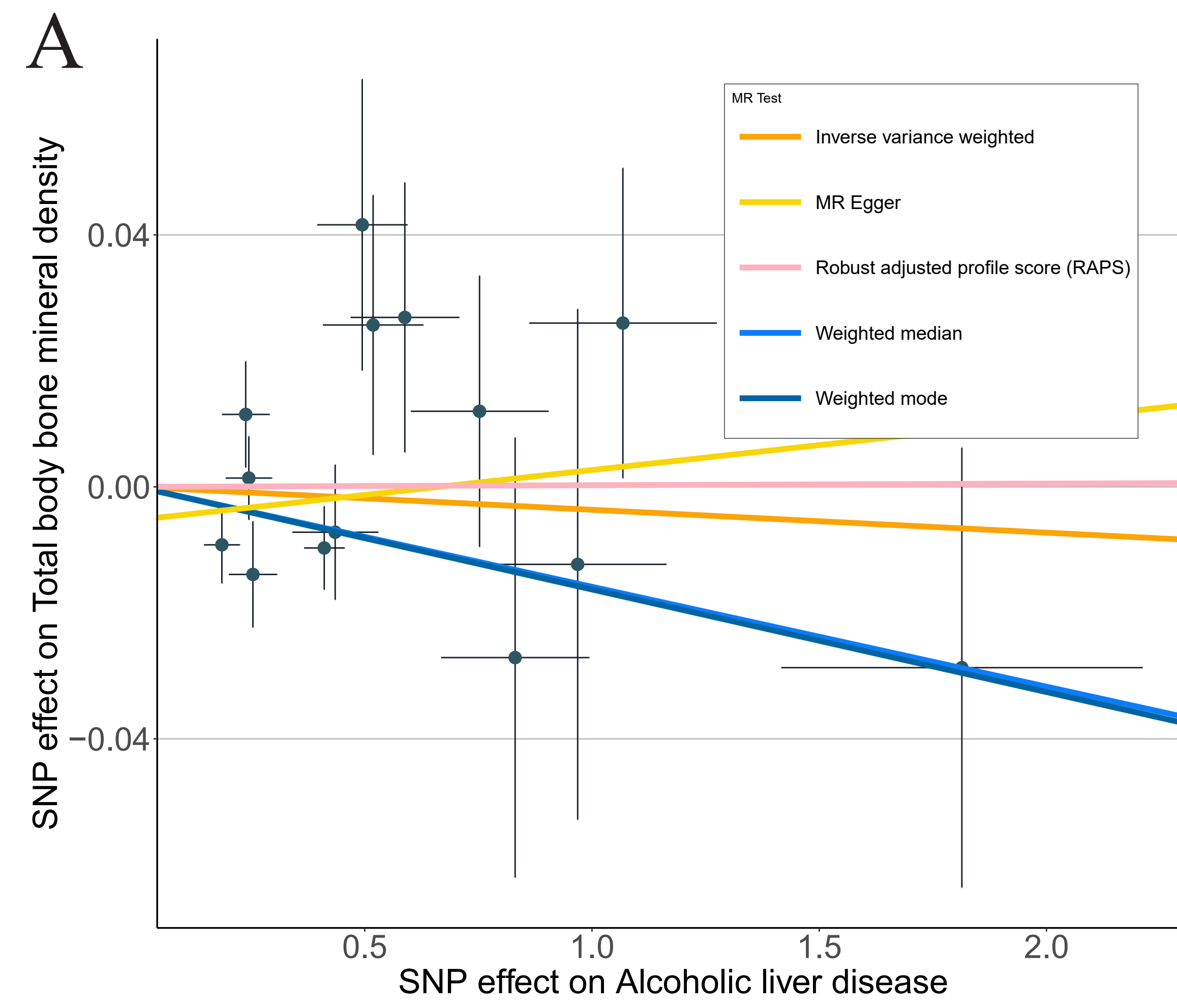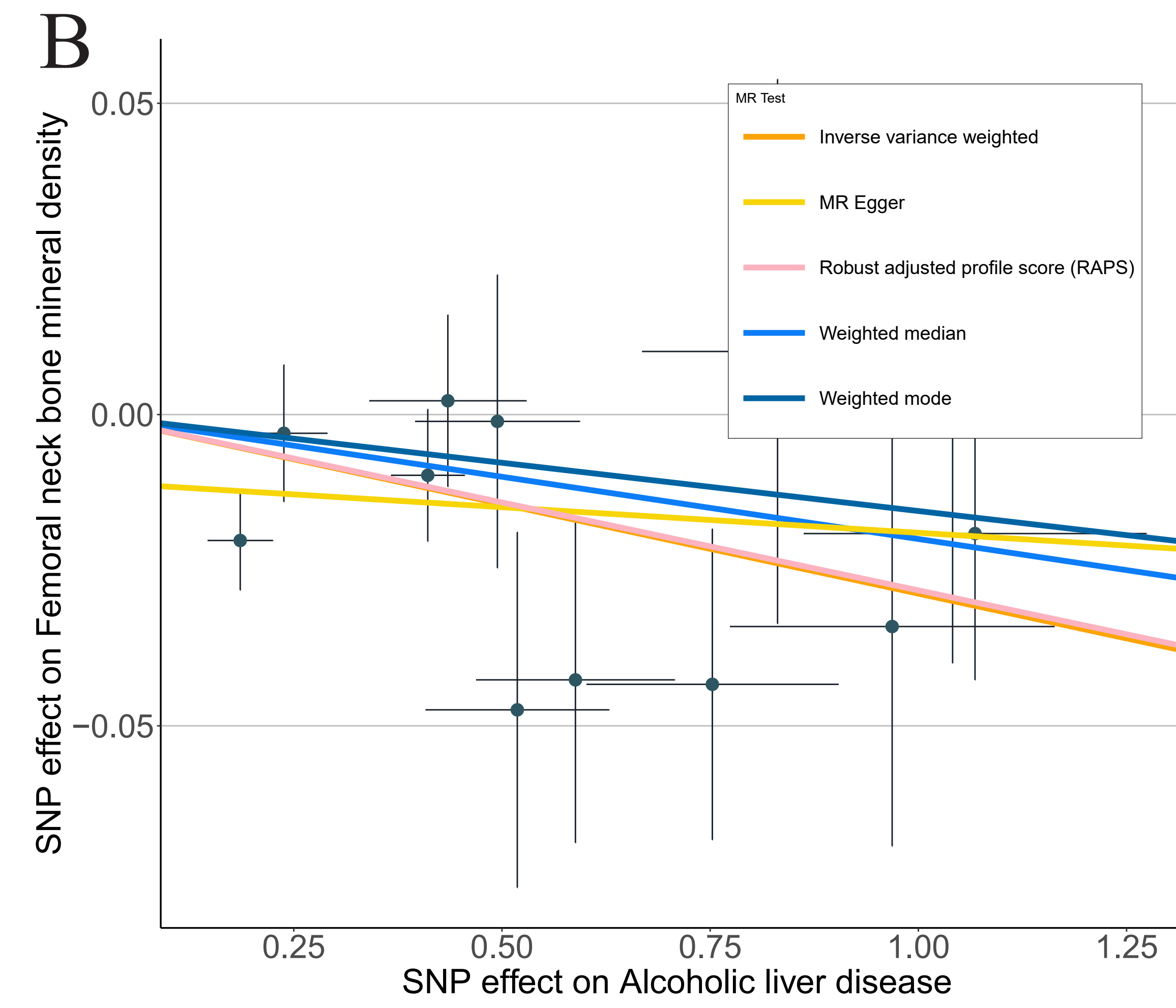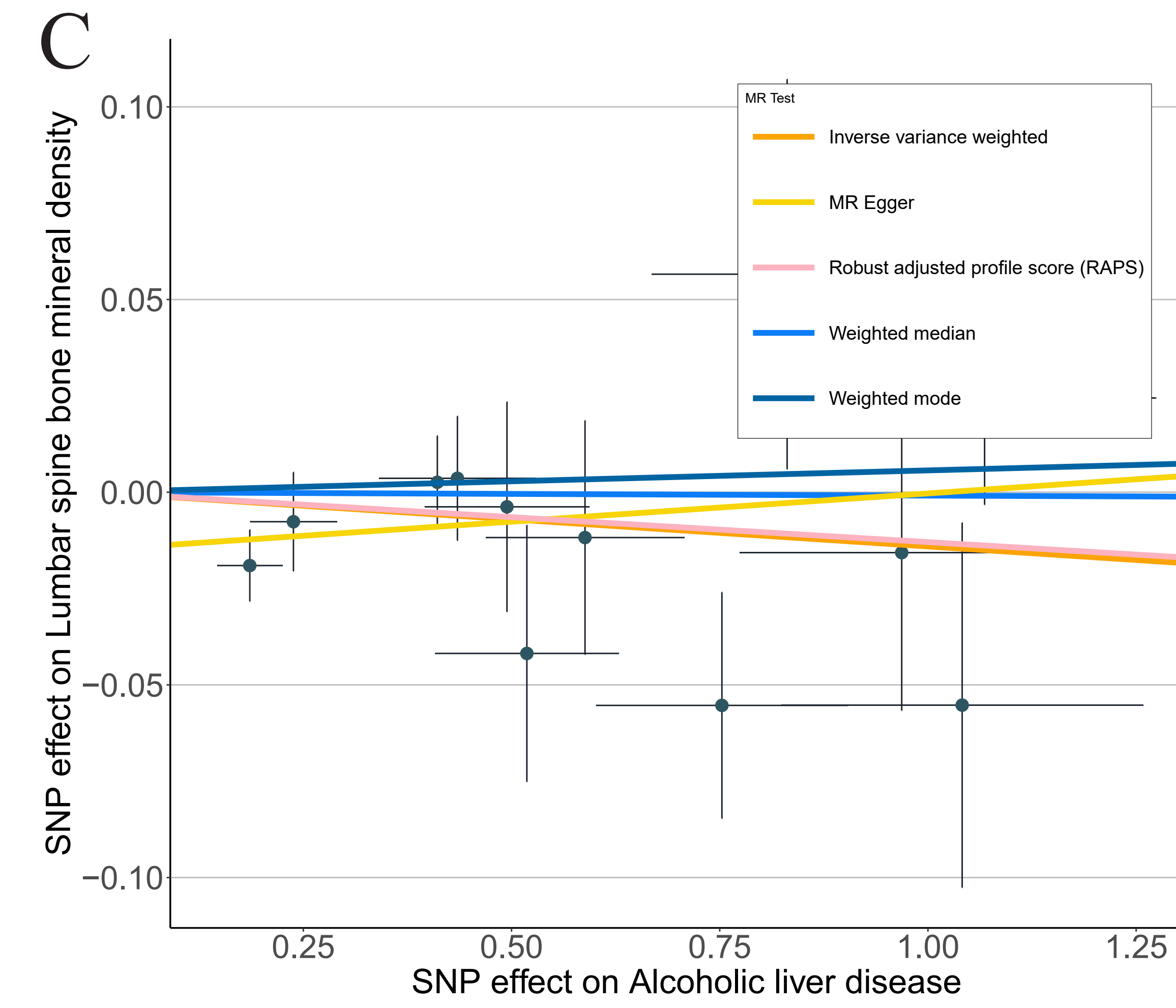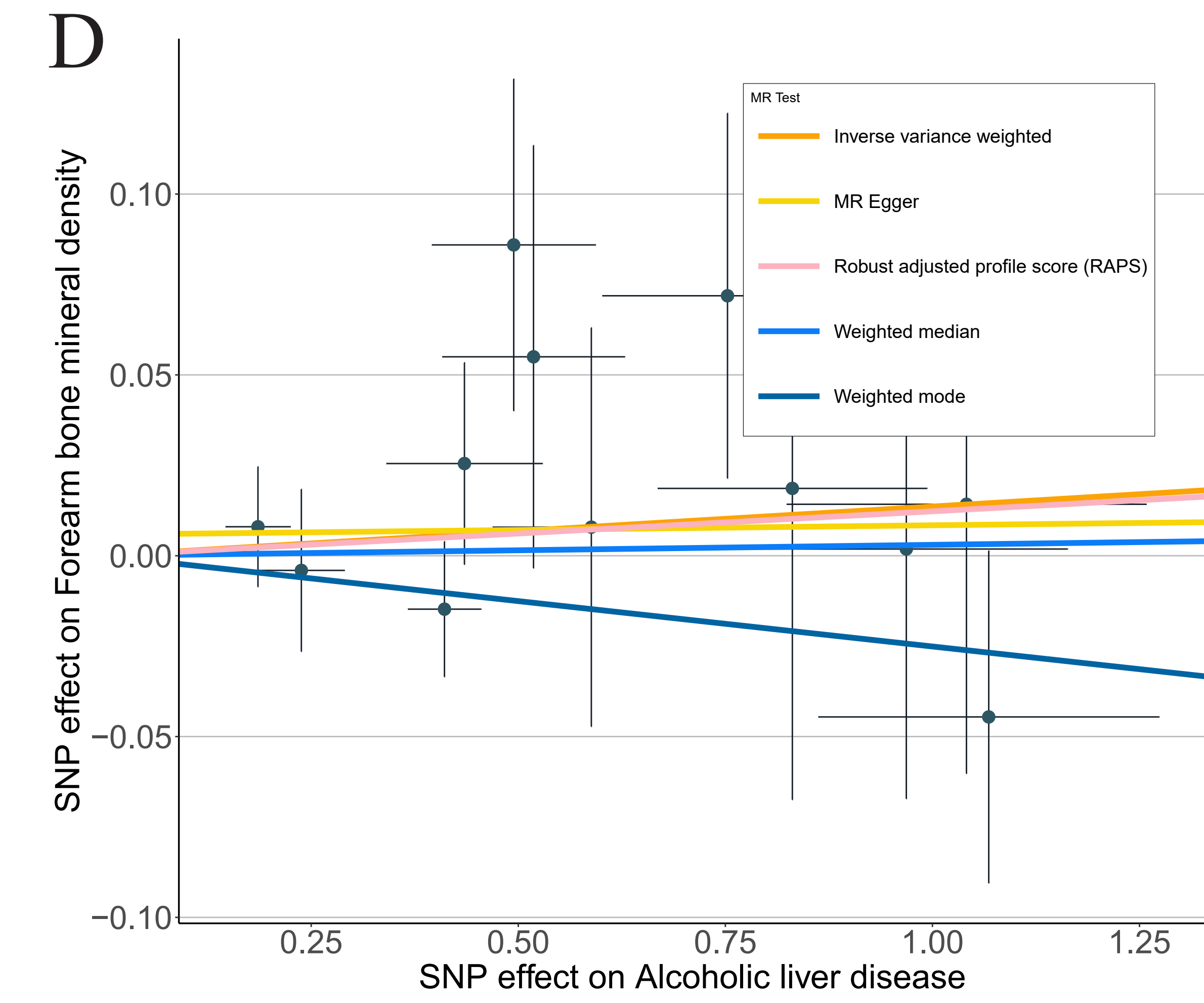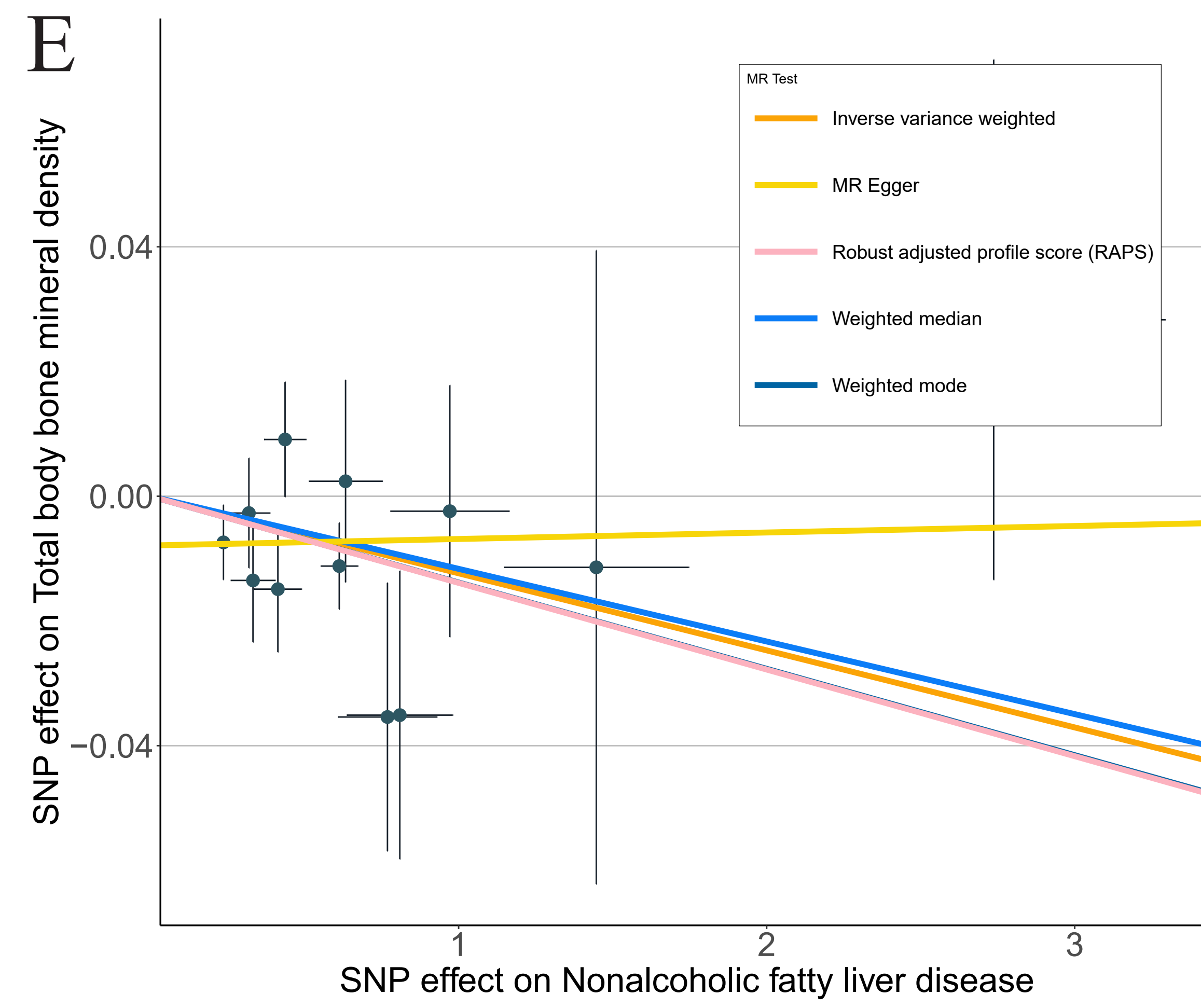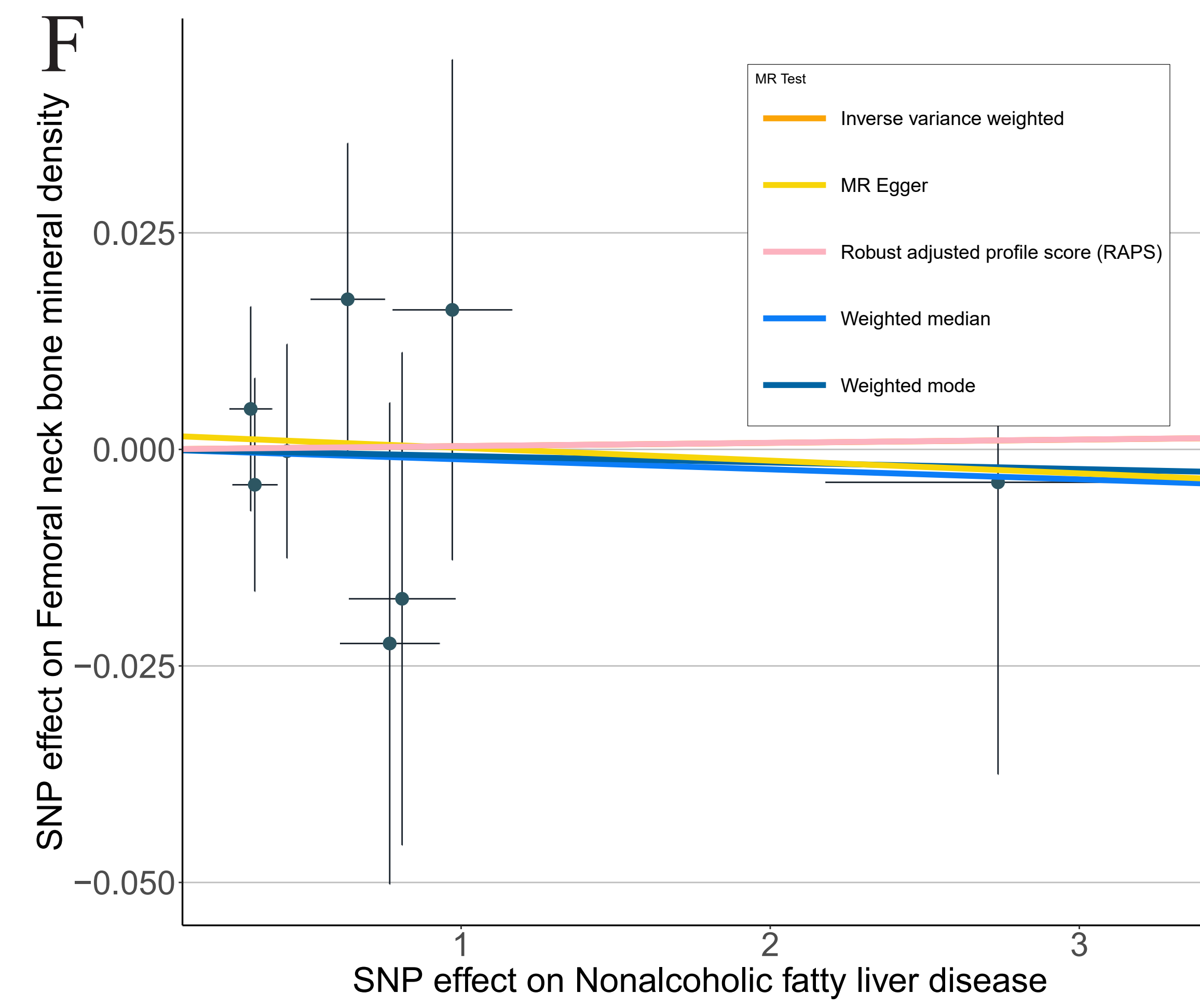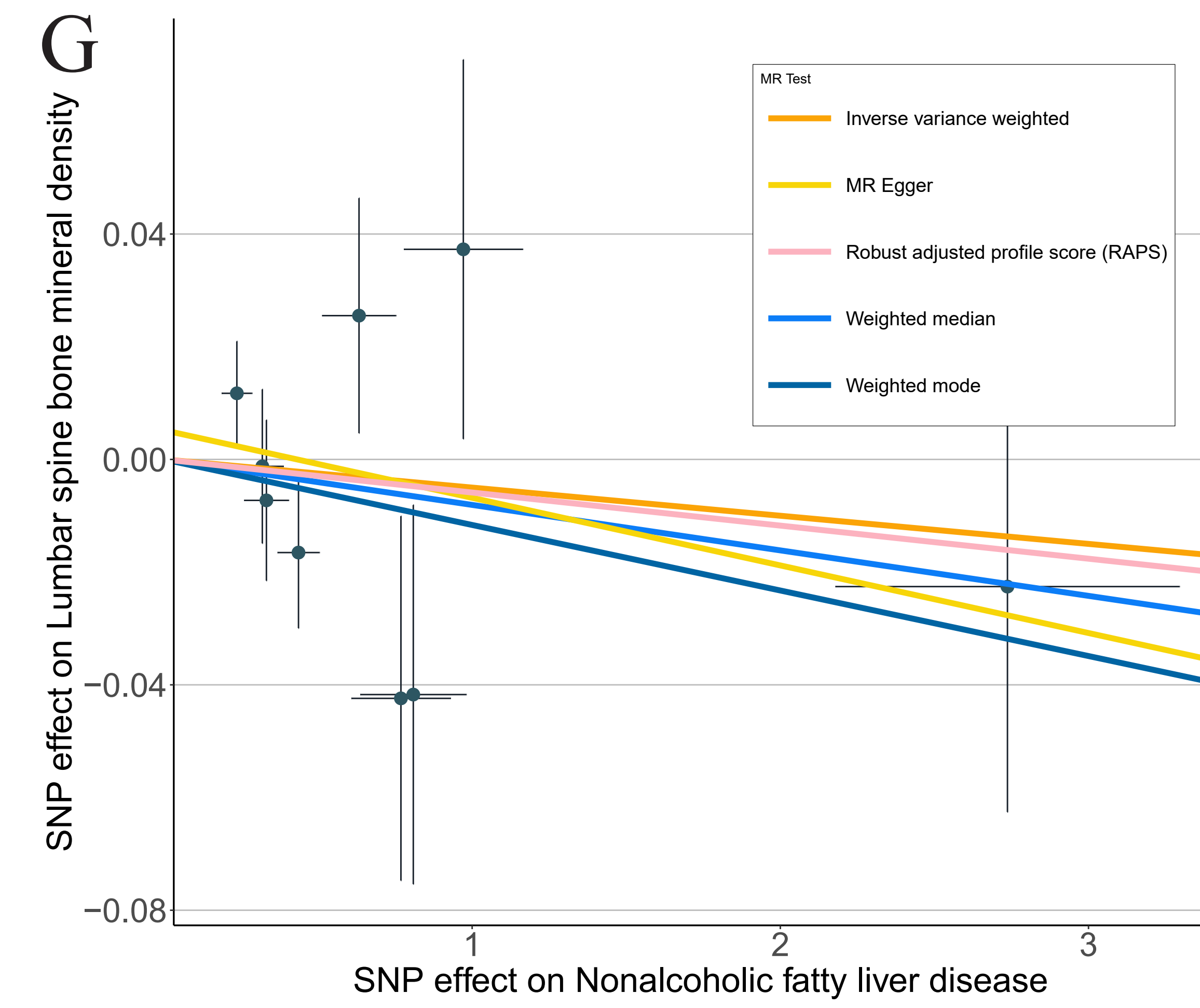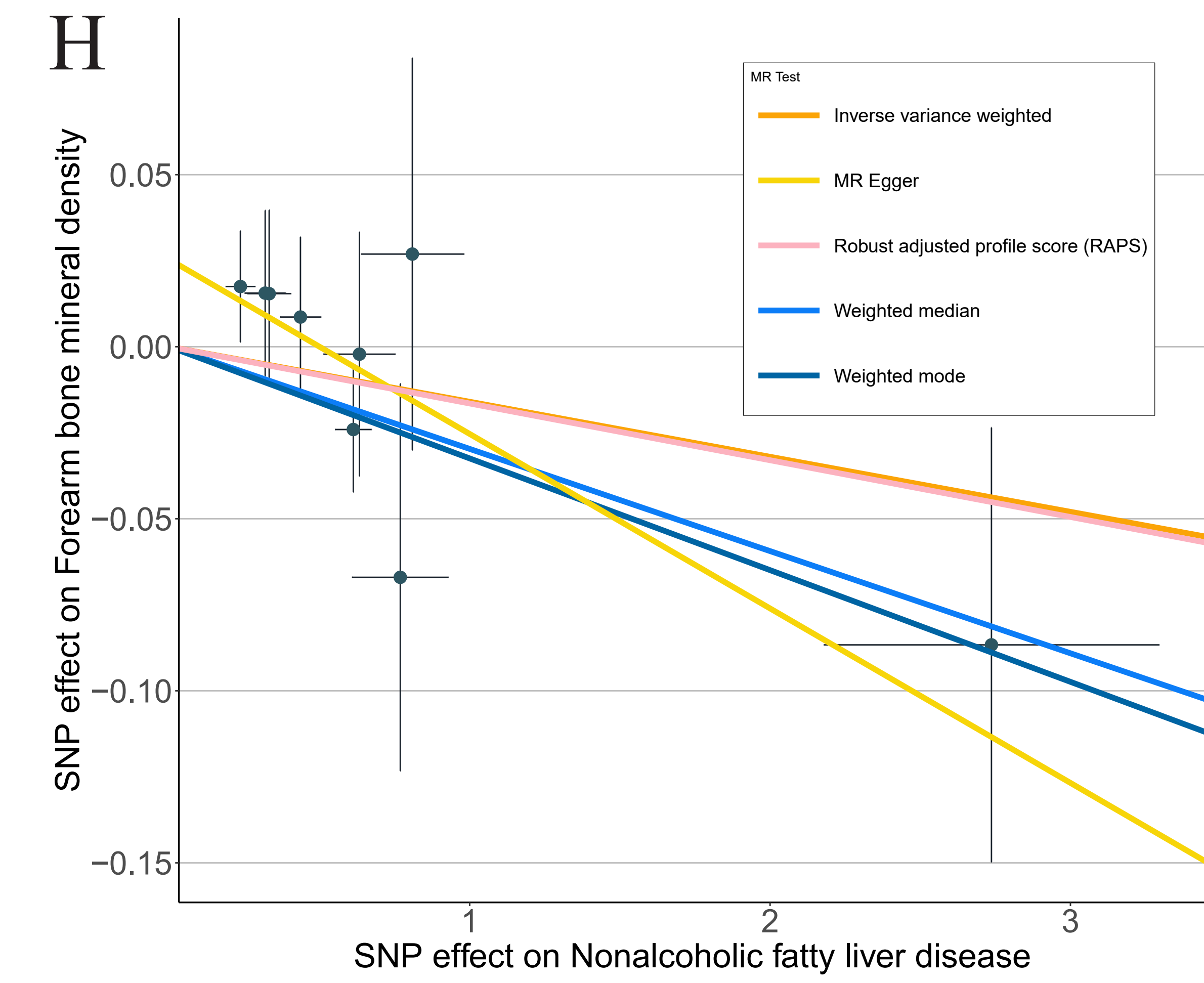

Supplement: S5 Fig — Scatterplot of associations of genetically predicted ALD on the risk of A)TB-BMD; B)FN-BMD;C) LS-BMD; D)FA-BMD; Scatterplot of associations of genetically predicted NAFLD on the risk of E)TB-BMD; F)FN-BMD;G) LS-BMD;H)FA-BMD. (PDF) [file pone.0292881.s005.pdf]

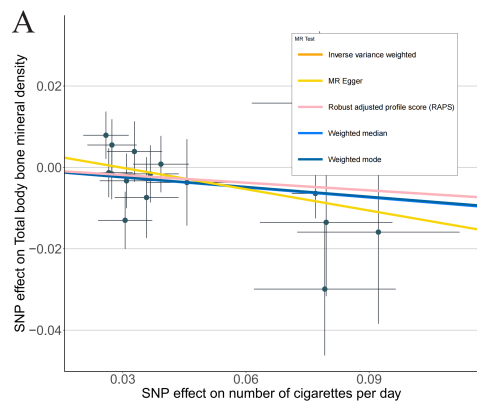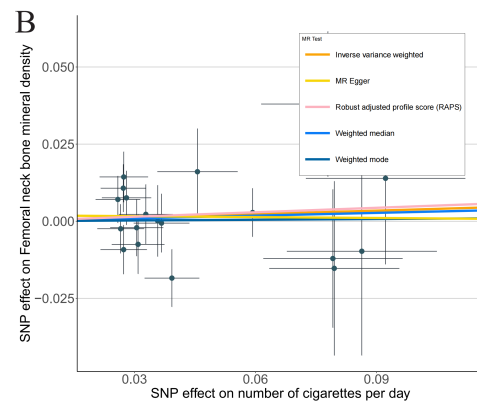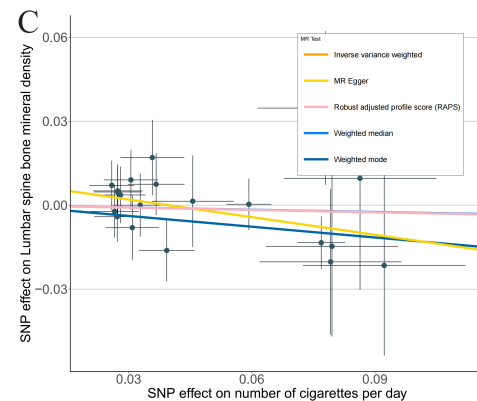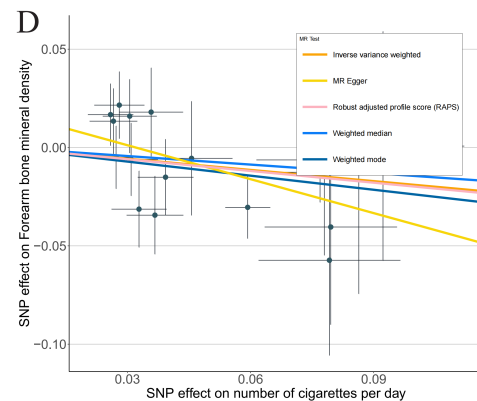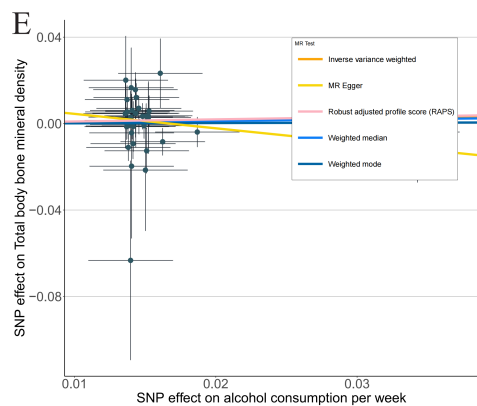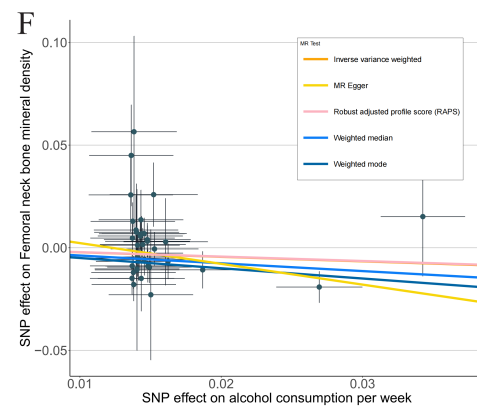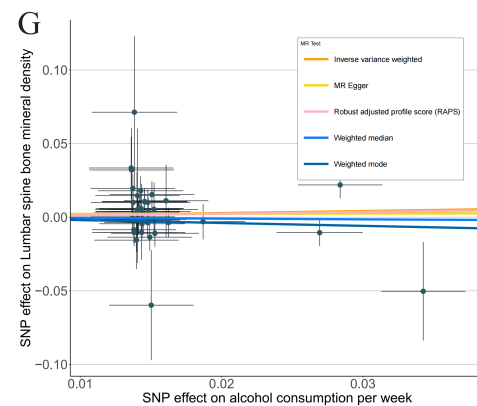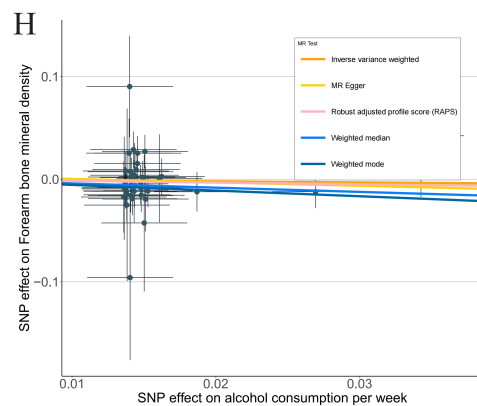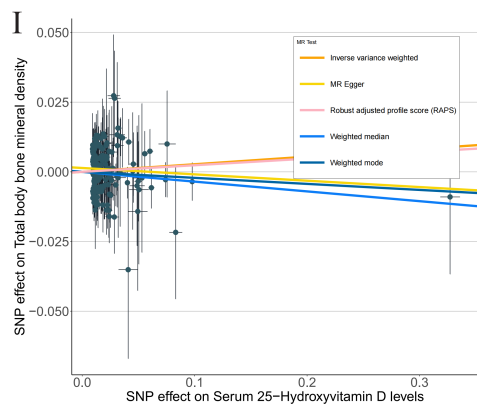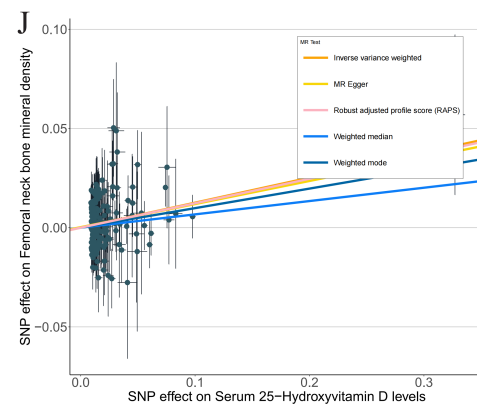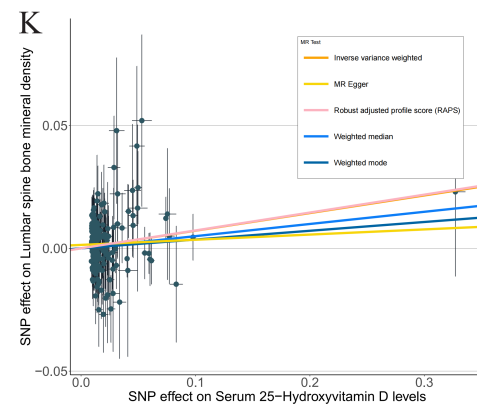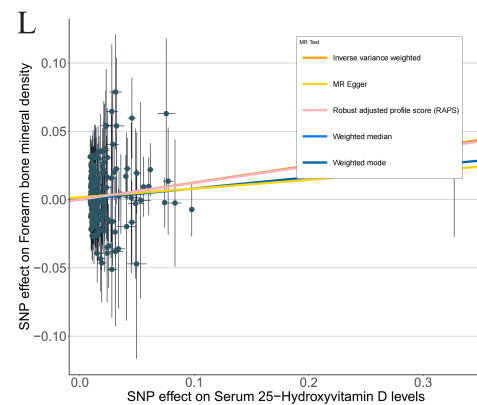

Supplement: S6 Fig — Scatterplot of associations of genetically predicted number of cigarettes per day on the risk of A)TB-BMD; B)FN-BMD;C) LS-BMD; D)FA-BMD; Scatterplot of associations of genetically predicted of alcohol consumption per week on the risk of E)TB-BMD; F)FN-BMD;G) LS-BMD; H)FA-BMD; Scatterplot of associations of genetically predicted of serum 25-Hydroxyvitamin D levels on the risk of I)TB-BMD; J)FN-BMD;K) LS-BMD; L)FA-BMD. (PDF) [file pone.0292881.s006.pdf]

SNP effect on Serum 25-Hydroxyvitamin D levels

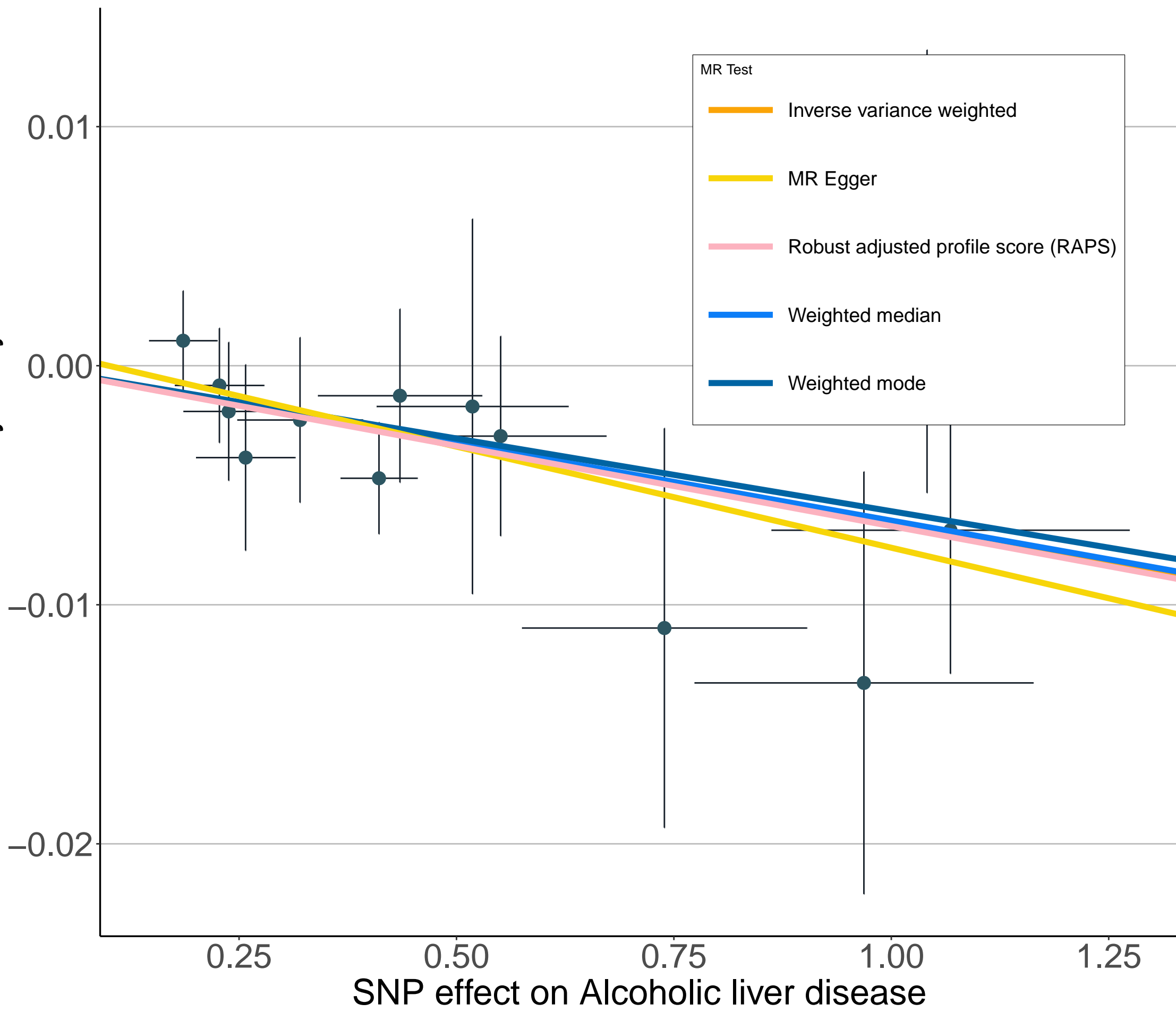

Supplement: S7 Fig — (PDF) [file pone.0292881.s007.pdf]

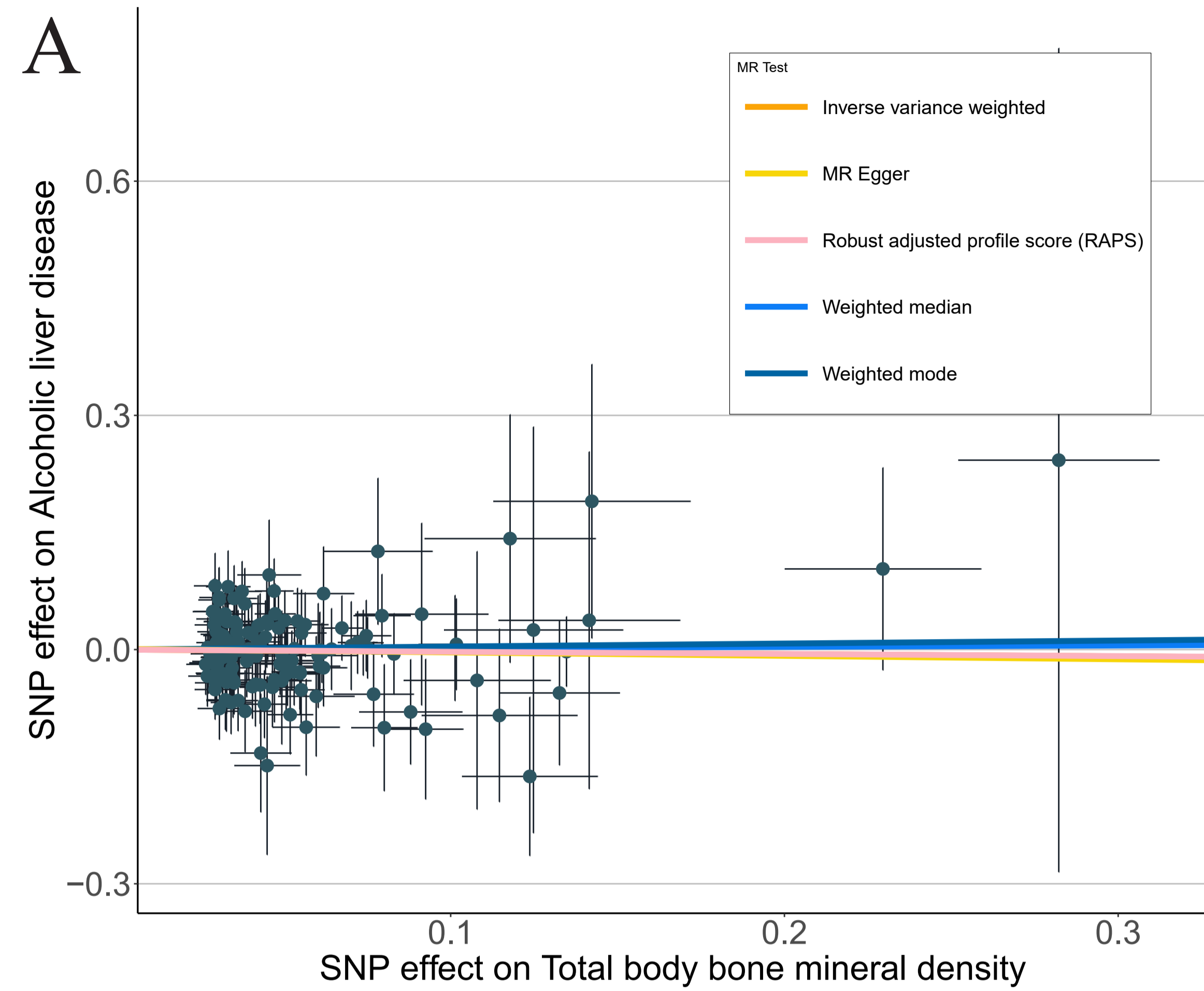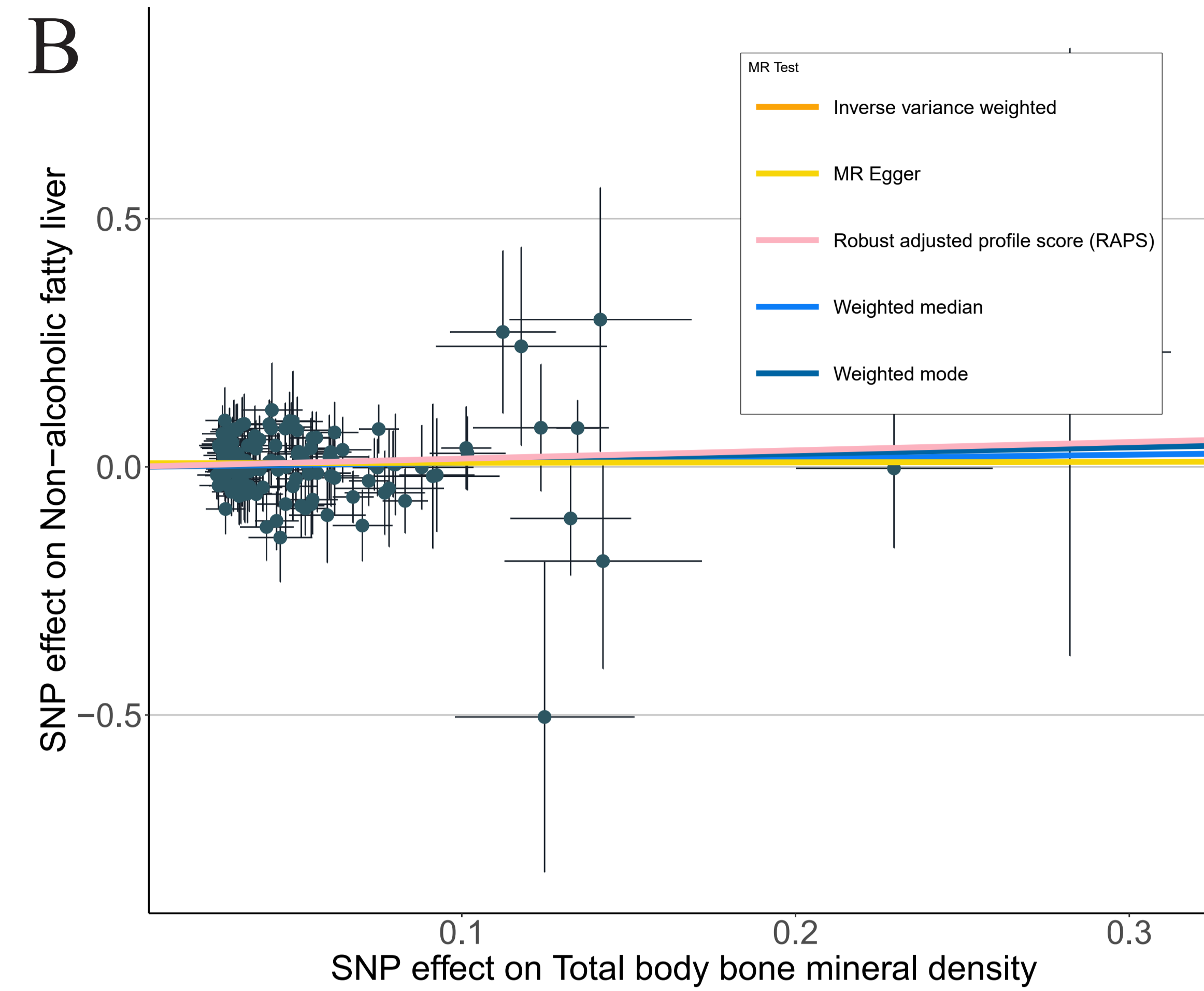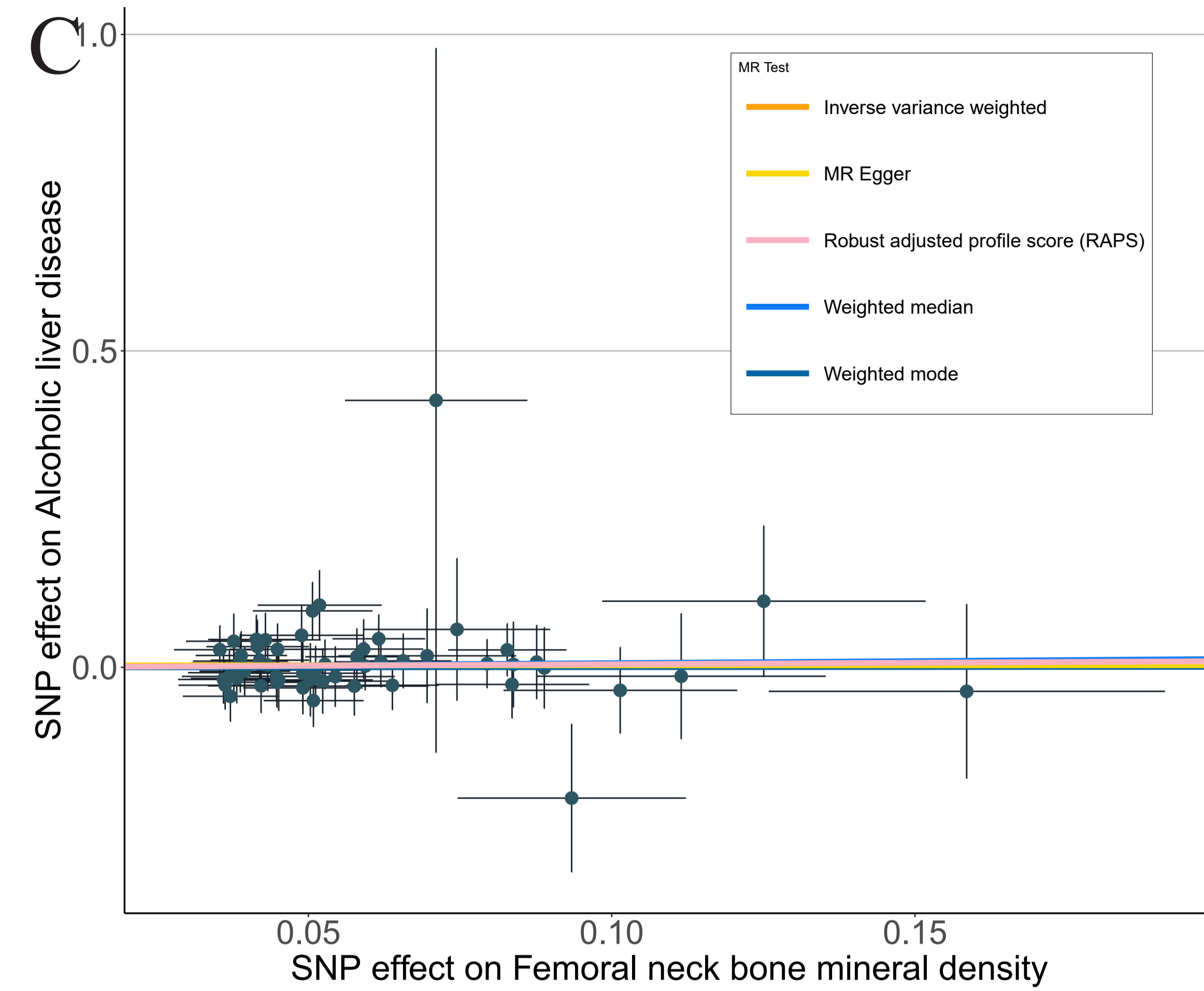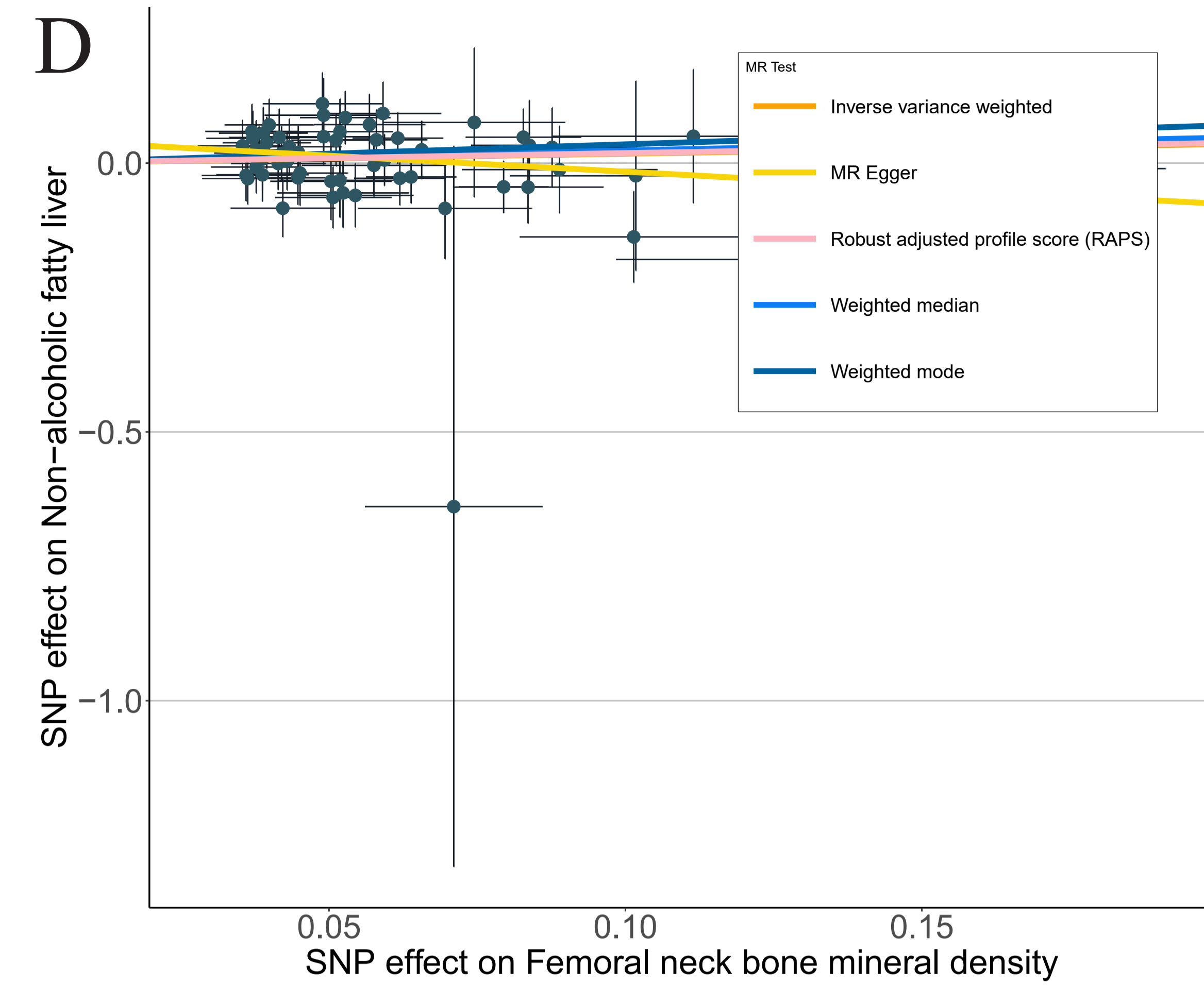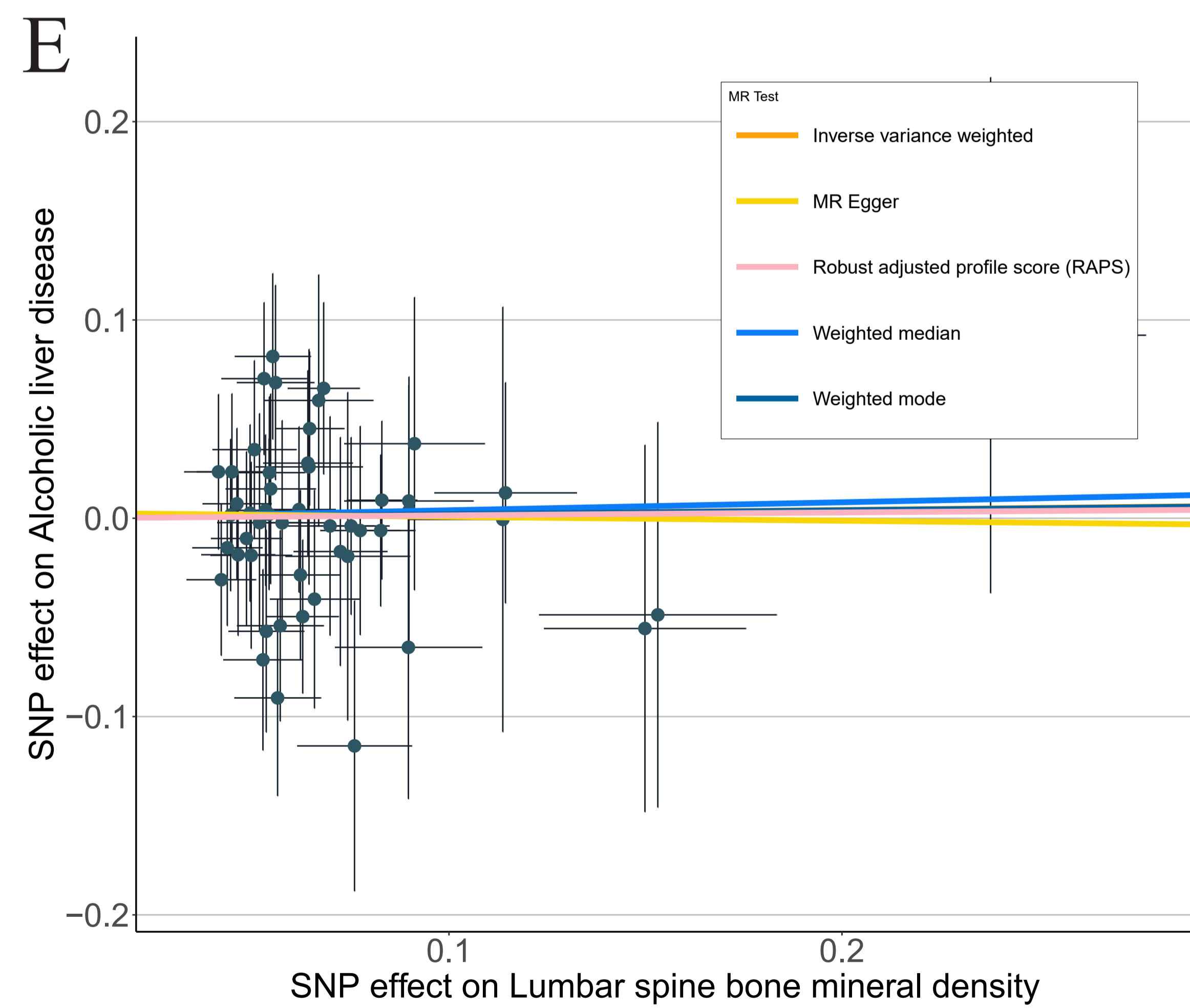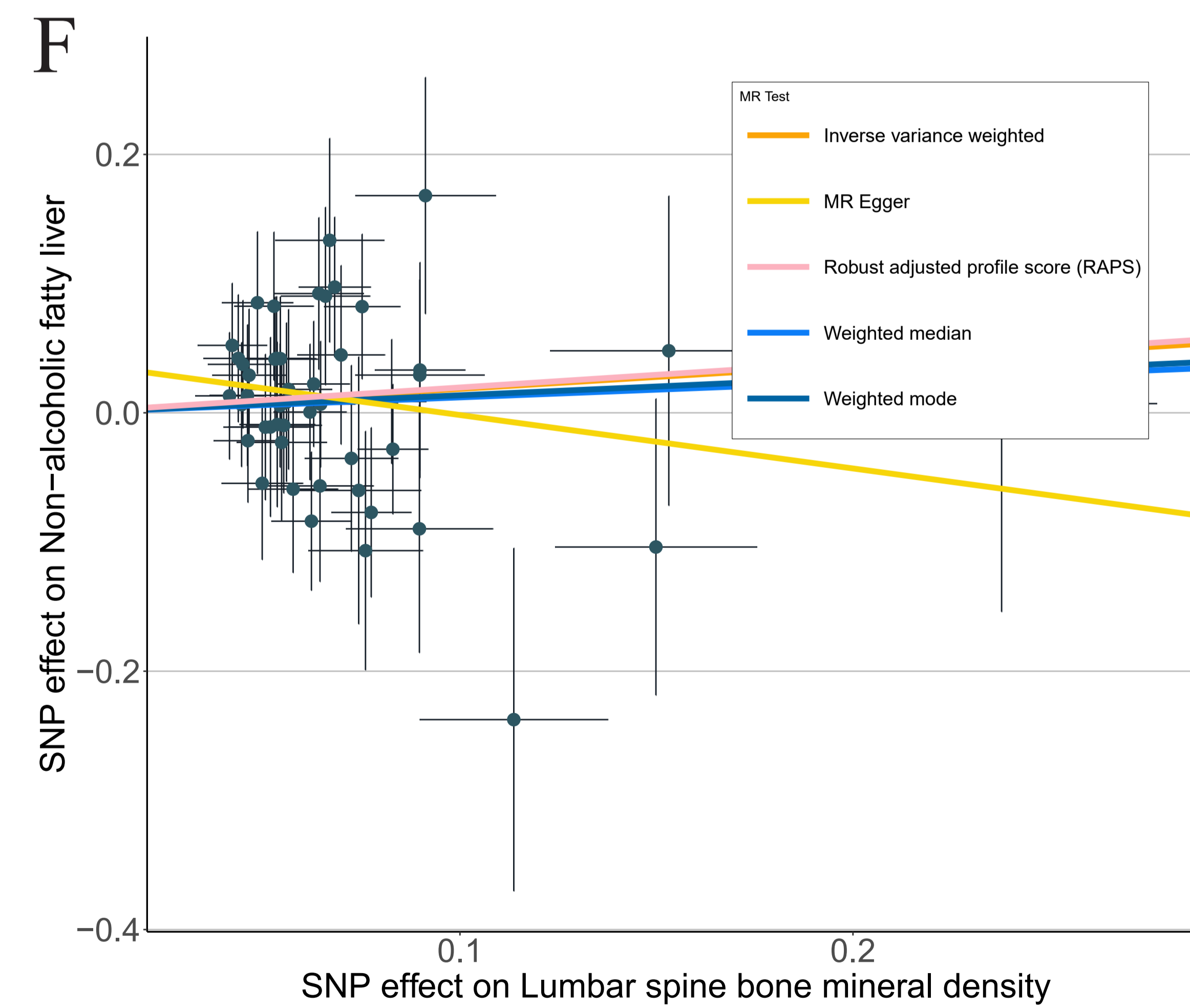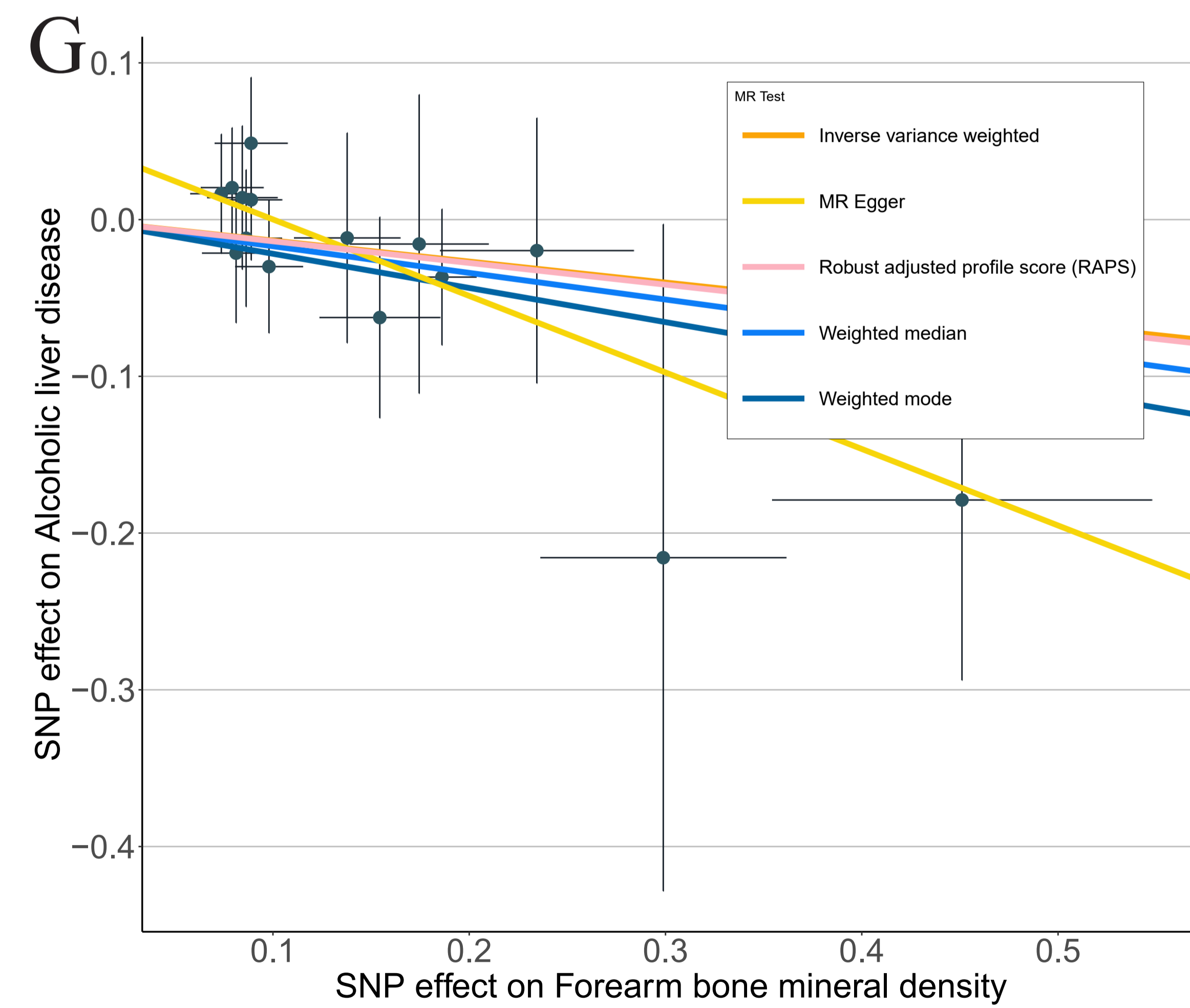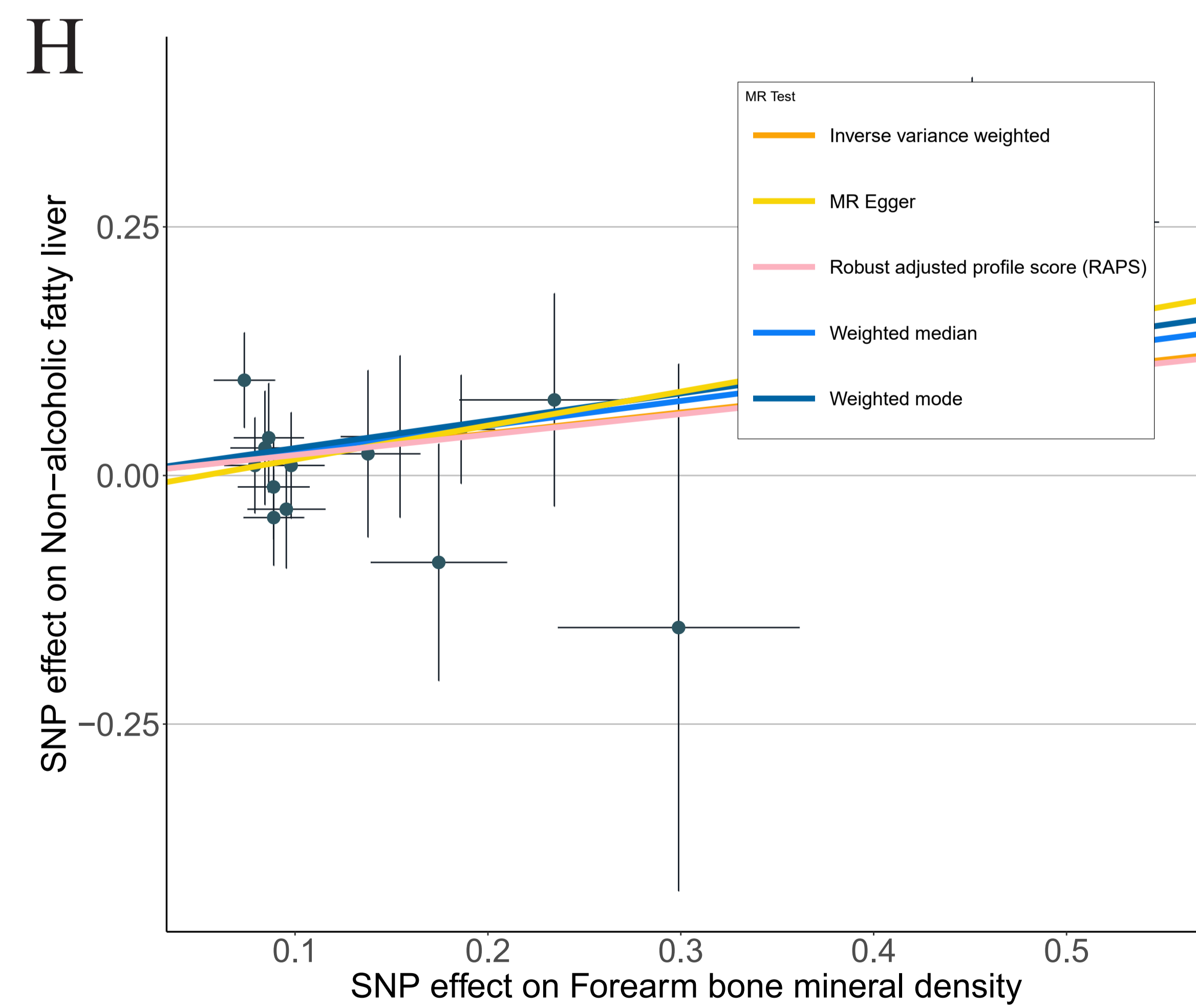

Supplement: S8 Fig — Scatterplot of associations of genetically predicted of TB-BMD on the risk of A)ALD;B) NAFLD; Scatterplot of associations of genetically predicted of FN-BMD on the risk of C)ALD;D) NAFLD; Scatterplot of associations of genetically predicted of LS-BMD on the risk of E)ALD;F) NAFLD; Scatterplot of associations of genetically predicted of FA-BMD on the risk of G)ALD;H) NAFLD. (PDF) [file pone.0292881.s008.pdf]

A

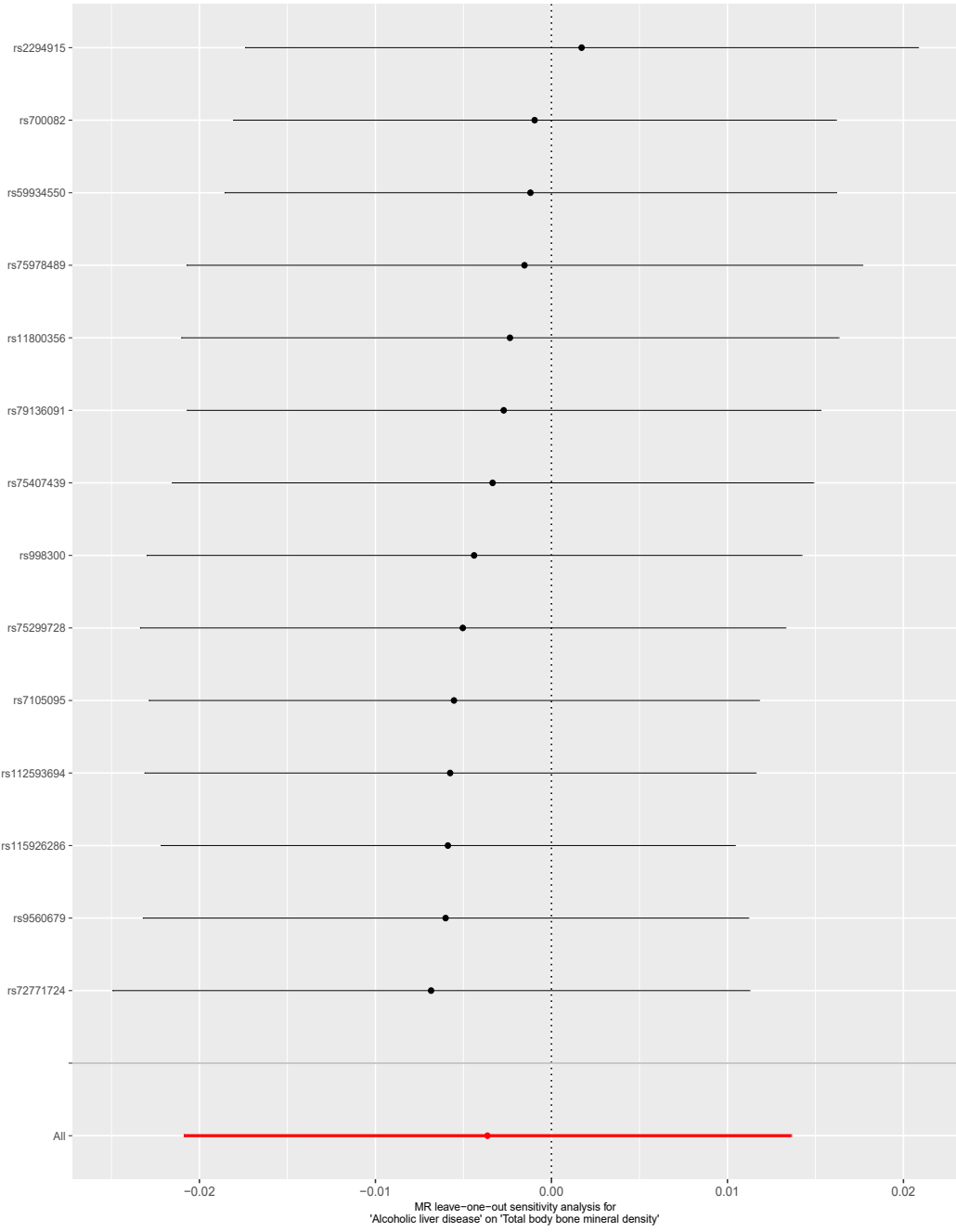

B

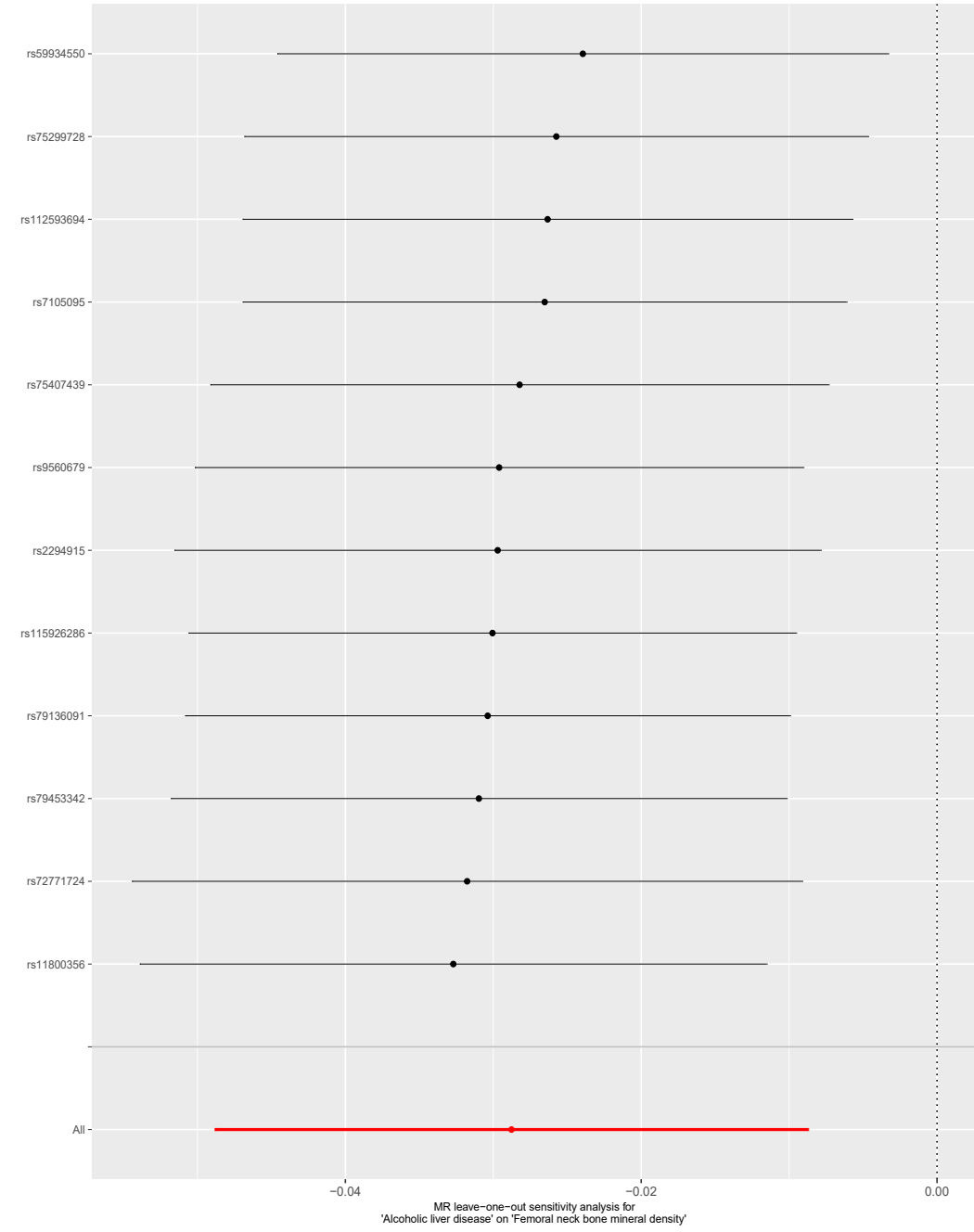

C

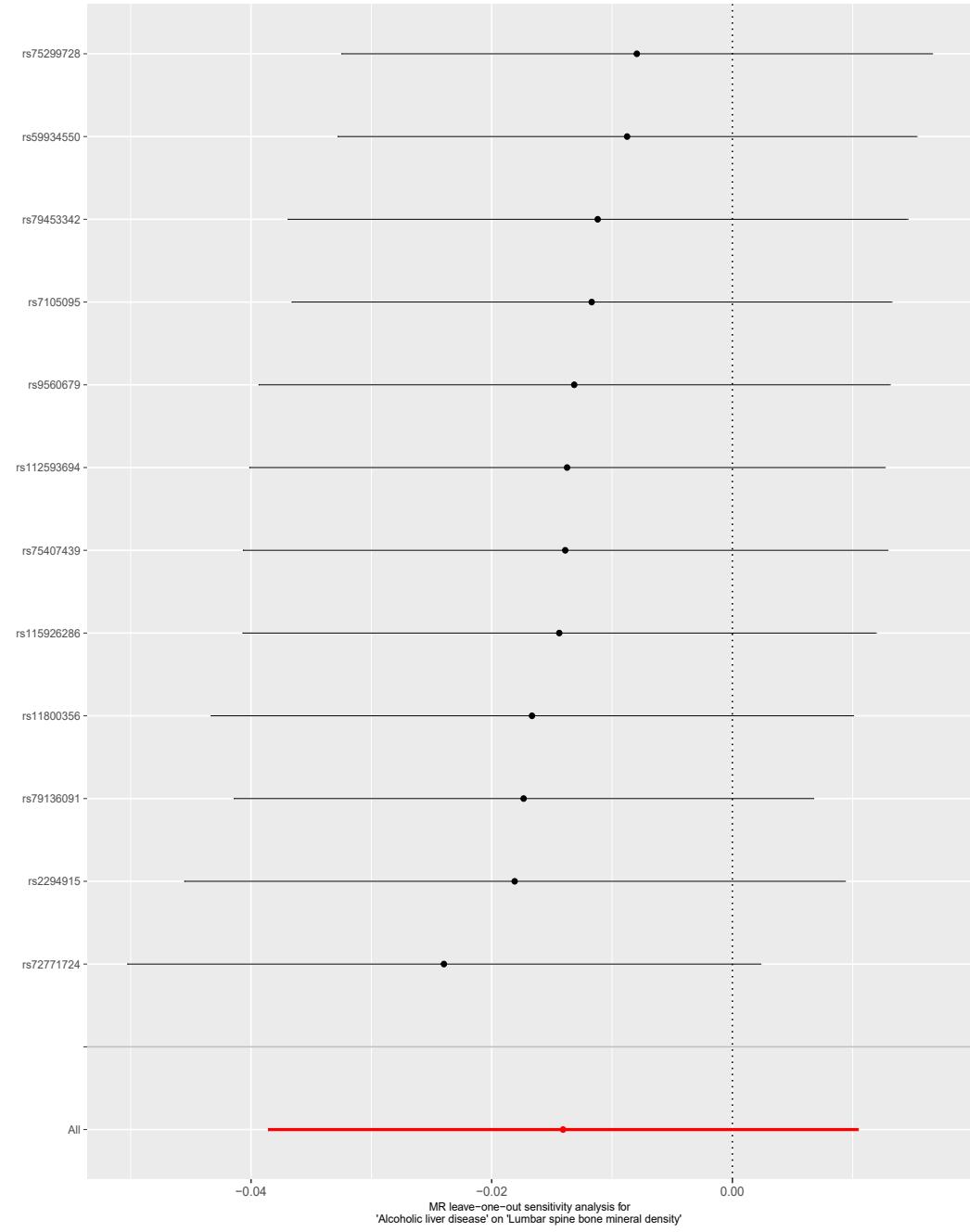

D

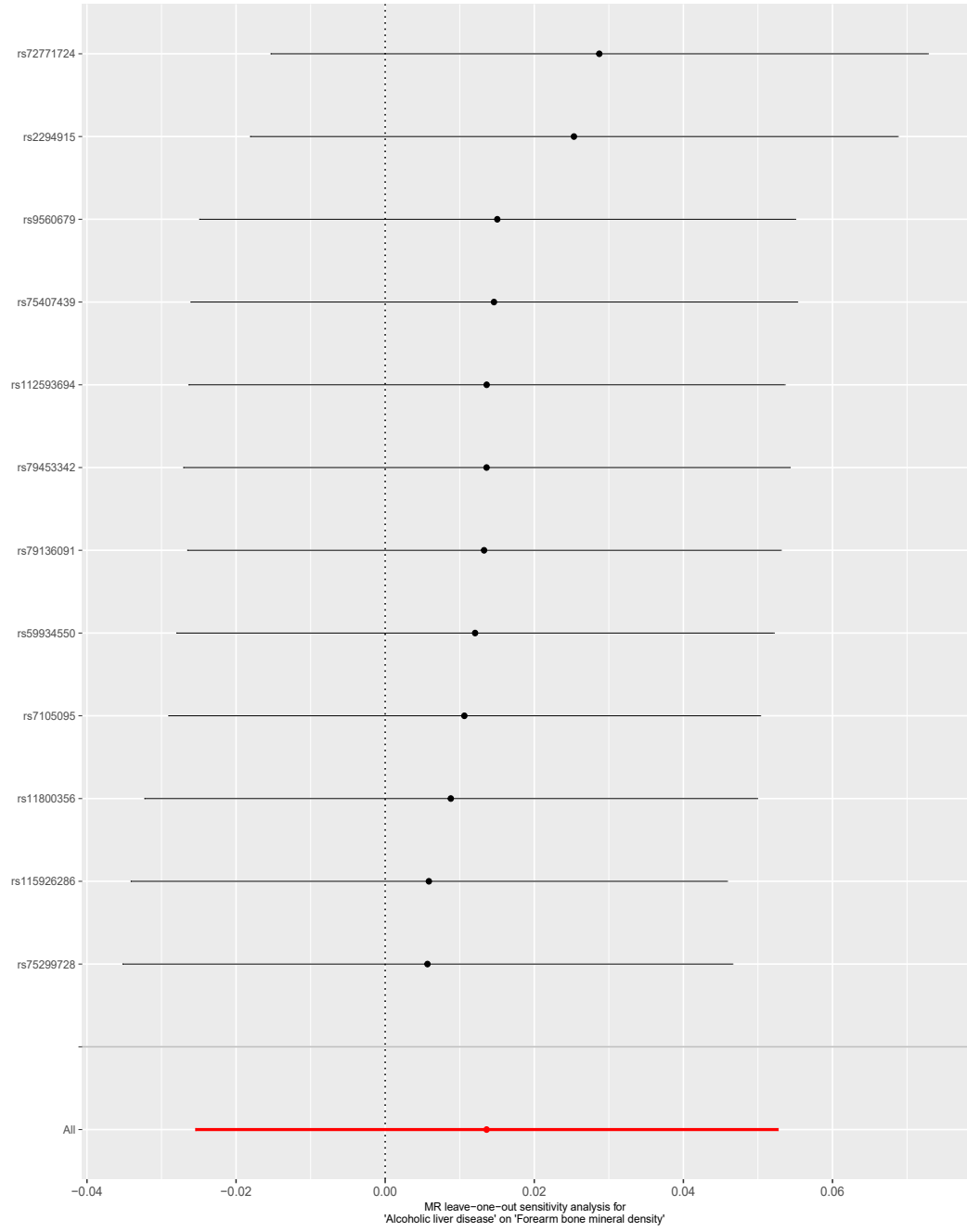

E

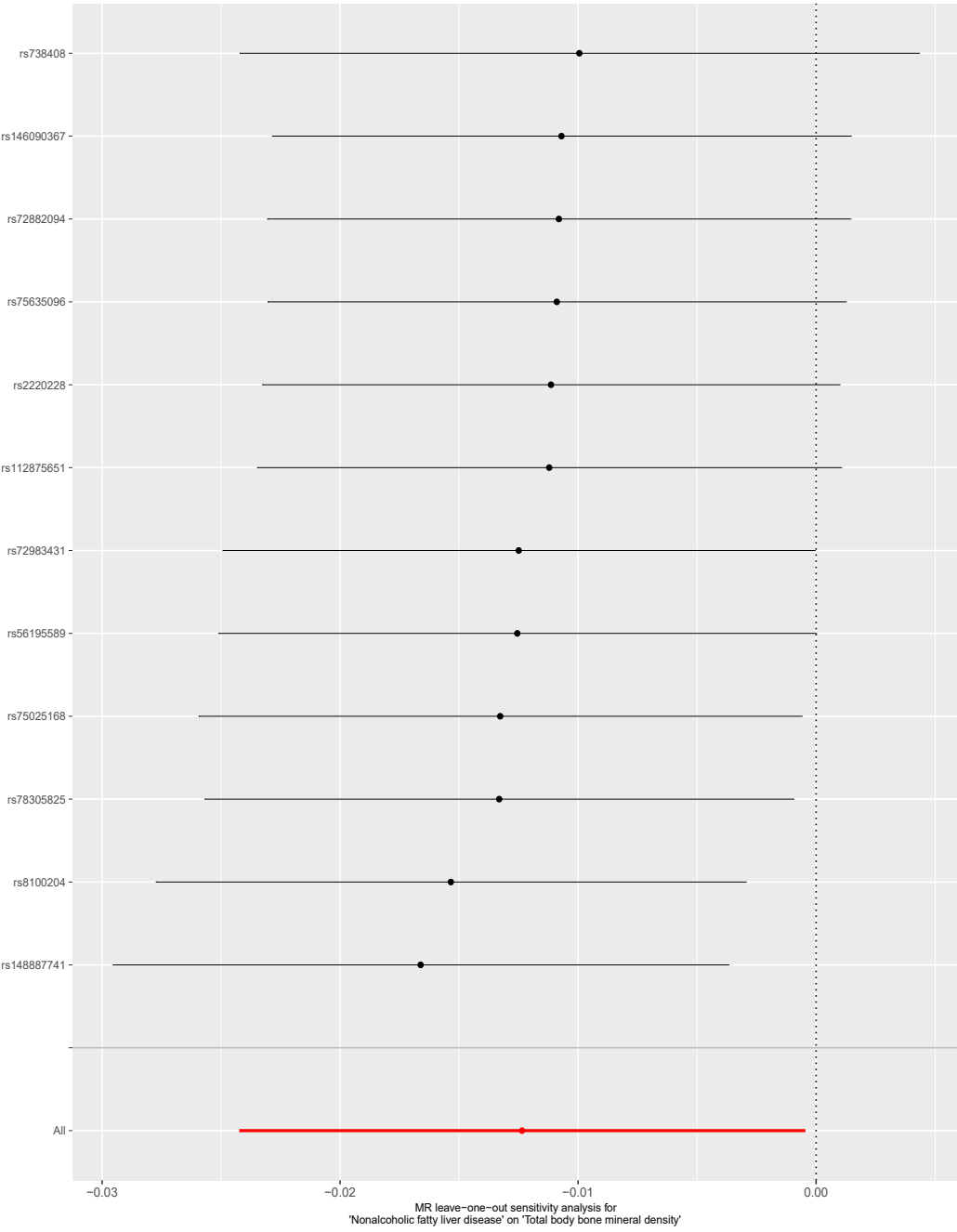

F

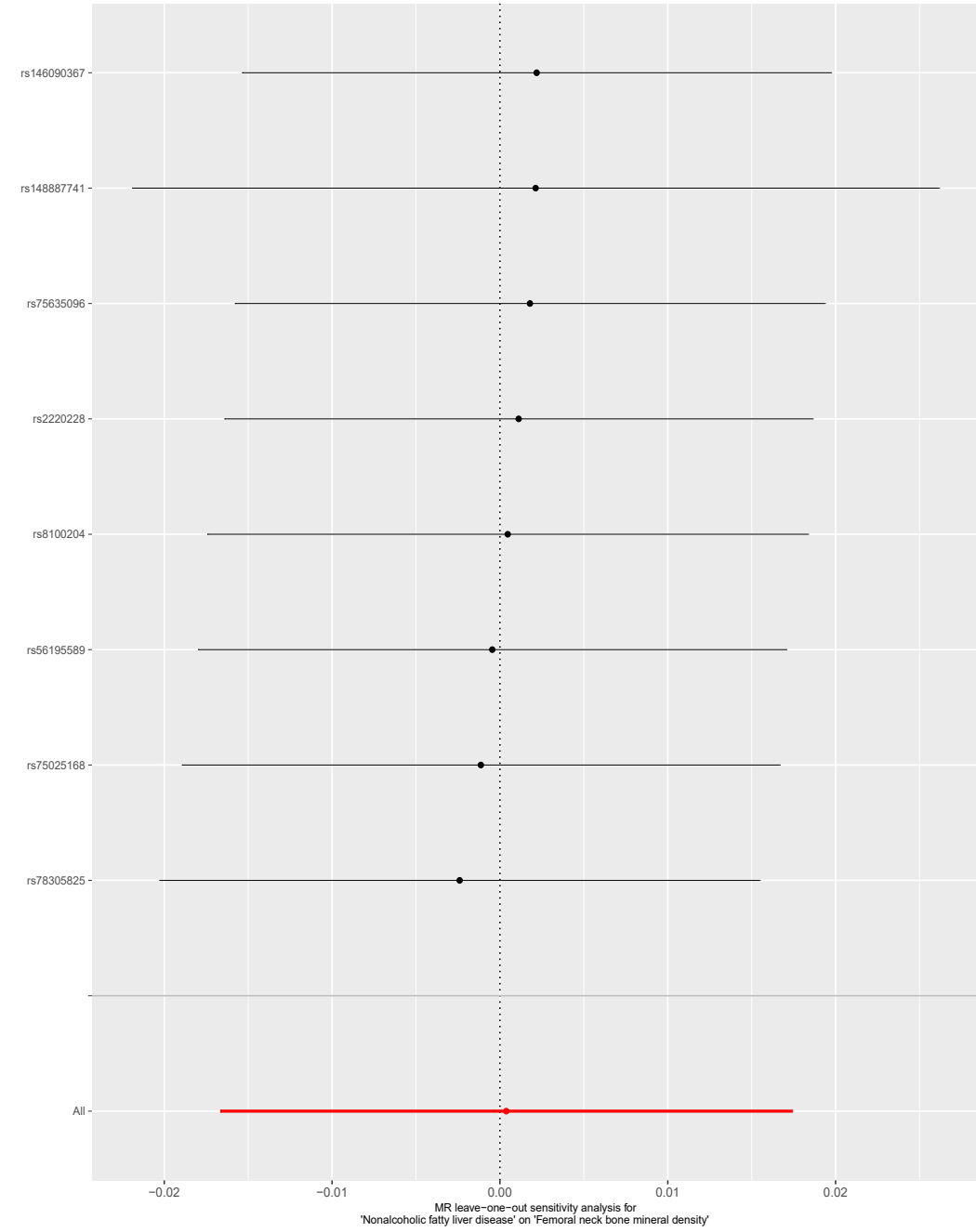

G

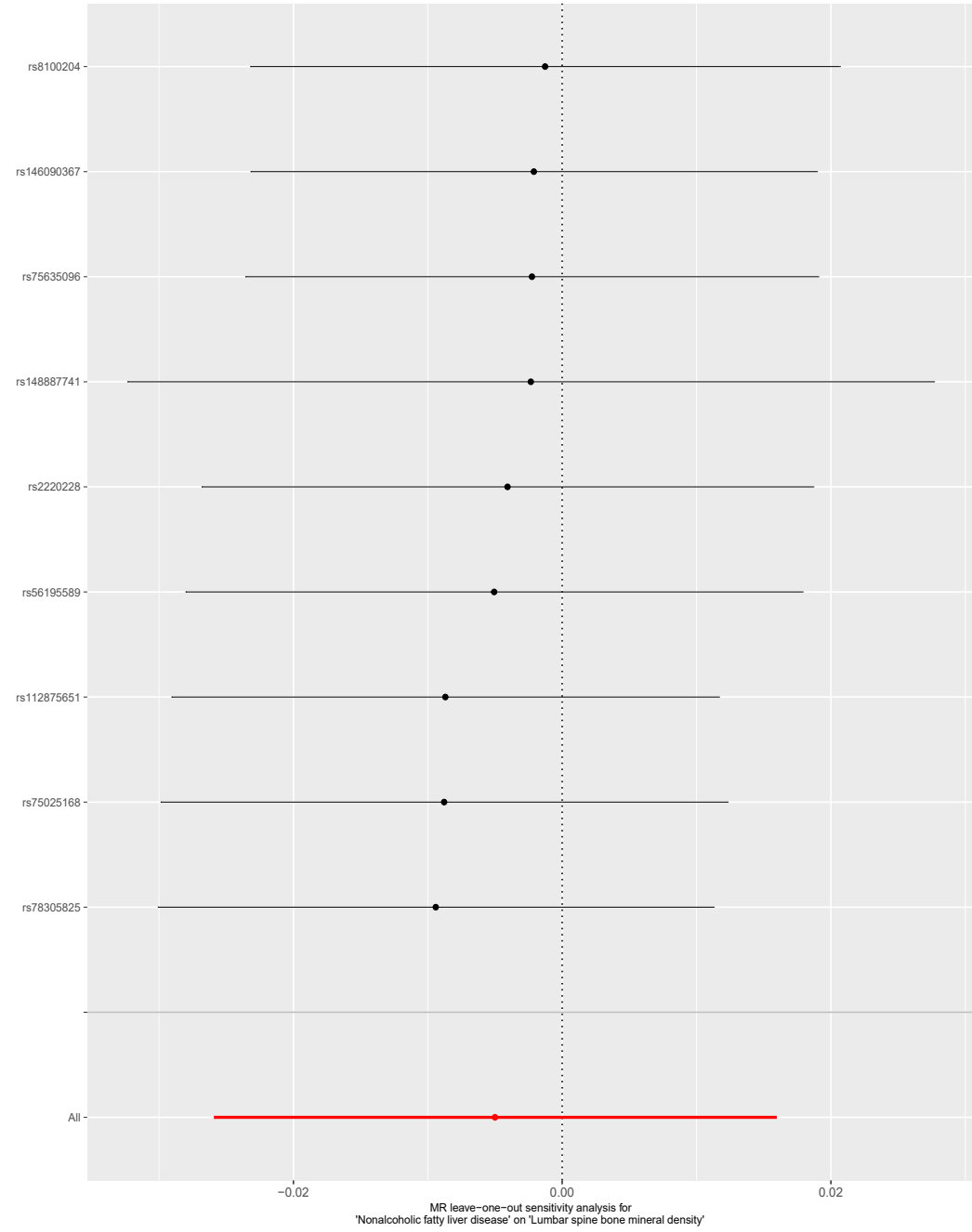

H

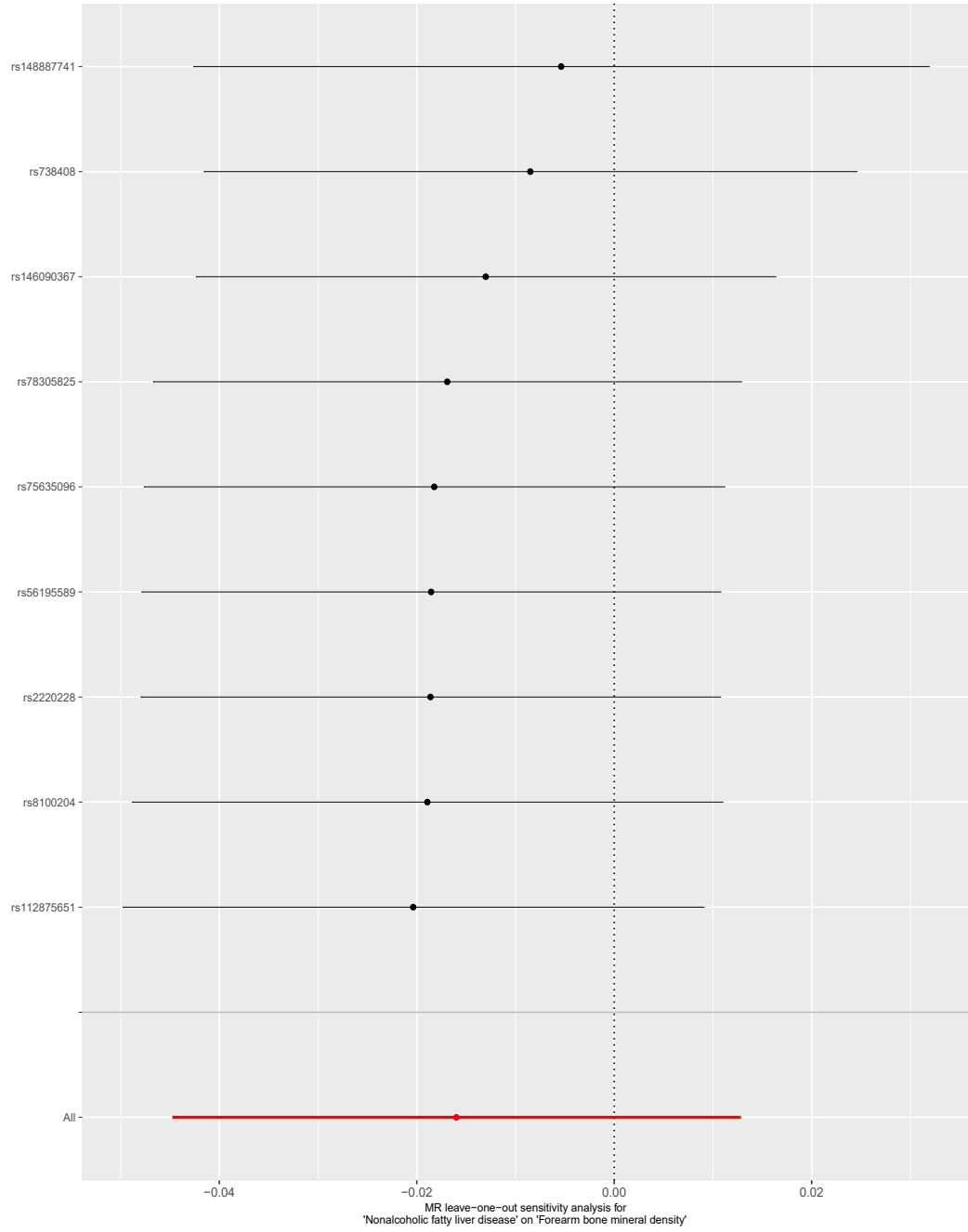

Supplement: S9 Fig — Leave-one-out analysis of ALD on A) TB-BMD; B) FN-BMD; C) LS = BMD; D) FA-BMD; Leave-one-out analysis of NAFLD on E) TB-BMD; F) FN-BMD; G) LS = BMD; H) FA-BMD. (PDF) [file pone.0292881.s009.pdf]

A

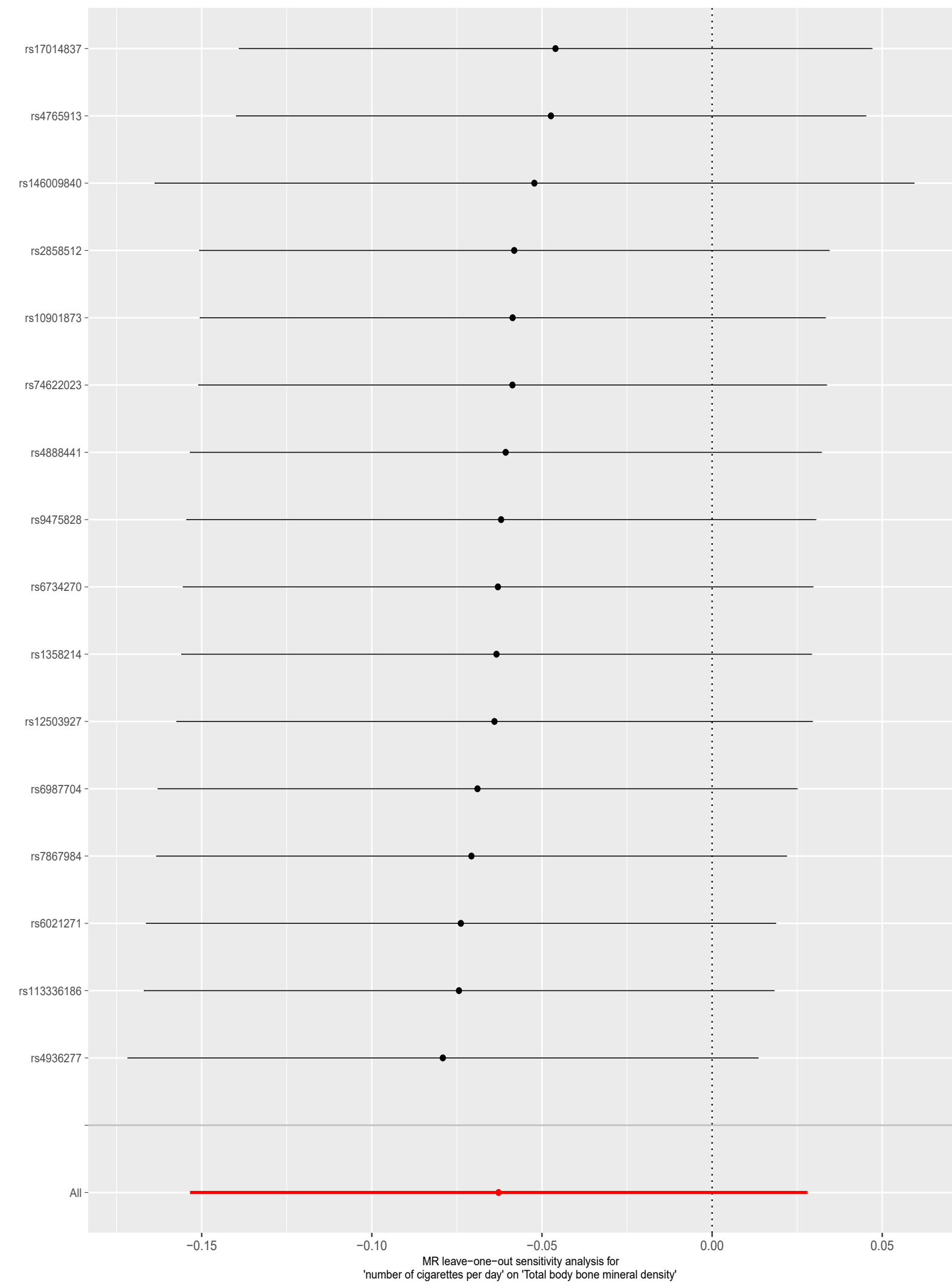

B

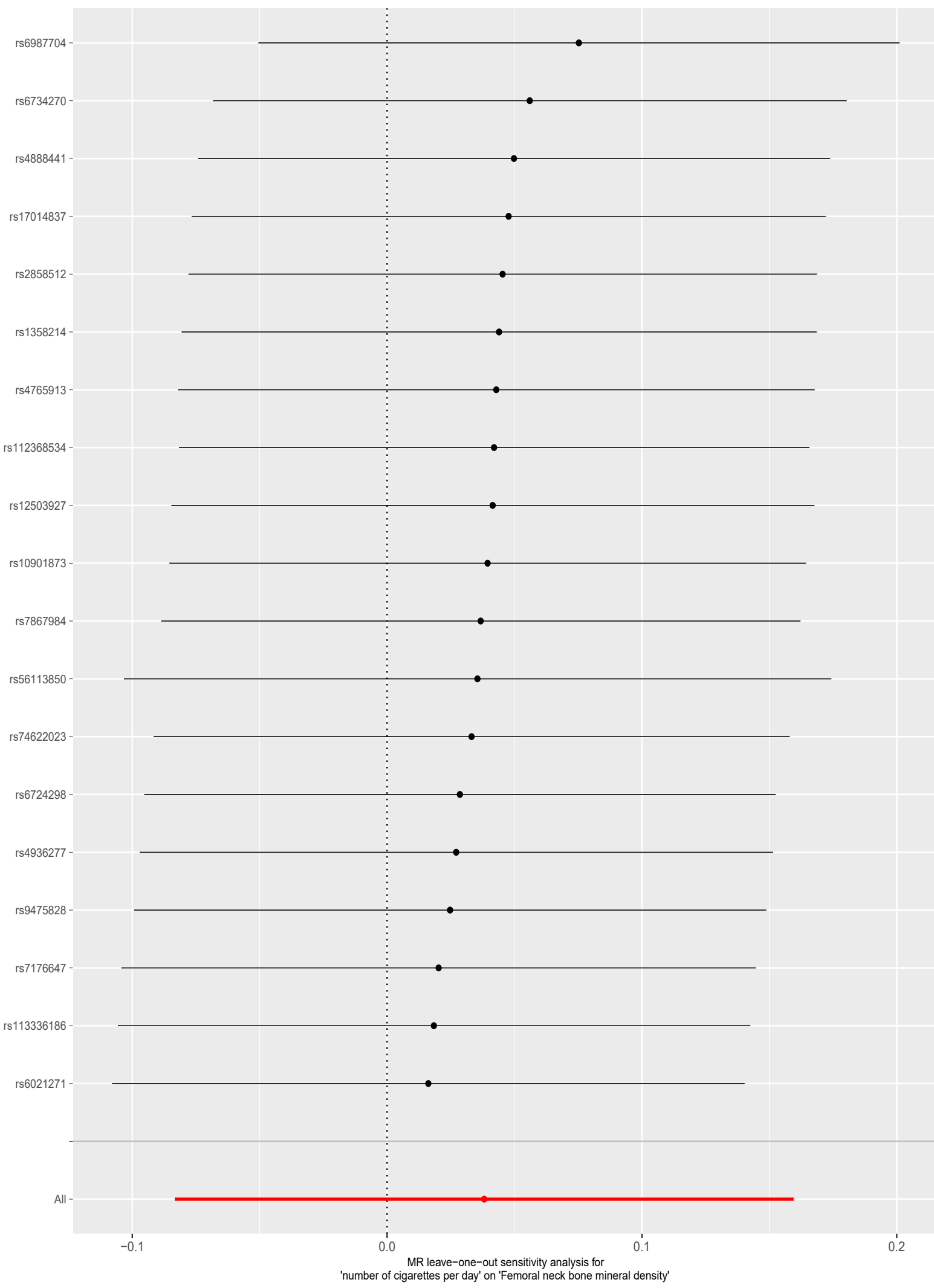

C

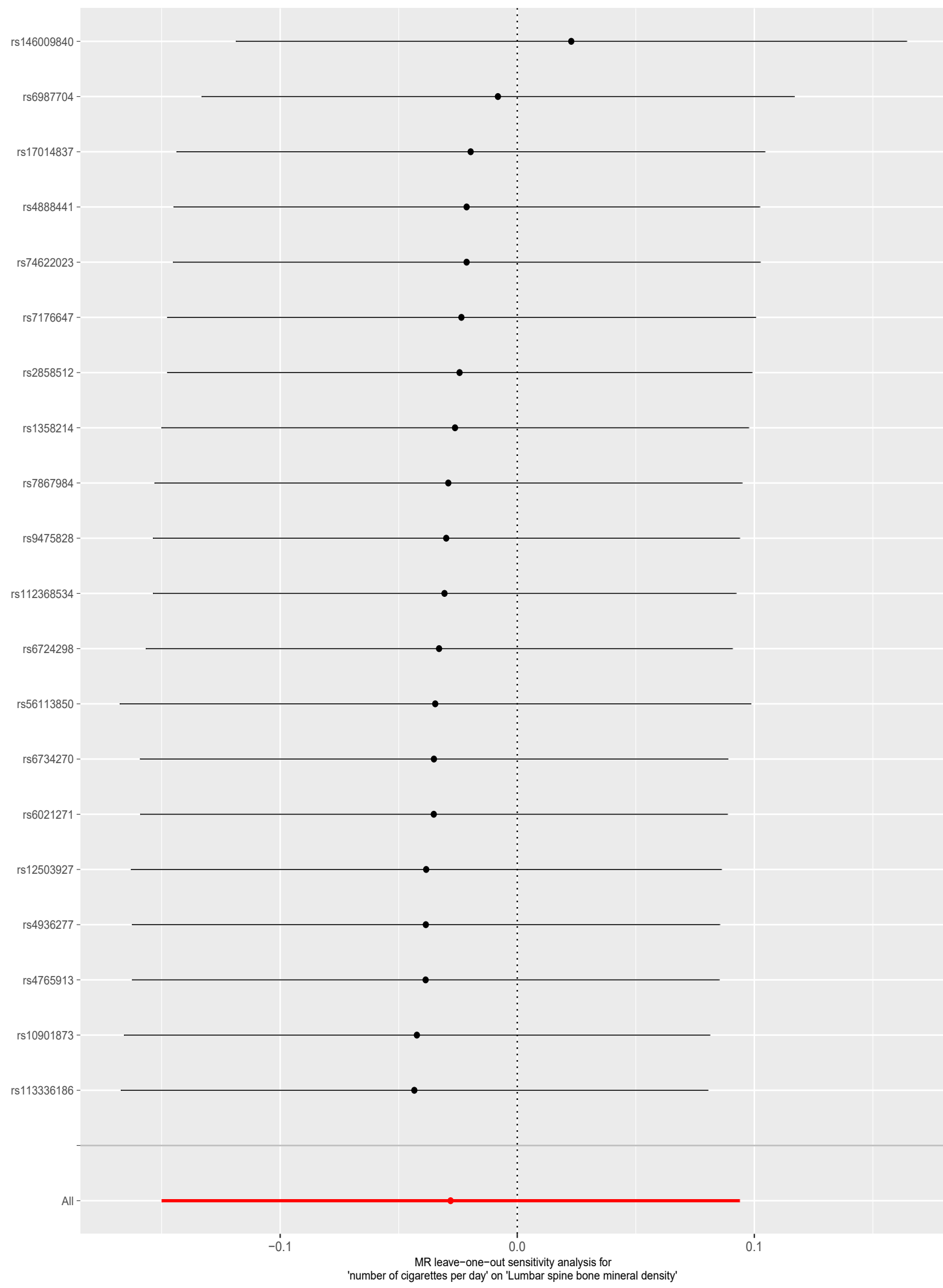

D

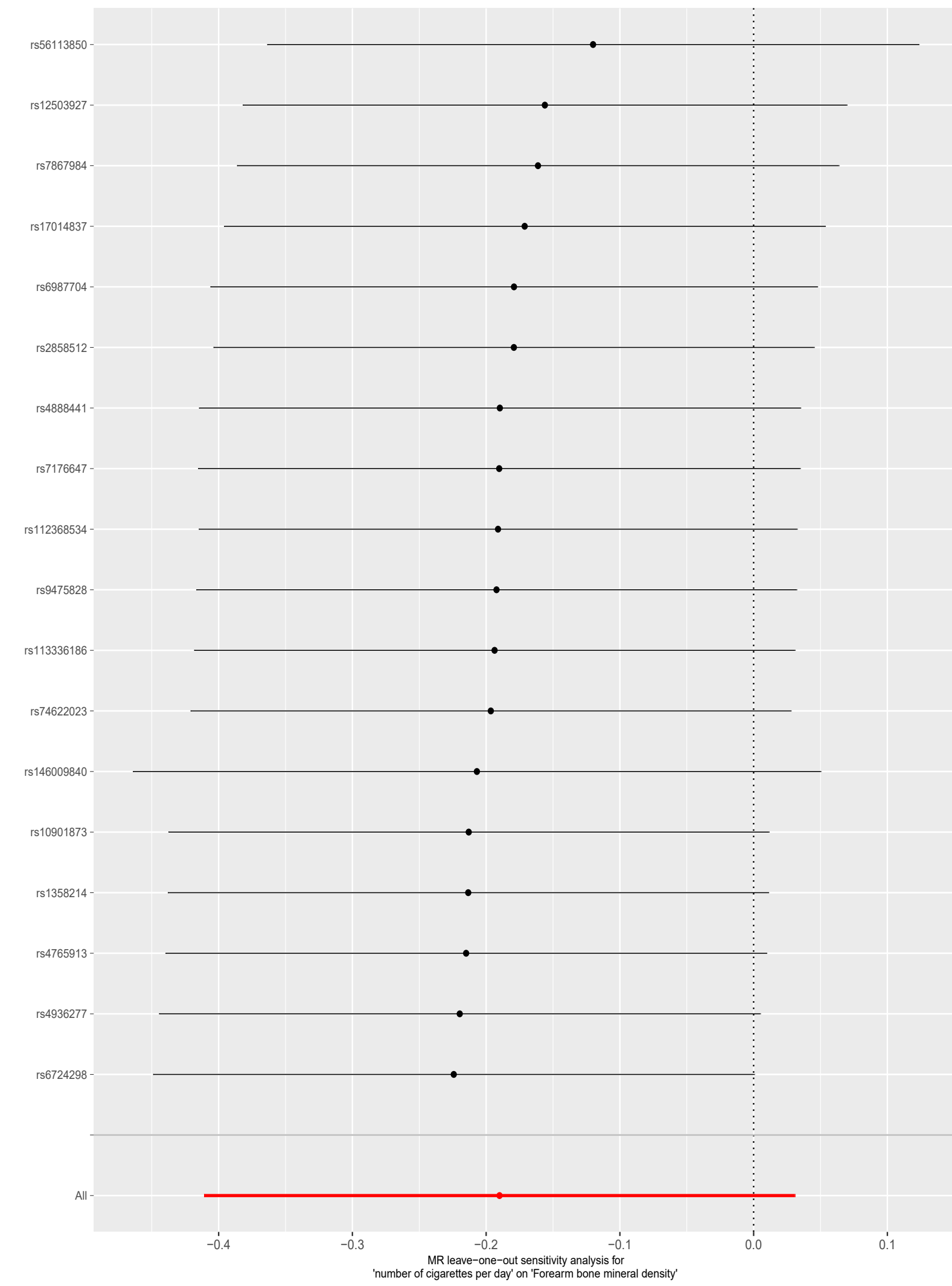

E

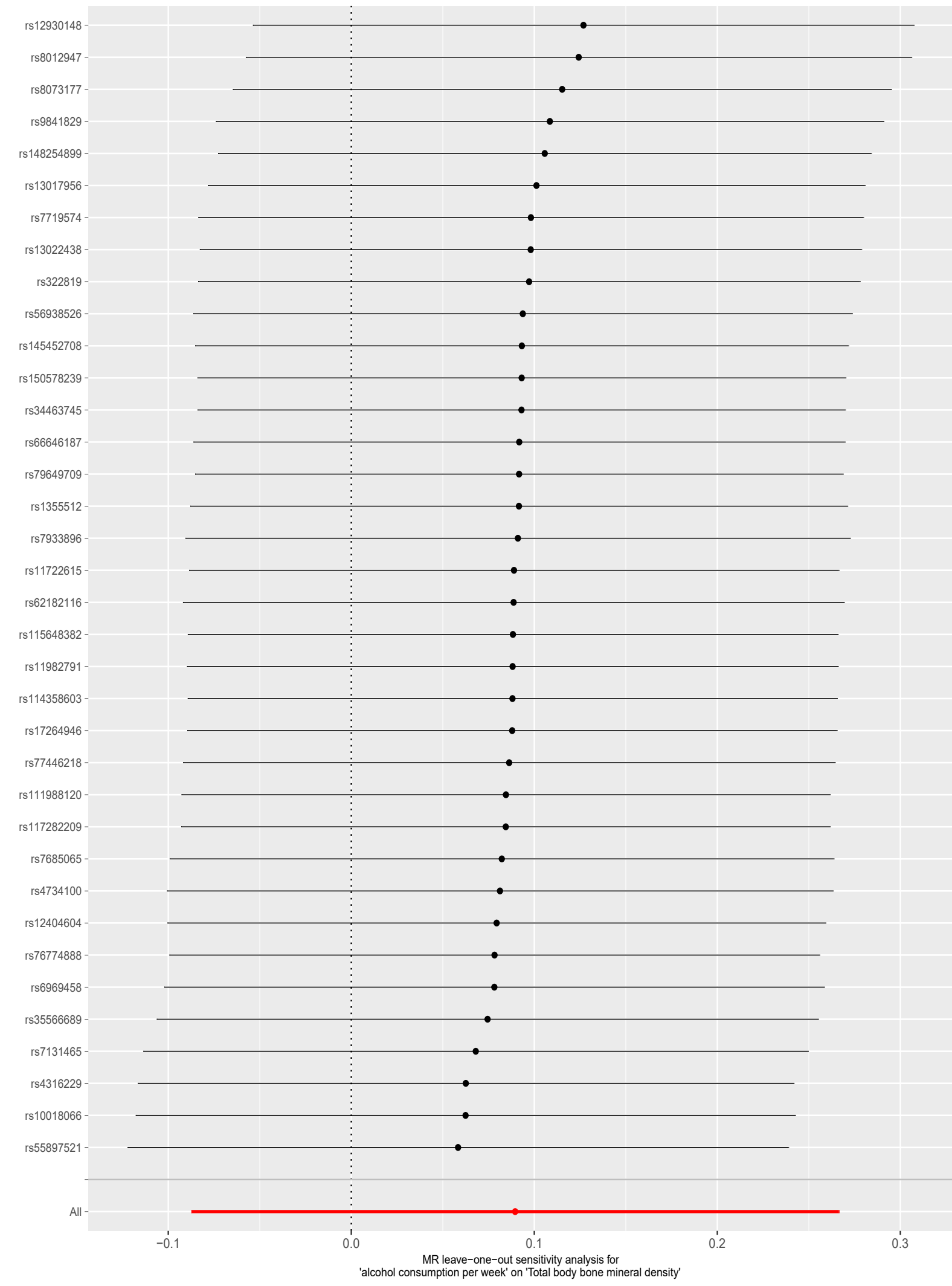

F

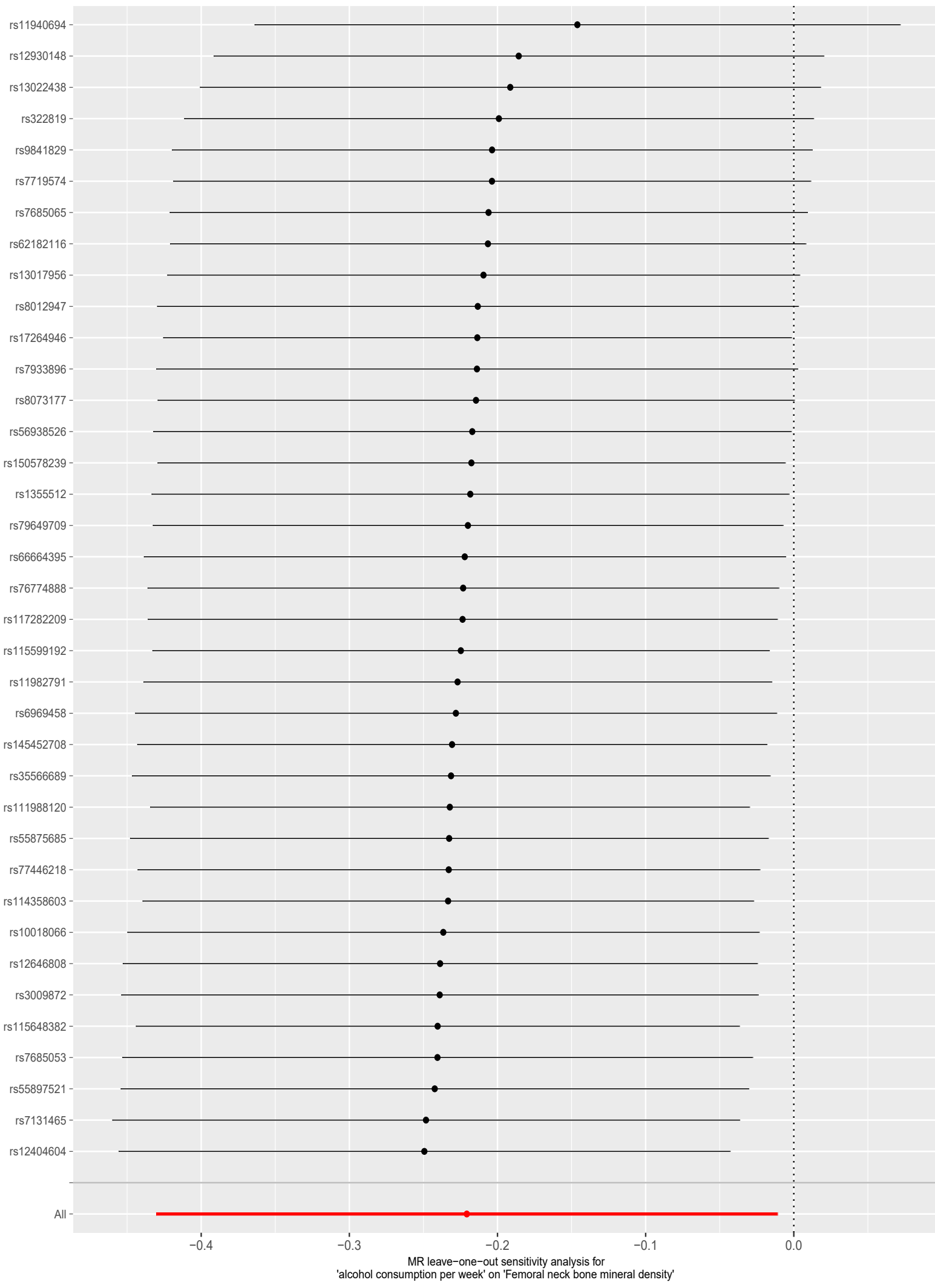

G

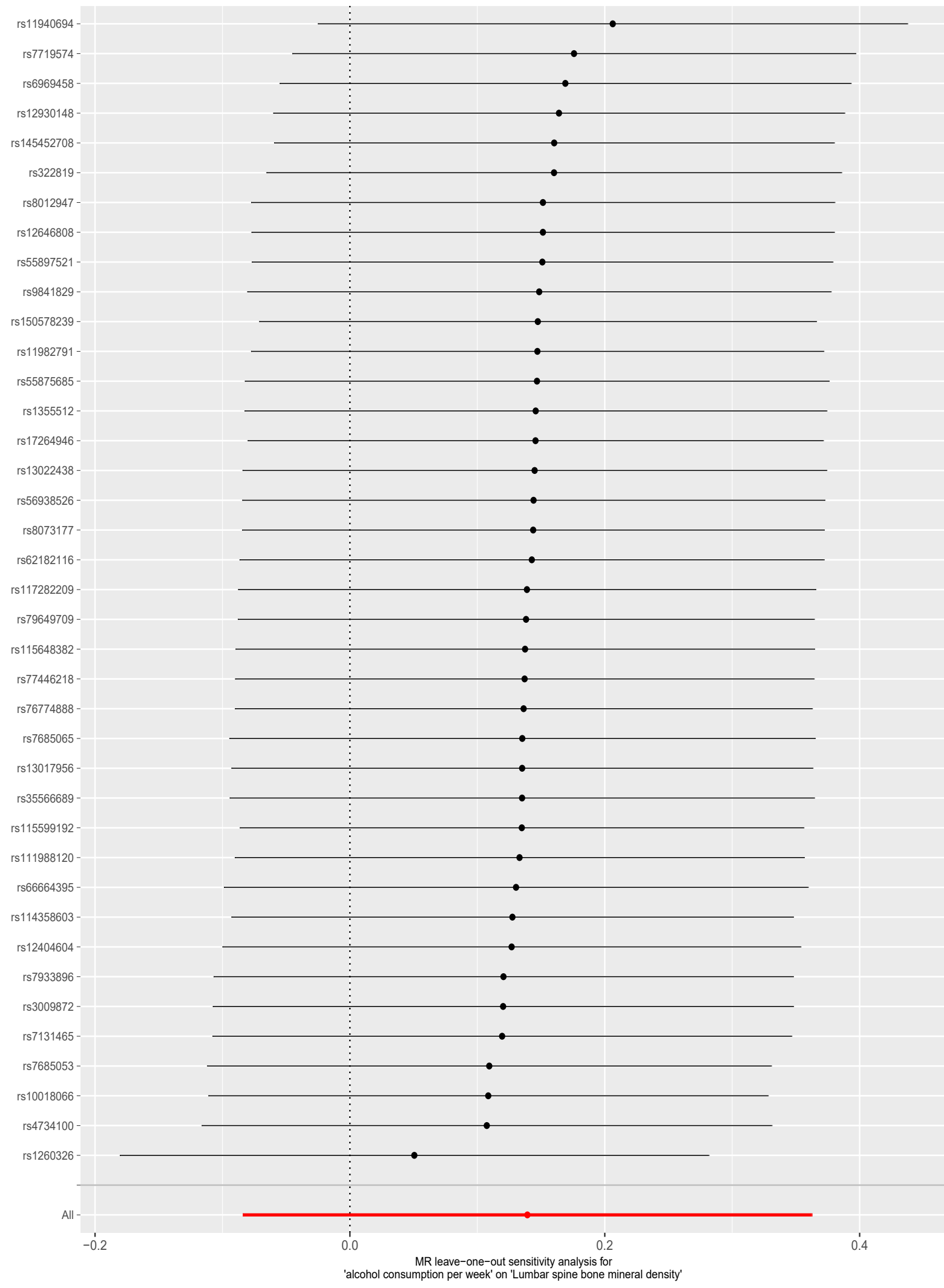

H

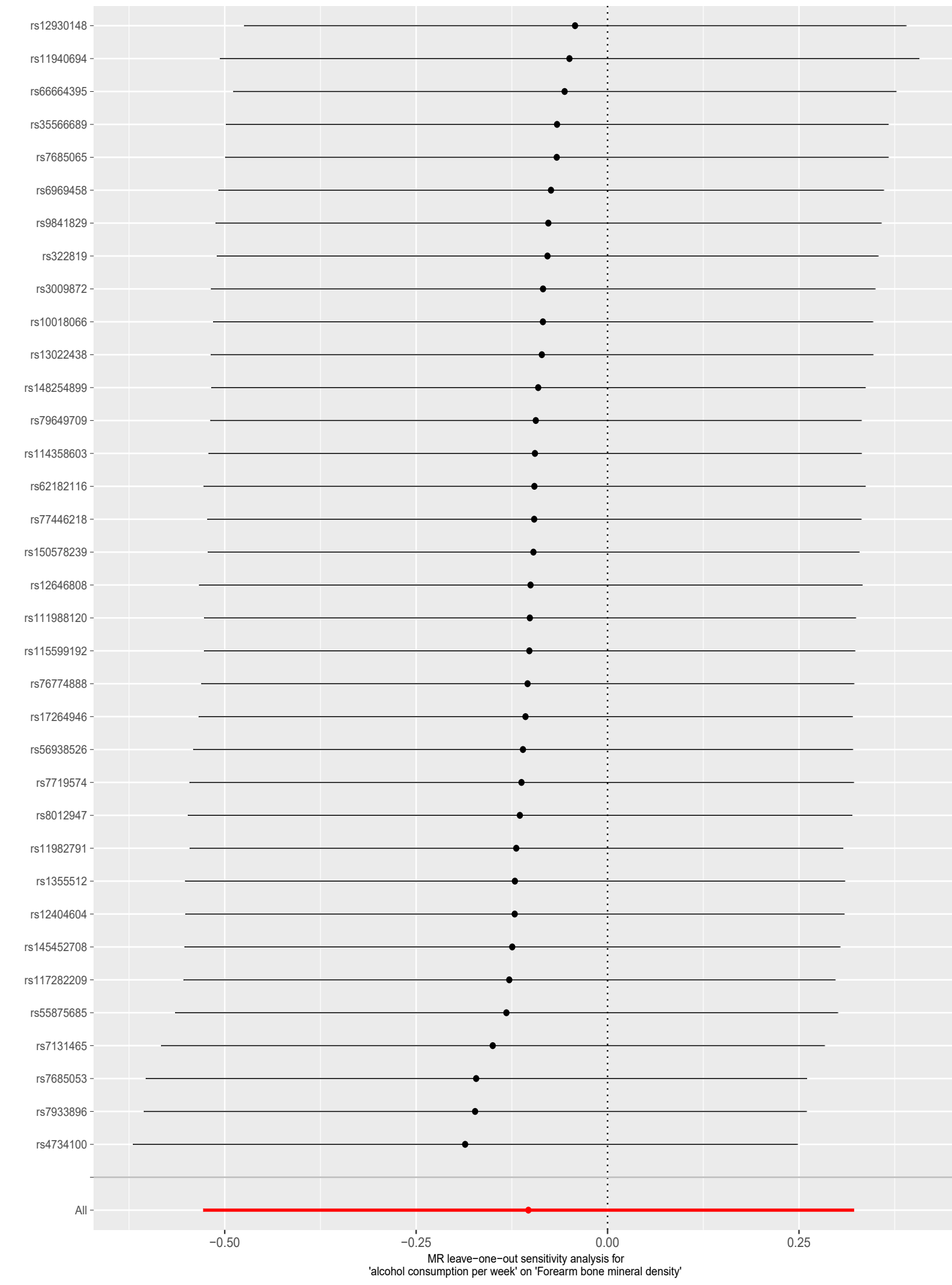

Supplement: S10 Fig — Leave-one-out analysis of number of cigarettes per day on A) TB-BMD; B) FN-BMD; C) LS-BMD; D) FA-BMD; Leave-one-out analysis of alcohol consumption per week on E) TB-BMD; F) FN-BMD; G) LS-BMD; H) FA-BMD. (PDF) [file pone.0292881.s010.pdf]

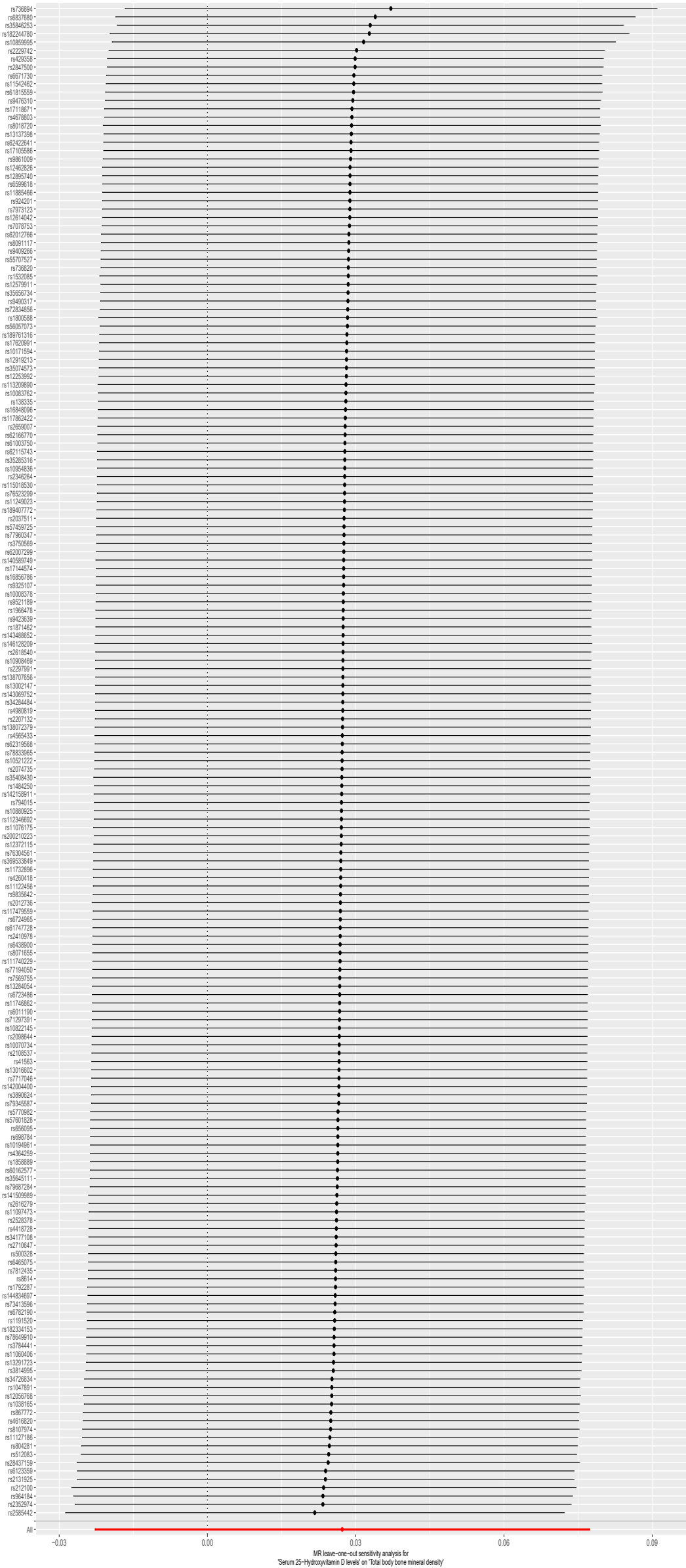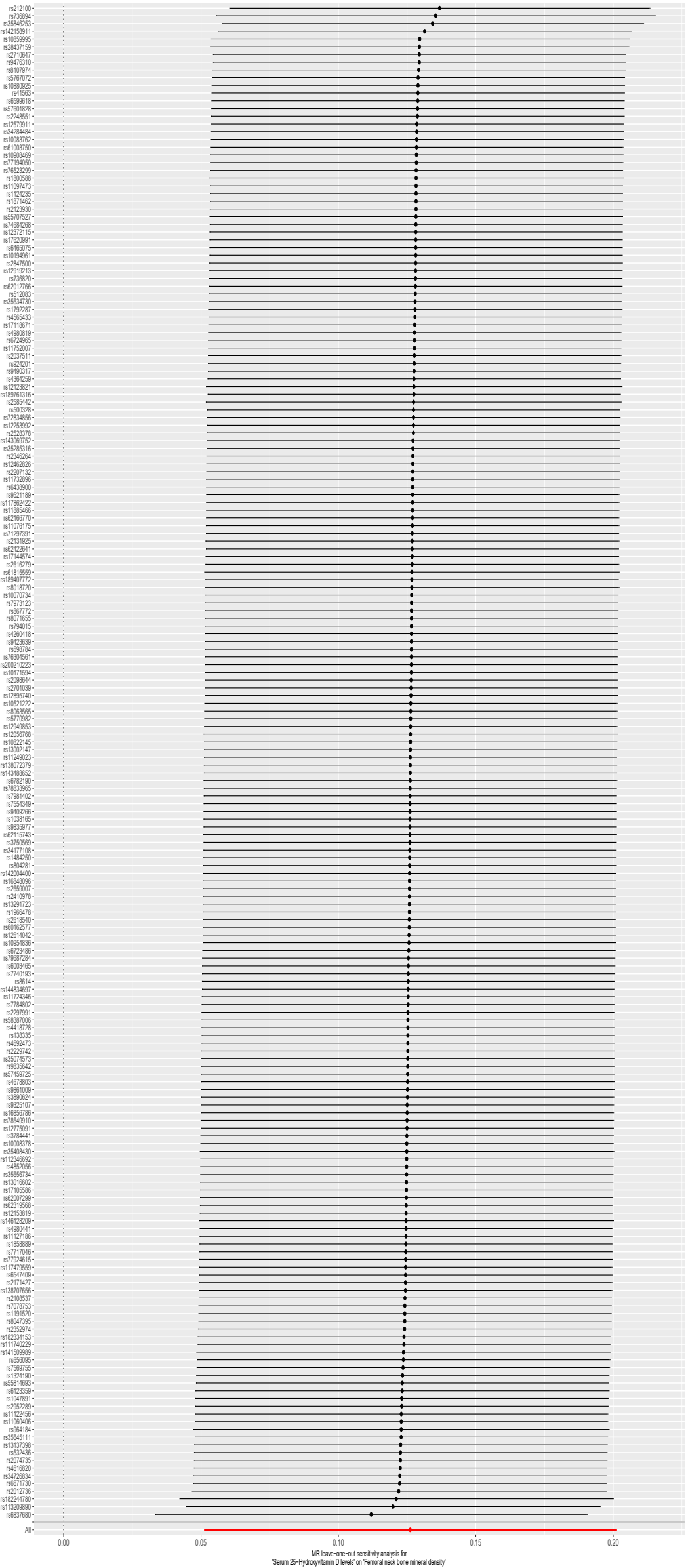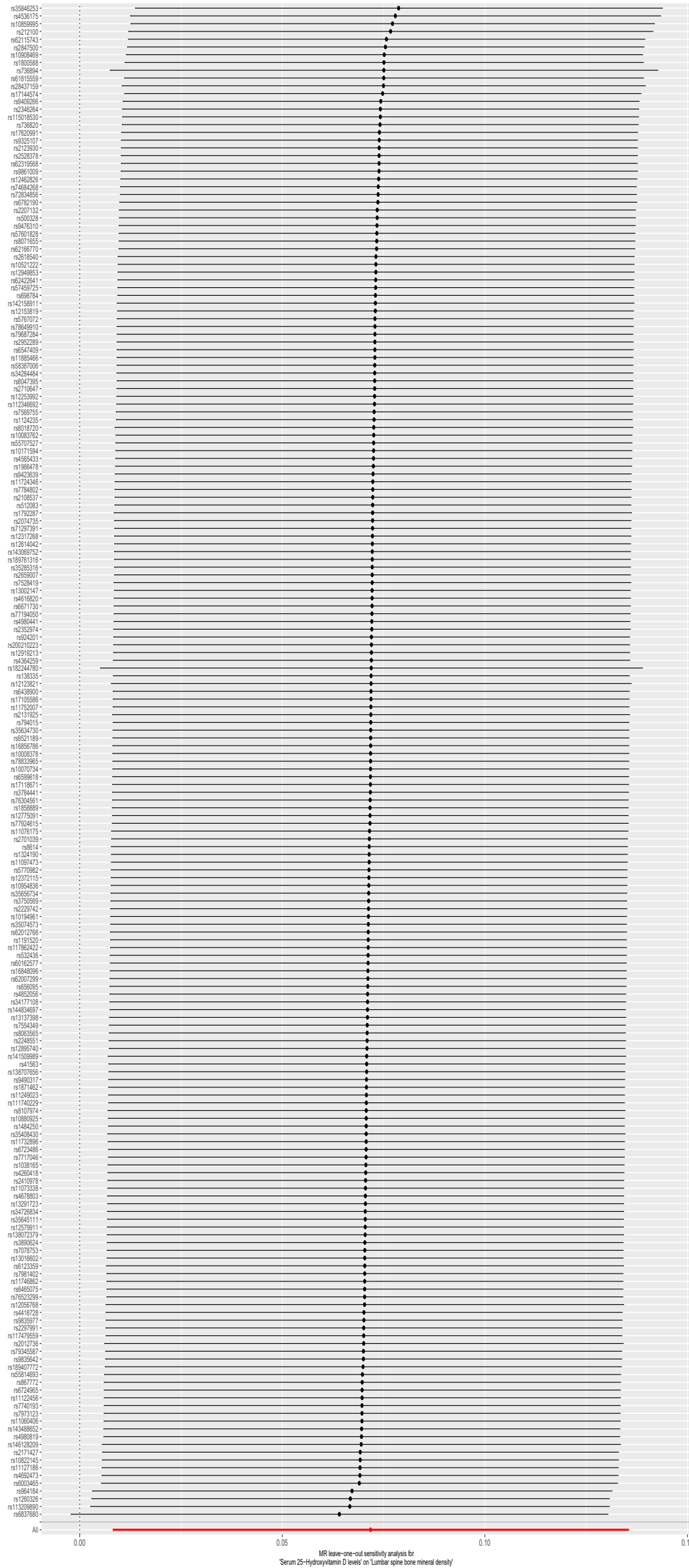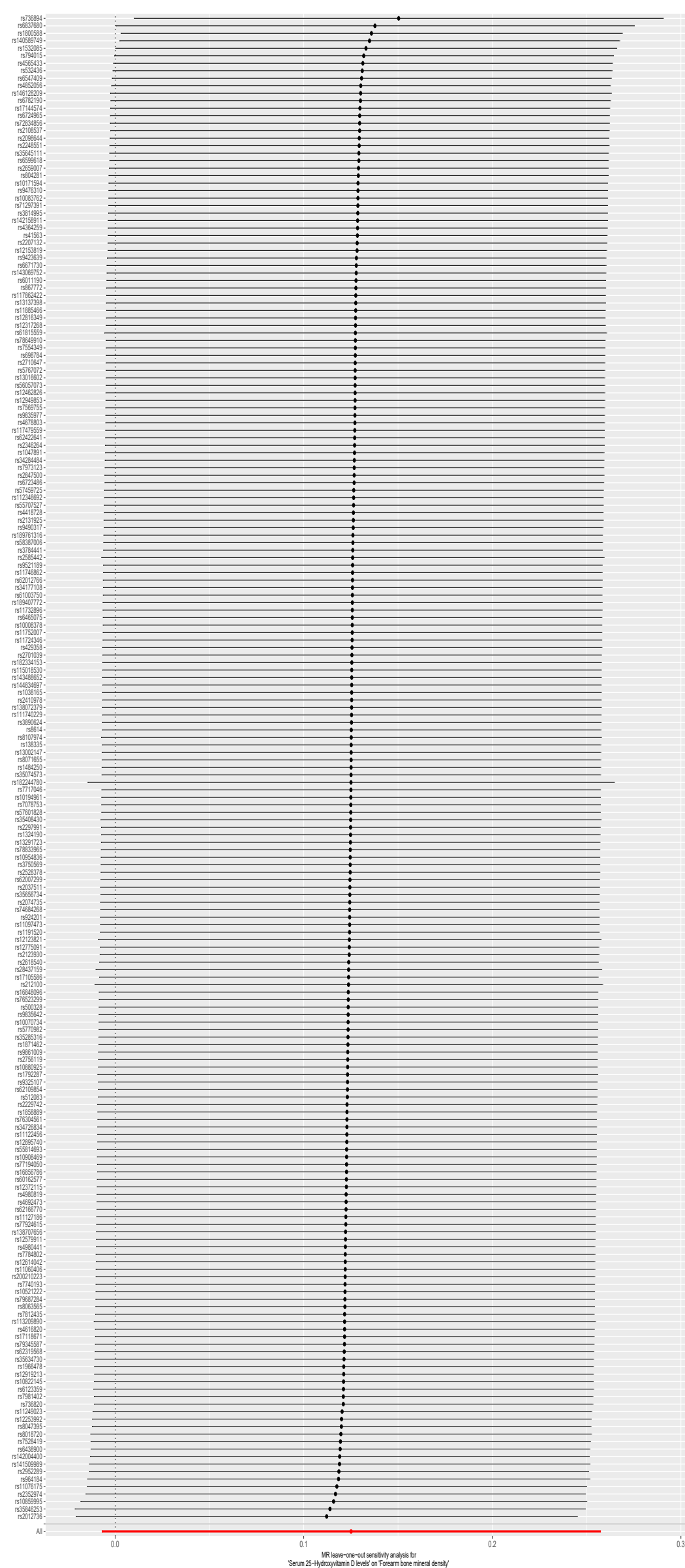

Supplement: S11 Fig — Leave-one-out analysis of serum 25-Hydroxyvitamin D levels on A) TB-BMD; B) FN-BMD; C) LS-BMD; D) FA-BMD. (PDF) [file pone.0292881.s011.pdf]

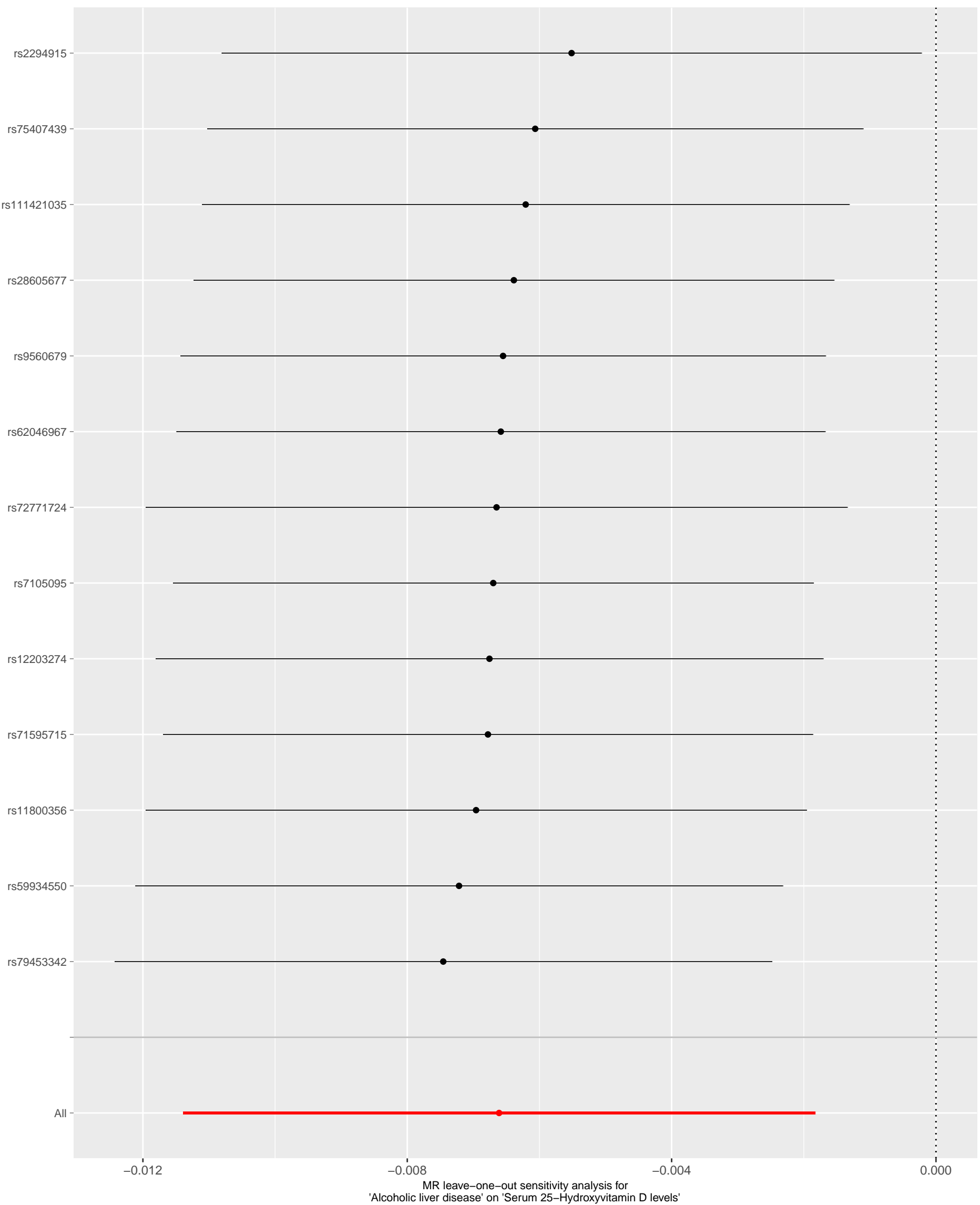

Supplement: S12 Fig — (PDF) [file pone.0292881.s012.pdf]

A

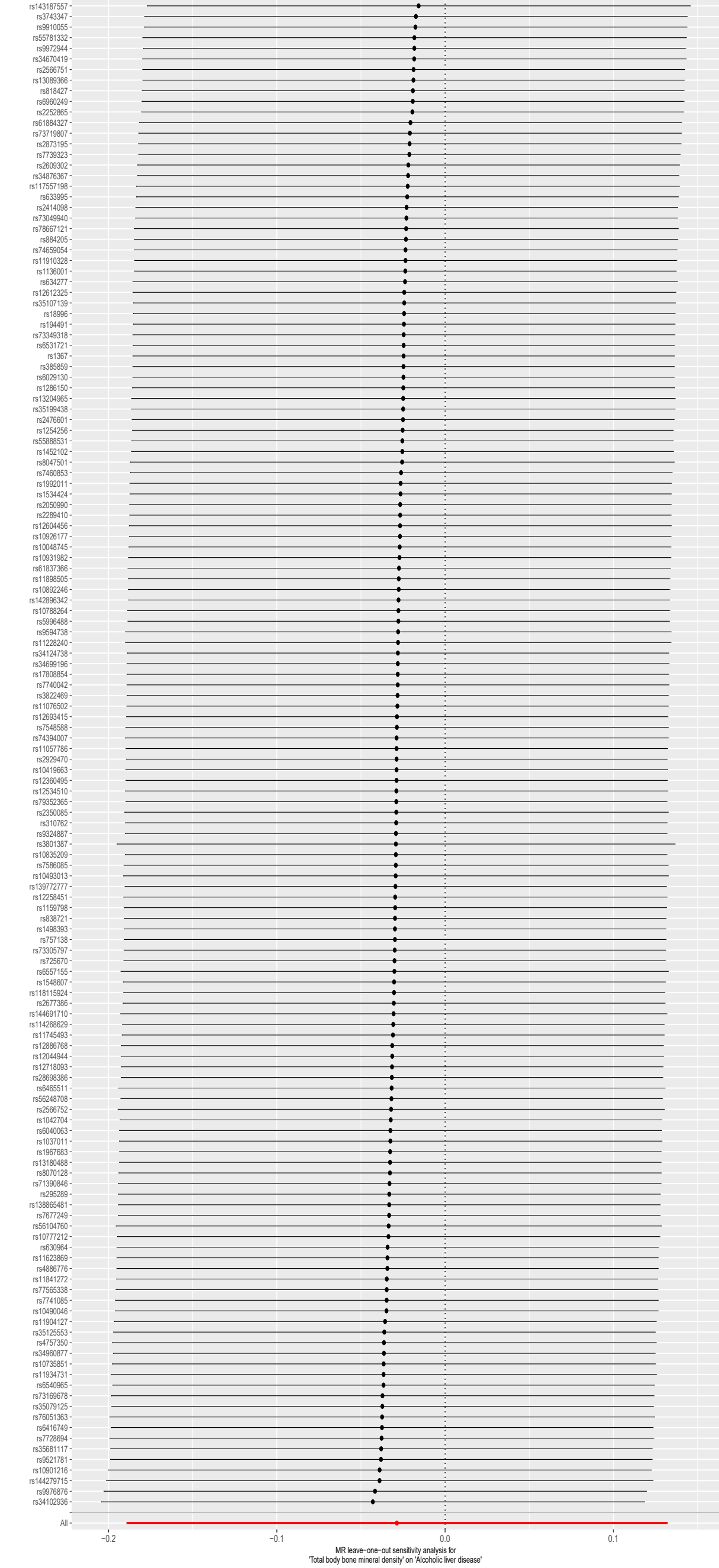

B

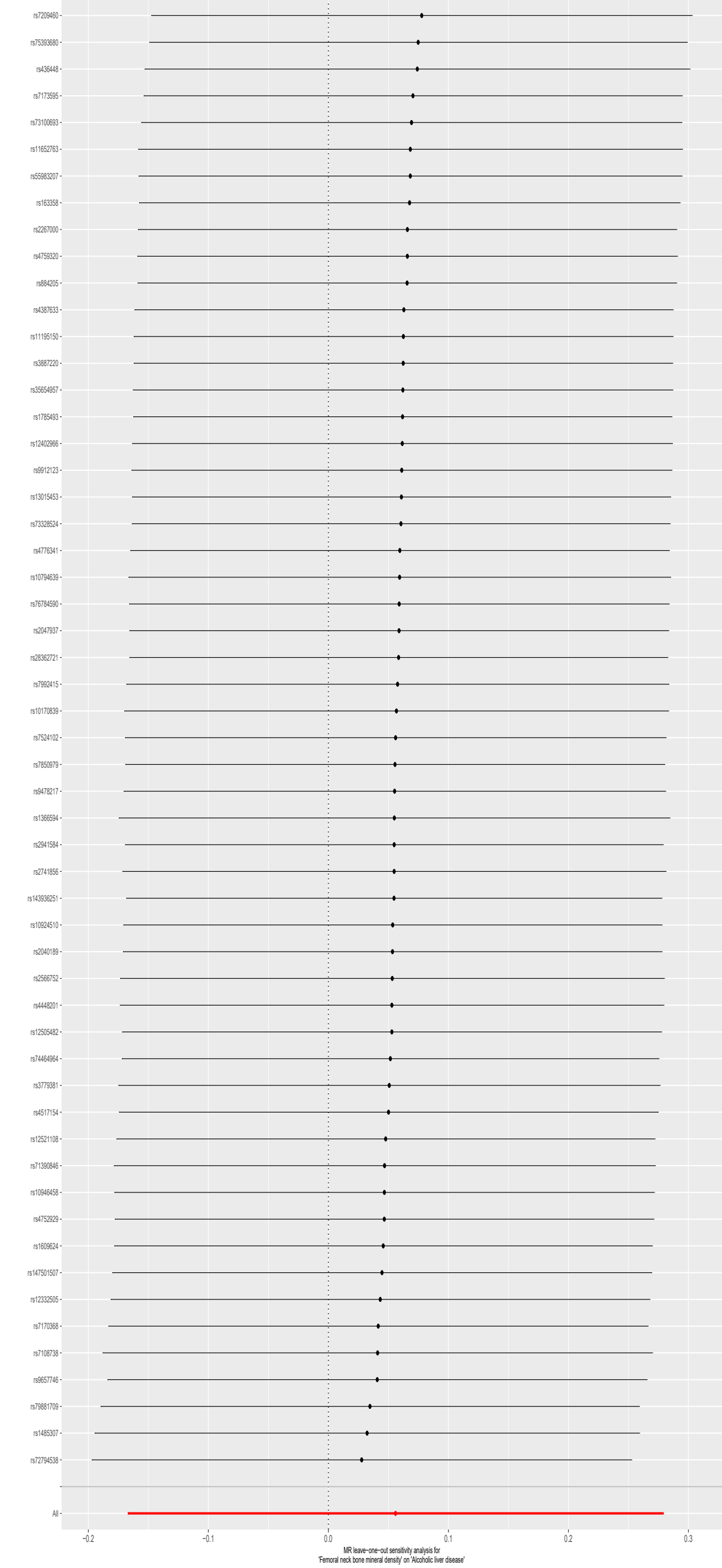

C

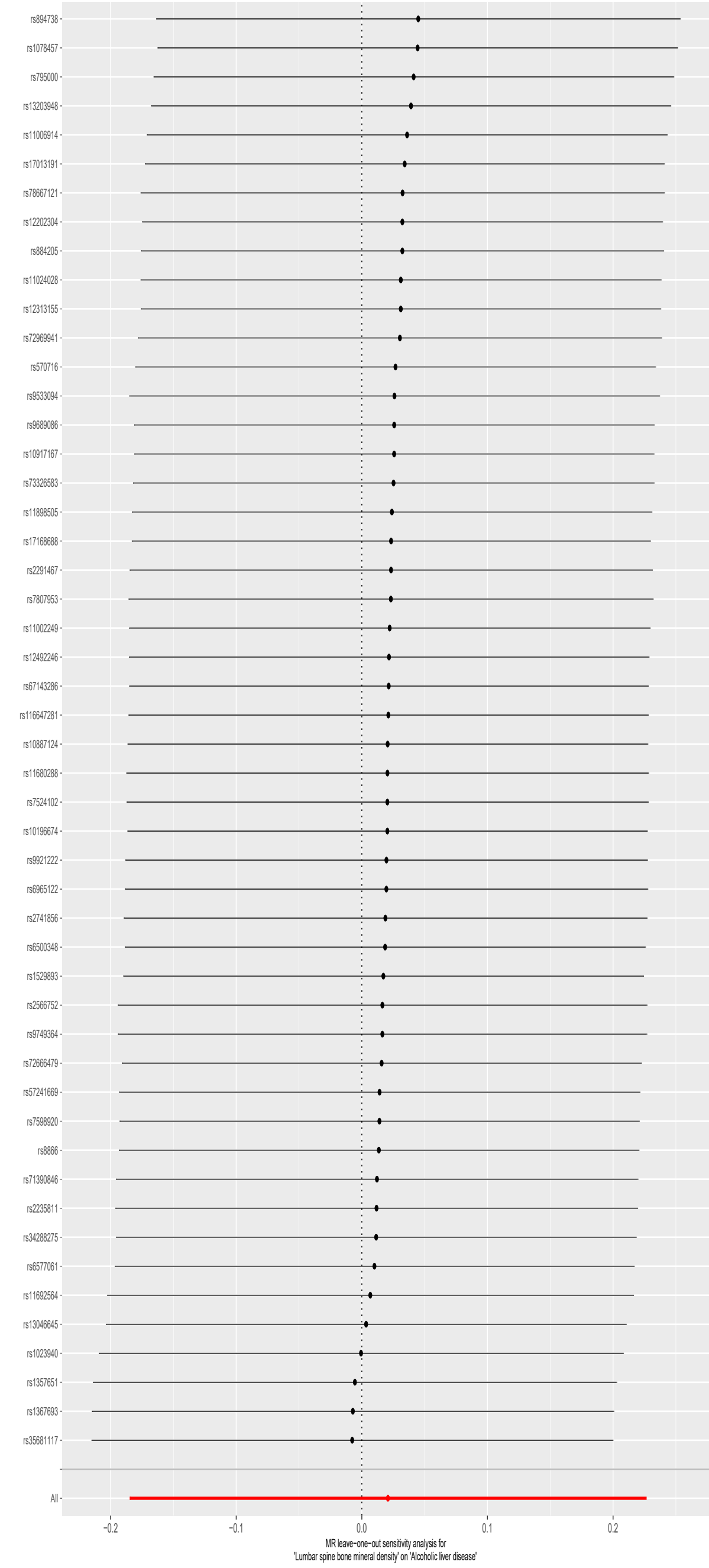

D

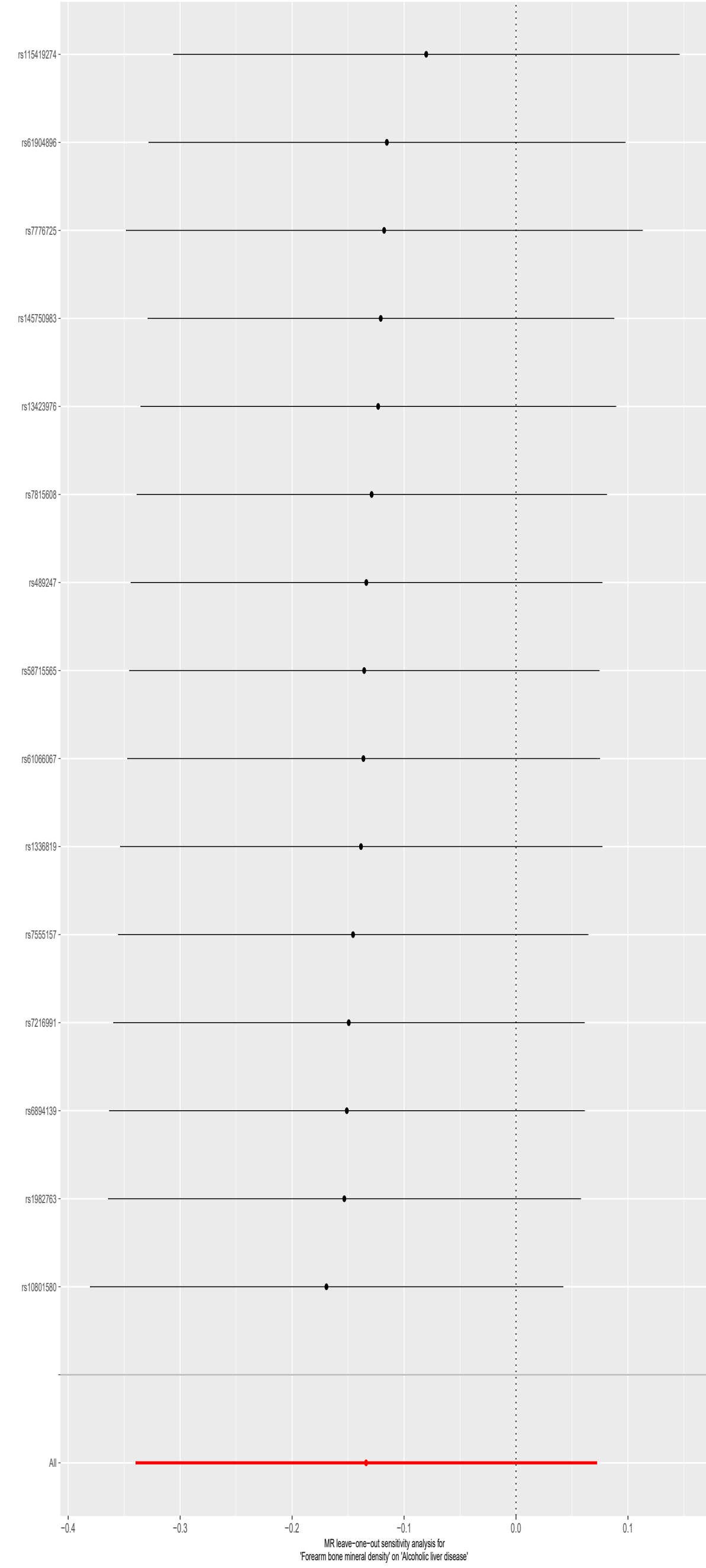

Supplement: S13 Fig — Leave-one-out analysis of A) TB-BMD; B) FN-BMD; C) LS-BMD; D) FA-BMD on ALD. (PDF) [file pone.0292881.s013.pdf]

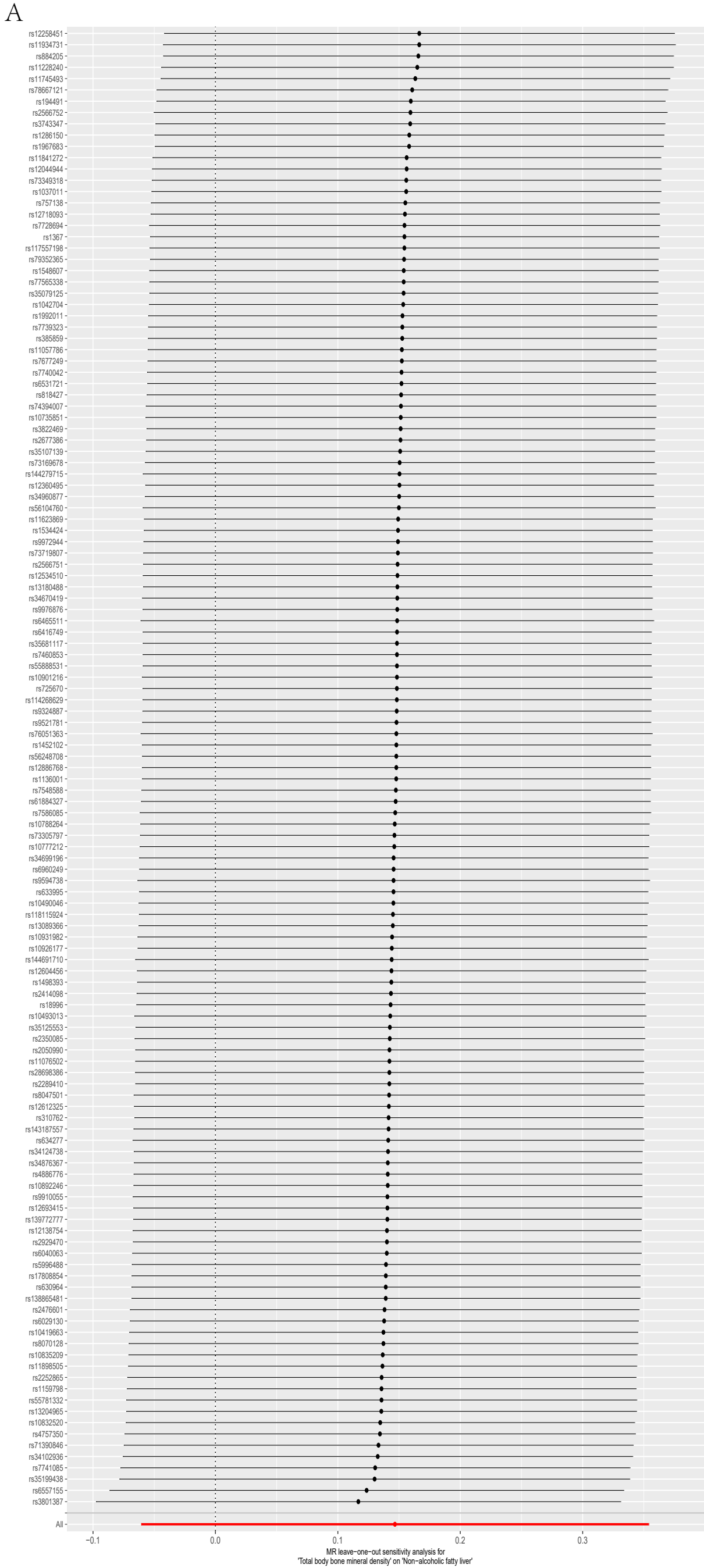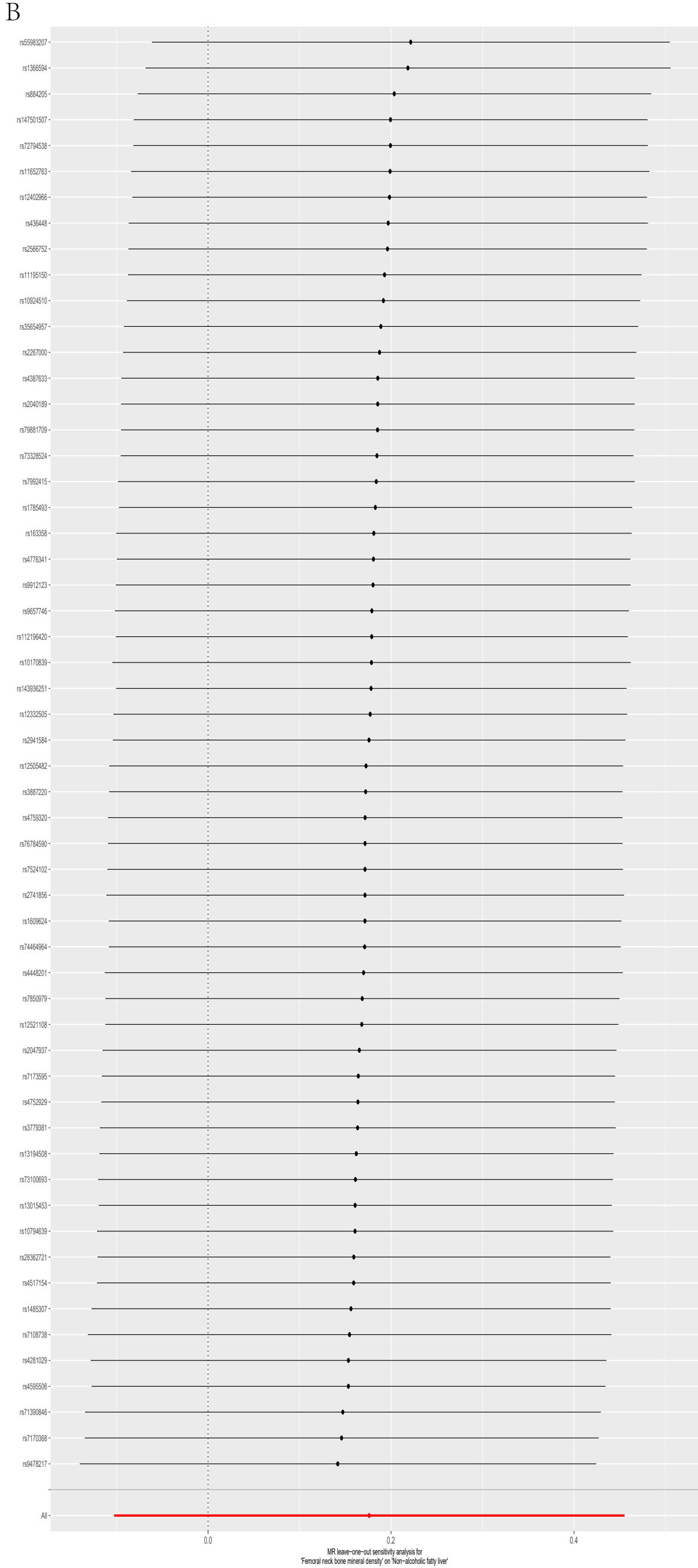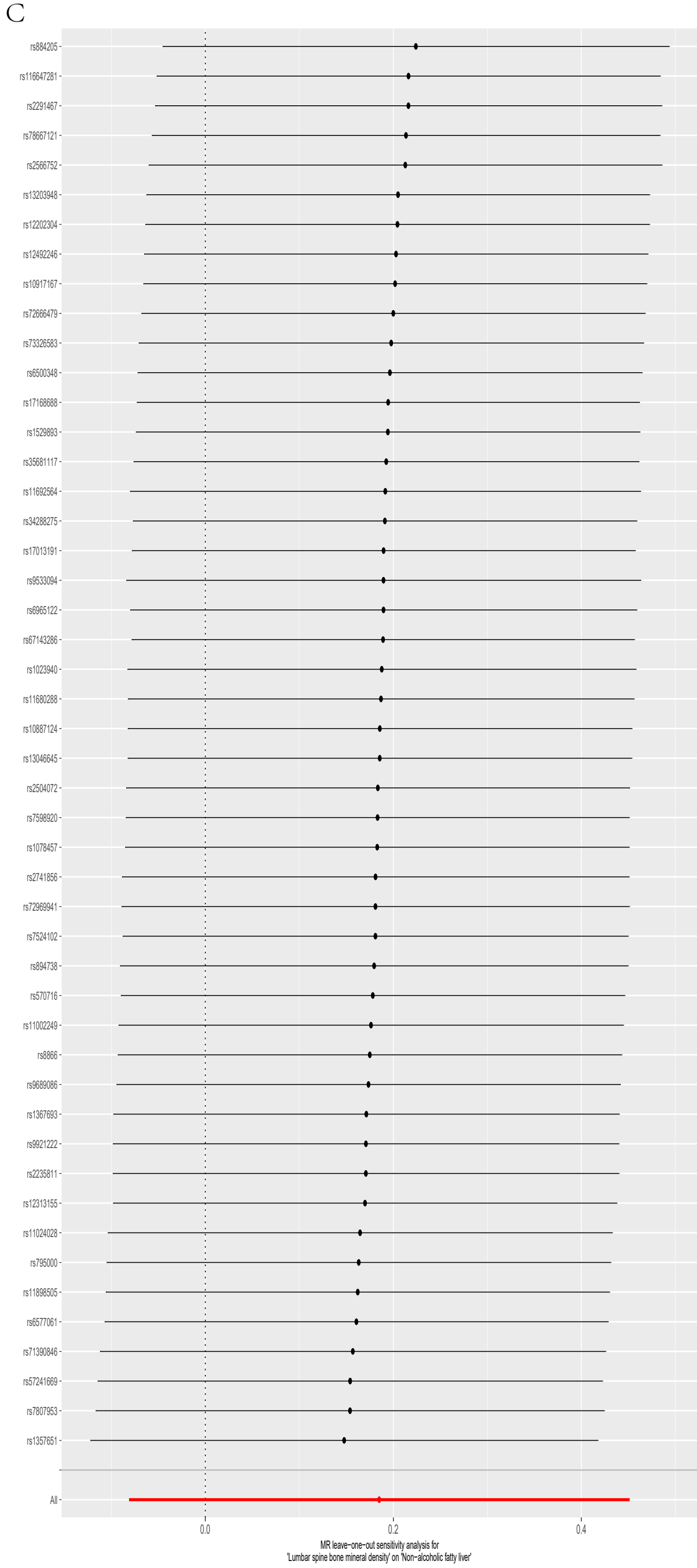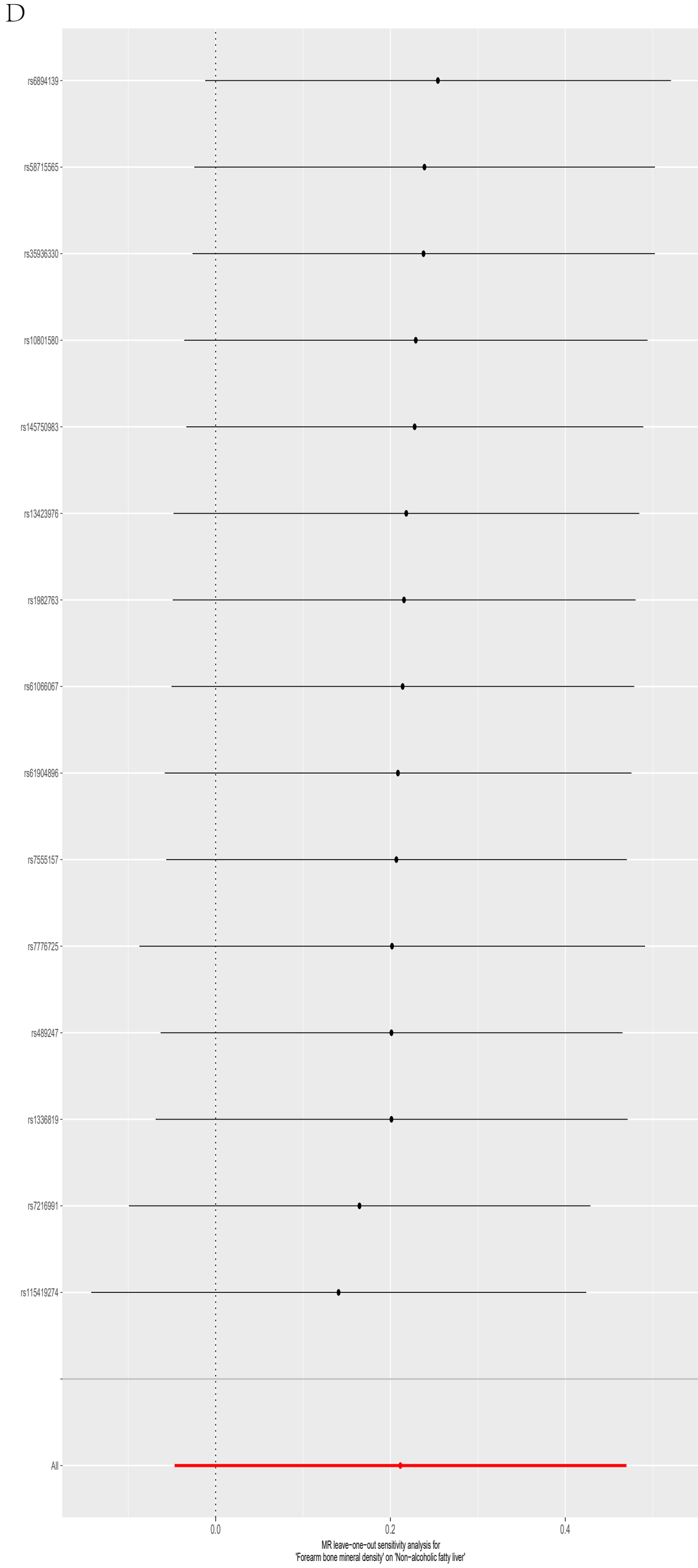

Supplement: S14 Fig — Leave-one-out analysis of A) TB-BMD; B) FN-BMD; C) LS-BMD; D) FA-BMD on NAFLD. (PDF) [file pone.0292881.s014.pdf]
