# Supplementary material for: The associations of alcoholic liver disease and nonalcoholic fatty liver disease with bone mineral density and the mediation of serum 25-Hydroxyvitamin D: A bidirectional and two-step Mendelian randomization
Source: PLoS One. 2023 Oct 19;18(10):e0292881. doi: 10.1371/journal.pone.0292881 (PMC10586666; doi:10.1371/journal.pone.0292881)
Supplement: Attachment — Submitted filename: Response to Reviewers.docx [file pone.0292881.s016.docx]

**Dear Reviewers,**

We would like to express our heartfelt thanks to you for the insightful comments. We have revised the manuscript according to your comments. Please find our point-to-point responses in the following. If there is any other concern, please don’t hesitate to let us know.

**Point-to-point Response**

**Reviewer 1#**

1. Upon reviewing Tables S2 and S3, it becomes evident that some of the variants exhibit unusually high R2 values. As a result, it is imperative for the authors to carefully reassess their calculations and the formulas utilized in the analysis. Such remarkably elevated R2 values may warrant closer scrutiny to ensure the accuracy and reliability of the reported results.

**Answer:** Thank you for your insightful comments. We carefully reviewed Supplementary Tables 2 and 3 and found that, as you said, two genetic variants, rs2294915 and rs738408, showed abnormally high R2 values. Therefore, we carefully checked the data of these two SNPs and found their EAF and beta values were incorrectly filled in Supplementary Tables 2 and 3, resulting in abnormally high R2 values. We have corrected them in the revised manuscript. Meanwhile, the values of other SNPs were also carefully checked to ensure the accuracy of our results. We apology for the careless mistake.

2.Consistency in the number of significant figures throughout the entire manuscript is crucial. Therefore, it is essential to ensure that the chosen number of significant figures remains uniform across all sections and calculations presented in the paper.
For example IVW beta = -0.0288; 95% CI: -0.0488, -0.0087; P = 0.005, if adopting three significant figures, the values should be presented as IVW beta = -0.0288; 95% CI: -0.0488, -0.00870; P = 0.00500. The manuscript currently contains a mix of two, three, and four significant figures, leading to inconsistency in reporting. To rectify this, the authors have the flexibility to choose which format (two, three, or four significant figures) to adopt throughout the paper.

**Answer:** Thanks a lot for your comments. We have revised our manuscript and used three significant figures throughout the manuscript.

**Response to journal**

Please review your reference list to ensure that it is complete and correct. If you have cited papers that have been retracted, please include the rationale for doing so in the manuscript text, or remove these references and replace them with relevant current references. Any changes to the reference list should be mentioned in the rebuttal letter that accompanies your revised manuscript. If you need to cite a retracted article, indicate the article’s retracted status in the References list and also include a citation and full reference for the retraction notice.

**Answer:** Thank you for your comments. We have re-examined the references in our manuscript and did find that some of the references had incomplete information, as you mentioned. We have revised the manuscript accordingly. No references to the retracted paper were cited in our manuscript. In addition, the link to the second reference we cited was accidentally lost, so we have updated the corresponding reference and revised the sentence "affects approximately 200 million individuals worldwide" to "affects approximately 500 million men and women worldwide".
